# Choosing Covariate Balancing Methods for Causal Inference: Practical Insights From a Simulation Study

**DOI:** 10.1002/sim.70672

**Published:** 2026-07-08

**Authors:** Etienne Peyrot, Raphaël Porcher, François Petit

**Affiliations:** ^1^ Université Paris Cité and Université Sorbonne Paris Nord, Inserm, INRAE, Center for Research in Epidemiology and StatisticS (CRESS) Paris France; ^2^ Centre d'Épidémiologie Clinique Assistance Publique‐Hôpitaux de Paris, Hôtel‐Dieu Paris France

**Keywords:** causal inference, inverse probability of treatment weighting, Monte Carlo simulation, observational study, treatment effect estimation

## Abstract

**Background:**

Weighting methods are widely used for confounding adjustment in observational studies, but their finite‐sample behavior depends on implementation choices and empirical overlap. We compare IPTW, just‐ and over‐identified covariate balancing propensity score (CBPS), CBPS by tailored‐loss function (CBPS‐TLF), energy balancing (EB), and kernel optimal matching (KOM).

**Methods:**

We conducted Monte Carlo simulations across 36 main scenarios varying sample size, treatment prevalence, and a complexity factor increasing confounding and reducing overlap. The main simulation considered a null constant treatment effect, with non‐null constant effects examined as sensitivity analyses. Average treatment effects and average treatment effects on the treated were estimated using weighted least squares (WLS) and doubly robust (DR) estimators. Inference followed published recommendations when feasible. An empirical illustration used probitsim.

**Results:**

Performance depended on the estimator and scenario complexity. Under WLS, IPTW and CBPS‐TLF were more sensitive to complexity, while standard CBPS often behaved similarly to IPTW but with less deterioration in some high‐prevalence settings. EB and KOM showed more stable point‐estimation patterns across scenarios. DR estimation reduced differences between weighting methods when all confounders were included in the outcome model, although confidence‐interval performance remained heterogeneous. probitsim results were consistent with simulation patterns.

**Conclusions:**

The study should be read as practical guidance rather than a ranking of methods. It identifies settings where weighting analyses become sensitive to prevalence, overlap, tuning, and variance estimation. Confidence intervals that account for weight construction and tuning remain an important open practical issue.

## 1 Introduction

In observational studies, treatment allocation is not randomized, and causal comparisons require adjustment for baseline differences between treatment groups. Since the seminal works on propensity scores by Rosenbaum and Rubin [1], weighting methods have become a central tool for estimating marginal causal effects from observational data, both in theoretical [2, 3] and applied research [4, 5]. In practice, however, several weighting strategies are available, and choosing among them is not straightforward. Their finite‐sample behavior may depend on treatment prevalence, empirical overlap, confounding strength, and implementation choices.

Among weighting methods, inverse probability of treatment weighting (IPTW), originally proposed by Rosenbaum [6] building upon survey sampling [7], is well‐known and commonly used. IPTW is a natural choice because it is simple, transparent, and familiar to applied researchers. However, it can be inefficient or unstable when estimated propensity scores are close to zero or one, and its performance may be sensitive to propensity‐score specification, treatment prevalence, and empirical overlap. In applied work, careful diagnostics and weight post‐processing, such as truncation, trimming, or stabilization, are often considered to address these issues [8, 9, 10, 11]. These limitations have motivated alternative weighting strategies that construct weights using criteria other than likelihood‐based propensity‐score estimation alone.

One such strategy is covariate balancing propensity score (CBPS), which estimates propensity scores while directly targeting covariate balance [12]. The original CBPS method includes a just‐identified version (CBPS‐JI) and an over‐identified version (CBPS‐OI), both of which estimate propensity scores through covariate‐balancing moment conditions. CBPS‐TLF [13] is related to CBPS, but uses a different construction: instead of solving the standard CBPS moment conditions, it fits the propensity score by optimizing an estimand‐tailored loss function. Including both standard CBPS and CBPS‐TLF therefore helps assess whether observed performance patterns are specific to the tailored‐loss formulation or shared more broadly by CBPS‐based propensity‐score weighting. Other recent methods construct weights without first estimating a propensity score. Energy balancing (EB) targets distributional balance through an energy‐distance criterion [14]. Unlike CBPS or EB, kernel optimal matching (KOM) does not directly target covariate balance, but chooses weights through a worst‐case bias or conditional mean‐squared error criterion in a reproducing kernel Hilbert space (RKHS) [15, 16]. Thus, the methods compared here should be viewed as alternative weighting strategies, not as evidence that IPTW, CBPS, augmented inverse probability weighting, or targeted maximum likelihood estimation cannot address confounding adjustment.

Because these strategies differ in both their statistical motivation and implementation requirements, our comparison focuses on a specific practical question: how do they behave in finite samples under common difficulties faced in applied analyses? The study is not designed primarily around formal positivity violations, nor is it primarily a study of systematic model misspecification. Throughout the simulation study, weak positivity is preserved, but the scenarios are designed to create different levels of empirical overlap difficulty. Similarly, although some limited nuisance‐model misspecification is present because the data‐generating mechanism includes nonlinear terms whereas the fitted nuisance models use simpler specifications, misspecification is not varied as a primary simulation factor. Our goal is instead to study finite‐sample behavior under varying treatment prevalence, empirical overlap, and confounding strength.

This raises the question of how methods with different weighting objectives should be compared. Covariate balance diagnostics are useful because they help detect weighting schemes that may leave residual confounding or produce unstable comparisons. However, balance is a diagnostic tool rather than the final object of interest. Poor balance matters mainly because it may lead to biased or unstable treatment‐effect estimates. Moreover, not all methods target the same notion of balance: some target covariate balance only indirectly through a bias criterion [15, 16], whereas others construct weights by explicitly targeting balance‐related moment or distributional criteria [12, 14]. For this reason, it would be ill‐advised to appraise these methods by a single balance metric [17]. Instead, we assess them by the task that motivates their use in applications: estimating marginal causal effects.

Accordingly, we focus on the average treatment effect (ATE) and the average treatment effect on the treated (ATT), two estimands used in the source literature for the methods under review. For each weighting method, we consider two recurring estimators: a weighted least squares (WLS) estimator and an augmented inverse probability weighting type, or doubly robust (DR), estimator. The DR estimator is included not primarily to study robustness under misspecification, but because outcome‐augmented estimators are used in parts of the source literature for the methods considered here [15] and are central in the AIPW literature [18, 19]. Moreover, under standard regularity conditions and correctly specified nuisance functions, augmented inverse probability weighting type estimators are closely related to efficient influence‐function or one‐step estimators and can attain the semiparametric efficiency bound [3, 20]. Although such estimators also have a double‐robustness property, systematic misspecification is not the main focus of this study. We also do not include full estimation frameworks or regression‐based nuisance‐modeling strategies, such as targeted maximum likelihood estimation (TMLE) [21], g‐computation with flexible outcome models, splines, or regularized regression approaches, as separate methods in the main comparison, because our objective is to compare weighting schemes rather than complete treatment‐effect estimation procedures. In addition, EB and KOM do not estimate propensity scores, so they cannot be inserted into a standard TMLE framework in the same direct way as IPTW, CBPS, or CBPS‐TLF.

Finally, applied usefulness depends not only on point‐estimation performance, but also on uncertainty quantification. This is important because confidence‐interval construction is less standardized, less direct, or less practical for some newer weighting methods, especially when the weights are obtained through complex optimization, regularization, or kernel‐based procedures. We therefore report empirical coverage of nominal 95% confidence intervals and the variance ratio, defined as the average estimated variance divided by the empirical Monte Carlo variance of the point estimates. Coverage evaluates whether confidence intervals contain the true effect at the expected frequency, whereas the variance ratio helps assess whether the estimated variances are calibrated. Used together with bias and empirical variance, these quantities provide a more complete description of confidence‐interval performance.

Our objective is not to provide a definitive ranking of weighting methods according to a single criterion. Instead, we aim to identify practical strengths, limitations, and failure modes across scenarios, including sensitivity to treatment prevalence and empirical overlap, bias‐variance trade‐offs, tuning burden, implementation accessibility, and confidence‐interval construction. We emulate how a practicing biostatistician might proceed, that is, following author guidance, avoiding extensive hyperparameter tuning beyond routine feasibility, and favoring transparent and reproducible choices.

This paper is structured as follows: we first introduce the weighting methods and the estimators in Section 2. Then, in Section 3, we detail the data‐generating mechanism for the Monte Carlo simulation. In Section 4, we present the results of our simulations. In Section 5, we evaluate the methods on probitsim, a synthetic observational study built by calibrating a simulated patient cohort to real‐world clinical summaries while preserving a known data‐generating mechanism. Finally, in Section 6, we conclude with a discussion of the practical limitations and challenges identified in our simulations.

## 2 Statistical Setting

### 2.1 Causal Framework

We adopt the Neyman‐Rubin potential outcomes framework [22, 23]. For each unit, let Y(0) and Y(1) denote the potential outcomes under treatment A=0 and A=1, respectively, and let $X\in 𝒳\subset {\mathbb{R}}^{p}$ be a vector of baseline covariates. Conceptually, each unit is associated with the tuple (X,Y(0),Y(1),A), while only (X,A,Y) is observed. We assume the following:

- **Stable Unit Treatment Value Assumption.** No interference and no hidden versions of treatment; consequently,
Y=AY(1)+(1−A)Y(0).
- **Unconfoundedness.** No unmeasured confounding given X:
{Y(0),Y(1)}╨A|X.
- **Positivity (weak overlap).** For all x in the support of X,
0<Pr(A=1|X=x)<1.

These assumptions are sufficient for identifying both the ATE and the ATT. For the ATT, however, weaker conditions are sufficient: because Y(1) is observed among treated units, identification only requires exchangeability for the untreated potential outcome, that is, Y(0)╨A|X, together with overlap for the control group on the treated support, meaning that Pr(A=1|X=x)<1 for covariate values x occurring among treated units.

### 2.2 Estimation and Notation

We observe an i.i.d. sample ${\left(X_{i},Y_{i},A_{i}\right)}_{i=1}^{n}$. We focus on two causal estimands that the original authors used to demonstrate their weighting methods: the average treatment effect (ATE) and the average treatment effect on the treated (ATT).

ATE:=𝔼[Y(1)−Y(0)],ATT:=𝔼[Y(1)−Y(0)|A=1].

Both estimands are marginal additive effects; for binary outcomes, they correspond to risk differences. Although ATE is most frequently reported in applied studies, we also evaluate ATT because several of the methods we study were originally developed for ATT and later adapted to ATE [15, 16]. We do not consider the average treatment effect on the controls (ATC), which was not emphasized by the methods under review and is methodologically analogous to the ATT (interchanging treatment and control) and therefore expected to behave similarly.

We denote by n the sample size and by $N_{0}=\sum_{i}(1-A_{i})$ and $N_{1}=\sum_{i}A_{i}$ the sizes of the control and treated groups, respectively.

### 2.3 Estimators

In this section, we briefly describe the two estimators used in this paper, namely the weighted least squares (WLS) estimator and a doubly robust (DR) estimator. We chose these estimators because they are used in the source literature introducing or applying several of the weighting methods compared in this study. Both estimators require a set of weights ${\left(W_{i}\right)}_{i}$ computed beforehand by a weighting method. For each estimand, the weights and the estimator must target the same population of interest: the general population for the ATE and the treated population for the ATT. This section defines the point estimators and highlights general inference issues. Because valid uncertainty quantification depends on how the weights are constructed, the method‐specific variance estimators and confidence‐interval procedures are described in Section 2.4.

#### 2.3.1 Weighted Least Squares Estimator

The WLS estimator considered here is the coefficient of the treatment indicator in a weighted least‐squares regression of the observed outcome on an intercept and the treatment indicator. Equivalently, and more importantly for interpretation, this coefficient is a weighted contrast between the observed outcome means in the treated and untreated groups. With weights ${\left(W_{i}\right)}_{i}$ computed beforehand, the estimator can be obtained by solving 


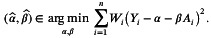


The WLS estimator is then given by ${\hat{\beta }}_{\text{WLS}}$. Although this implementation is often described as a weighted linear regression of the outcome on the treatment indicator and an intercept, it should not be interpreted as specifying a Gaussian outcome model for the binary outcome. In this setting, the regression is only a computational device for estimating a weighted marginal mean contrast.

Indeed, the coefficient assigned to the treatment indicator is algebraically equal to the weighted difference in group means:

$${\hat{\beta }}_{\text{WLS}}=\frac{\sum_{i=1}^{n}A_{i}W_{i}Y_{i}}{\sum_{i=1}^{n}A_{i}W_{i}}-\frac{\sum_{i=1}^{n}(1-A_{i})W_{i}Y_{i}}{\sum_{i=1}^{n}(1-A_{i})W_{i}}.$$

When the weights are normalized within treatment groups, so that $\sum_{i=1}^{n}A_{i}W_{i}=\sum_{i=1}^{n}(1-A_{i})W_{i}=1$, this contrast can be written as

$${\hat{\beta }}_{\text{WLS}}=\overset{n}{\underset{i=1}{\sum }}(2A_{i}-1)W_{i}Y_{i}.$$

Thus, if the weights target the ATE, the WLS coefficient estimates an ATE‐type weighted mean contrast; if the weights target the ATT, it estimates an ATT‐type weighted mean contrast. For binary outcomes, the weighted group means are weighted proportions, and the WLS coefficient is therefore a weighted risk difference. When the weights are normalized inverse‐probability weights, this estimator corresponds to the Hájek form of the IPTW estimator [24].

The usual model‐based standard errors reported by a weighted least‐squares regression should not be interpreted as valid standard errors for the causal weighted mean contrast. Valid uncertainty quantification must account for the variance structure induced by weighting. When weights depend on an estimated propensity score, it should also account for propensity‐score estimation uncertainty; when weights depend on data‐driven tuning or hyperparameter selection, this additional source of variability may also be relevant. Depending on the weighting method, appropriate approaches can include stacked M‐estimation, linearization, sandwich variance estimators, or bootstrap procedures [2, 20, 25, 26, 27]. The procedures used for each weighting method in this study are described in Section 2.4.

#### 2.3.2 Doubly Robust Estimator

We also considered a doubly robust (DR) estimator as an outcome‐augmented alternative to the WLS weighted mean contrast. The DR estimator combines the weighting scheme with estimates of the response surfaces $\mu_{0}(X_{i}):=𝔼\left[Y_{i}(0)|X_{i}\right]$ and $\mu_{1}(X_{i}):=𝔼\left[Y_{i}(1)|X_{i}\right]$. It is included because outcome augmentation can improve efficiency and reduce sensitivity to imperfect weighting when the outcome‐regression model is correctly specified, or at least sufficiently accurate. Comparing WLS and DR estimators is also relevant because both types of estimators are used in the methodological literature introducing or applying several of the weighting methods considered in this study.

For the ATE and ATT, the DR estimators considered in this study are given by

$$\begin{array}{ll} \hat{\text{ATE}} & =\underset{i}{\sum }\left[\frac{1}{n}\left\{{\hat{\mu }}_{1}(X_{i})-{\hat{\mu }}_{0}(X_{i})\right\}+A_{i}W_{i}\left\{Y_{i}-{\hat{\mu }}_{1}(X_{i})\right\}\right.\\ & \;\left.-(1-A_{i})W_{i}\left\{Y_{i}-{\hat{\mu }}_{0}(X_{i})\right\}\right],\\ \hat{\text{ATT}} & =\frac{1}{N_{1}}\underset{i}{\sum }A_{i}\left\{Y_{i}-{\hat{\mu }}_{0}(X_{i})\right\}-\underset{i}{\sum }(1-A_{i})W_{i}\left\{Y_{i}-{\hat{\mu }}_{0}(X_{i})\right\}, \end{array}$$

where ${\hat{\mu }}_{0}$ and ${\hat{\mu }}_{1}$ are estimators of $\mu_{0}$ and $\mu_{1}$, respectively. If the weights used are those obtained through IPTW, this estimator corresponds to the augmented inverse probability weighting (AIPW) estimator [2, 3, 18, 19, 20]. AIPW and related DR estimators are closely connected to efficient influence‐function or one‐step estimators and can be locally efficient under correct nuisance‐function specification and standard regularity conditions [3, 20].

The term “doubly robust” refers to consistency of the point estimator, not automatically to valid uncertainty quantification. Under the usual causal assumptions, a DR point estimator can remain consistent if either the treatment‐assignment component or the outcome‐regression component is correctly specified. However, confidence intervals must still account for nuisance estimation, including estimation of the propensity score or other weighting quantities and estimation of the outcome regressions. For weighting methods such as EB and KOM, inference may also need to account for uncertainty introduced by the weight‐construction procedure itself, including optimization choices and, when relevant, tuning or hyperparameter selection. Thus, DR estimation should not be interpreted as solving the inference problem by itself. The confidence‐interval procedures used for each weighting method are described in Section 2.4.

### 2.4 Weighting Methods

In this section, we briefly describe the weighting methods compared in our study and the confidence‐interval procedures used with each method. We use the term weighting methods because not all approaches considered here are defined by conventional covariate‐balance constraints. IPTW and CBPS construct weights through a propensity‐score model, CBPS‐TLF constructs propensity‐score weights through an estimand‐tailored loss, EB directly targets covariate balance by minimizing an energy‐distance criterion between the weighted covariate distribution and the target covariate distribution. In contrast, KOM constructs weights by minimizing a worst case bias‐variance, or CMSE, criterion in an RKHS. We include IPTW as the primary reference method, together with standard CBPS, EB, KOM, and CBPS‐TLF [1, 12, 13, 14, 15, 16]. The weights produced by each method are then used with the WLS and DR estimators defined in Section 2.3.

#### 2.4.1 IPTW

Inverse probability of treatment weighting (IPTW) is a widely used propensity‐score weighting method [1, 2, 6]. It assigns weights inversely proportional to the probability of receiving the observed treatment. Let e(X)=ℙ(A=1|X) denote the propensity score and let ê(X) be its estimate. In this article, we use rescaled versions of the conventional IPTW weights. For the ATE, the weights are

$$W_{i}^{\text{ATE}}=\frac{1}{n}\left\{\frac{A_{i}}{ê(X_{i})}+\frac{1-A_{i}}{1-ê(X_{i})}\right\}.$$

For the ATT, the corresponding observation‐level weights can be written as

$$W_{i}^{\text{ATT}}=\frac{1}{N_{1}}\left\{A_{i}+(1-A_{i})\frac{ê(X_{i})}{1-ê(X_{i})}\right\}.$$

For notational consistency across weighting methods, the scaling constants are incorporated into the IPTW weights rather than into the estimator. When these weights are combined with the WLS or DR expressions above, they recover the usual normalized IPTW and AIPW estimators of the ATE and ATT.

For IPTW, we estimate the propensity score with a logistic regression model. This is a smooth finite‐dimensional nuisance model, for which standard stacked M‐estimation is a natural approach to variance estimation. The estimating equations can stack the score equations for the propensity‐score model with the estimating equation for the treatment‐effect estimator, yielding a sandwich variance estimator and a Wald‐type confidence interval [20, 25, 26, 27]. This construction is especially direct for parametric IPTW because the nuisance component is finite dimensional and differentiable. The extensions and complications that arise for optimization‐based, kernel‐based, or regularized weighting methods are described in the corresponding method‐specific subsections below.

#### 2.4.2 Standard Covariate Balancing Propensity Score

The standard covariate balancing propensity score (CBPS) estimates a propensity‐score model while directly targeting covariate balance [12]. Imai and Ratkovic consider a logistic propensity‐score model and use the dual role of the propensity score as both a treatment‐assignment model and a balancing score. The original article proposes two main variants: a just‐identified version (CBPS‐JI) and an over‐identified version (CBPS‐OI). Once the propensity score has been estimated, ATE and ATT weights are obtained using the IPTW formulas in Section 2.4.1.

##### 2.4.2.1 Just‐Identified CBPS (CBPS‐JI)

The just‐identified version estimates the propensity‐score parameters using covariate‐balancing moment conditions. For the ATE, the parameters are chosen so that

$$\overset{n}{\underset{i=1}{\sum }}\left\{\frac{A_{i}}{e_{\beta }(X_{i})}-\frac{1-A_{i}}{1-e_{\beta }(X_{i})}\right\}X_{i}=0.$$

For the ATT, the corresponding balance condition targets the treated population:

$$\overset{n}{\underset{i=1}{\sum }}\left\{A_{i}-(1-A_{i})\frac{e_{\beta }(X_{i})}{1-e_{\beta }(X_{i})}\right\}X_{i}=0,$$

up to a multiplicative constant that does not affect the root of the estimating equation. Thus, CBPS‐JI chooses propensity‐score parameters so that the implied weights directly balance the covariates for the estimand of interest. It is just identified because the number of moment conditions matches the number of propensity‐score parameters.

For confidence intervals, CBPS‐JI fits naturally into the stacked M‐estimation framework used for parametric propensity‐score weighting. In our study, the estimating equations for the treatment‐effect estimator were stacked with the CBPS estimating equations. For the DR estimator, the outcome‐regression score equations were also included. This accounts for uncertainty due to propensity‐score estimation, under the regularity conditions for smooth finite‐dimensional estimating equations.

##### 2.4.2.2 Over‐Identified CBPS (CBPS‐OI)

The over‐identified version augments the balancing moment conditions with the score equations from the treatment‐assignment model. For a logistic propensity‐score model, this gives a generalized method of moments estimator [28] of the form


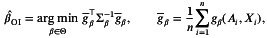


where

$$g_{\beta }(A_{i},X_{i})=\left(\begin{array}{l} s_{\beta }(A_{i},X_{i})\\ w_{\beta }(A_{i},X_{i})X_{i} \end{array}\right).$$

Here, $s_{\beta }(A_{i},X_{i})$ is the score contribution of the propensity‐score model, and $w_{\beta }(A_{i},X_{i})$ is the balancing weight corresponding to the target estimand. CBPS‐OI therefore combines model fit and covariate balance. The over‐identifying restrictions can also be assessed using Hansen's J‐test, providing a specification check for the propensity‐score model [12].

Inference for CBPS‐OI is less direct than for CBPS‐JI. Because CBPS‐OI is obtained from an over‐identified GMM objective rather than from a simple just‐identified system of estimating equations, the direct stacked M‐estimation procedure used for CBPS‐JI does not apply without further derivation. In our implementation, confidence intervals for CBPS‐OI were computed conditionally on the estimated weights; the additional uncertainty induced by the GMM‐based propensity‐score estimation was not propagated analytically. This is consistent with the public WeightIt implementation, for which M‐estimation is supported for just‐identified CBPS but not for the over‐identified version. It also matches the way the public CBPS [29] implementation was used in this simulation, namely as a weight‐estimation step rather than through package‐specific outcome‐variance routines. This limitation is considered when interpreting coverage results.

#### 2.4.3 Covariate Balancing Propensity Score by Tailored Loss Functions

Covariate balancing propensity score by tailored loss functions (CBPS‐TLF) [13] estimates the propensity score by optimizing a loss function tailored to the target estimand. As in IPTW and standard CBPS, the resulting propensity‐score estimate is then transformed into ATE or ATT weights using the formulas in Section 2.4.1. The method is related to standard CBPS because both approaches connect propensity‐score estimation with covariate balance. However, whereas standard CBPS imposes explicit covariate‐balancing moment conditions, CBPS‐TLF chooses a loss function whose first‐order conditions correspond to balance conditions relevant to the target estimand and link function. We therefore view CBPS‐TLF as an extension of the CBPS principle, rather than as a method that is uniformly preferable to standard CBPS.

Concretely, CBPS‐TLF models the propensity score e(x) with a generalized linear model (GLM) having link l, and a prespecified feature set ℱ for the linear predictor (e.g., main effects, interactions, polynomials, or splines). The choice of estimand and link induces a score S. The propensity function is then obtained by solving 

(1)

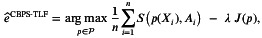


with $𝒫=l^{-1}(ℱ)$, J a regularizer to limit overfitting, λ a parameter controlling the degree of regularization, and S a proper scoring rule whose form depends on the estimand of interest and the GLM link function l.

If l is the logit link function, then the expressions of S for the estimands of interest in our study are as follows. Let (q,a)∈(0,1)×{0,1}:

- ATE: $S(q,a)=(2a-1)\log\;\left(\frac{q}{1-q}\right)-\frac{a}{q}-\frac{1-a}{1-q}$,
- ATT: $S(q,a)=(1-a)\log\;\left(\frac{1-q}{q}\right)-\frac{a}{q}$.

The maximizer of (1) estimates the propensity score, from which weights are computed using the standard IPTW formulas, and optionally, group‐normalized so that $\sum A_{i}W_{i}=\sum (1-A_{i})W_{i}=1$.

Analytic variance estimation is more difficult for CBPS‐TLF than for smooth finite‐dimensional propensity‐score models fitted by logistic regression or by just‐identified estimating equations. The tailored‐loss objective, regularization, kernel choice, bandwidth choice, and regularization‐parameter selection all affect the final weights. The original authors recommend an honest, design‐based confidence interval that starts from a usual Wald interval and adds a worst‐case bias allowance derived under an RKHS smoothness assumption on the outcome regression [13]. This approach requires specifying the RKHS and an upper bound on the norm of the outcome regression.

In our study, we did not use this honest, design‐based procedure. Instead, confidence intervals for CBPS‐TLF were constructed using robust sandwich standard errors obtained from a stacked M‐estimation system, in the same spirit as for IPTW. This approach accounts for estimation of the propensity‐score model used to construct the weights, but does not propagate uncertainty due to kernel choice, bandwidth selection, or regularization‐parameter selection.

#### 2.4.4 Energy Balancing

Energy balancing aims at minimizing a discrete version of the energy distance [30] between the empirical weighted multivariate cumulative distribution function (CDF) of each treatment group and the empirical multivariate CDF of the target population [14]. This approach generates the following weights: 

(2)

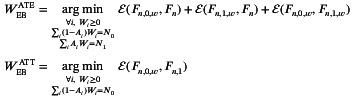


where $F_{n,1}$ (resp. $F_{n}$) denotes the empirical CDF of the treated group (resp. general population); $F_{n,0,w}$ (resp. $F_{n,1,w}$) represents the weighted empirical CDF of the control group (resp. treated group); and ℰ is the empirical energy distance on weighted CDF.

The third term in the minimization problem for the ATE (Equation 2) is a trick proposed by the authors to enhance the performance of their method by further reducing the heterogeneity of the weighted groups at the price of slightly increasing the distance between each group and the target population. Weights obtained through this balancing method are then group‐normalized to satisfy $\sum_{i}A_{i}W_{i}=\sum_{i}(1-A_{i})W_{i}=1$.

The authors recommend bootstrapping to estimate the confidence intervals.

#### 2.4.5 Kernel Optimal Matching

General optimal matching (GOM) aims at finding weights that minimize the worst‐case conditional mean‐squared error (CMSE) criterion for a treatment‐effect estimator over a prespecified class of functions [15, 16]. This min‐max view addresses the fact that the true response surfaces are unknown by minimizing a worst‐case upper bound on CMSE over plausible class of outcome‐regression functions.

Kernel Optimal Matching is an instance of GOM that uses an RKHS as the function class. The advantages of KOM are that an RKHS is flexible enough to be reasonably close to the true response surfaces while resulting in a solvable min‐max optimization problem. The general formula to compute weights for the ATE and ATT via KOM is: 


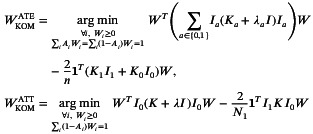


where $K,K_{0},K_{1}$ are the Gram matrices of the chosen kernels $𝒦,{𝒦}_{0},{𝒦}_{1}$ respectively, where $K_{ij}=𝒦(X_{i},X_{j})$, and $I_{a}=\text{diag}(1\left(A_{i}=a\right))$ with 1(·) denotes the indicator function. Finally, $\lambda_{0}$ and $\lambda_{1}$ control the trade‐off between bias and variance in the control and treated groups, respectively.

The authors do not provide an unconditional variance estimate for the estimators they developed. Instead, they provide a conditional variance estimate, given the dataset. This implies that the weights and regularization parameters, being derived from the data, are treated as constants. The procedure to estimate a confidence interval differs between the first paper [15] and the second paper [16]. Because the second paper is more recent and generalizes beyond ATT estimation, we adopt its approach. It consists in building a robust Wald CIs from stacked M‐estimation [31] without accounting for weight uncertainty. The variance of the DR estimator is discussed only in the first article [15] as the authors do not recommend this estimator in the second article [16] due to practical violations of the positivity assumption and the potential for high bias if the outcome and treatment models are misspecified [32].

## 3 Simulation Study Plan

Our objective is to provide a pragmatic review of recently proposed weighting approaches alongside IPTW. We aim to characterize the performance these methods achieve when applied as a practicing biostatistician would: in good faith, following the authors' published recommendations, and without hyperparameter tuning beyond what is typically feasible in routine analyses.

To this end, we use a simple, clinically inspired data‐generating mechanism. Covariates are predominantly categorical and exhibit some correlation, reflecting common features of electronic health records and clinical registries. We vary sample size, treatment prevalence (inducing imbalance in sample sizes between treated and control groups), and a general “complexity” factor that strengthens confounding and reduces overlap by increasing the influence of covariates on both treatment assignment and outcome. This design allows us to probe where methods are robust and where they fail, rather than to engineer settings in which any one method excels.

The primary goal is to document practical shortcomings, such as sensitivity to prevalence, instability under higher complexity, and systematic bias, under defensible, author‐guided implementations. Beyond characterization, the study serves as decision support by linking failure modes to observable data features (treatment prevalence, overlap, and covariate complexity), it offers practical guidance for method selection under routine analytical constraints. We report performance summaries (RMSE, MAE, bias, variance, empirical coverage of 95% confidence intervals, and variance ratios along their Monte Carlo standard errors) primarily to highlight limits and failure modes, providing a realistic picture of what these methods deliver on data that are simple yet share characteristics of clinical practice.

### 3.1 Data‐Generating Mechanism

The data‐generating mechanism described in this section simulates baseline covariates, potential outcomes, and the treatment assignment mechanism. To reflect characteristics of real medical data, the simulated data include more categorical than numerical covariates and incorporate correlations between covariates. For simplicity, the main simulation uses a null constant treatment effect. Additional constant treatment effects of 0.05 and 0.10 are used as a sensitivity analysis. Heterogeneous treatment effects are not considered in this study.

For each patient, the first step is to generate a Gaussian vector $\tilde{X}=({\tilde{X}}^{\left(1\right)},{\tilde{X}}^{\left(2\right)},\ldots ,{\tilde{X}}^{\left(10\right)})$ drawn from a multivariate Gaussian distribution with mean zero and a specified covariance matrix. Each component of the Gaussian vector has variance 1, and the covariance is set to zero for all pairs of variables except for the following:

$$\begin{array}{ll} \text{Cov}\left({\tilde{X}}^{\left(1\right)},\;{\tilde{X}}^{\left(5\right)}\right) & =\text{Cov}\left({\tilde{X}}^{\left(3\right)},\;{\tilde{X}}^{\left(8\right)}\right)=0.2\;,\\ \text{Cov}\left({\tilde{X}}^{\left(2\right)},\;{\tilde{X}}^{\left(6\right)}\right) & =\text{Cov}\left({\tilde{X}}^{\left(4\right)},\;{\tilde{X}}^{\left(9\right)}\right)=0.9\;. \end{array}$$

The following transformation is applied to $\tilde{X}$ to get mixed continuous and binary covariates:

$$X^{\left(i\right)}=\left\{\begin{array}{l} {\tilde{X}}^{\left(i\right)}\;\text{if}\;i\in \{2,4,7,10\},\\ 1\left({\tilde{X}}^{\left(i\right)}>0\right)\;\text{otherwise}. \end{array}\right.$$

Given the data‐generating mechanism for X, we generate the potential outcomes Y(0),Y(1) and the treatment assignment mechanism A as follows:

$$\begin{array}{ll} A & ∼\text{Bernoulli}\left\{{\text{logit}}^{-1}\left[b_{0}+g\left(Xb+\frac{1}{2}X^{\left(1\right)}{X^{\left(2\right)}}^{2}\right)\right]\right\},\\ Y(t) & ∼\text{Bernoulli}\left\{\tau t+{\text{logit}}^{-1}\left[a_{0}+g\left(Xa+\frac{1}{2}X^{\left(3\right)}{X^{\left(4\right)}}^{2}\right)\right]\right\},\\ & \;t\in \{0,1\} \end{array}$$

where τ∈{0,0.05,0.10} is the constant treatment effect, and

$$\begin{array}{ll} a & ={\left(0.9,\;-1.08,\;-2.19,\;-0.6,\;0,\;0,\;0,\;0.71,\;-0.19,\;0.26\right)}^{⊤},\\ b & ={\left(0.8,\;-0.25,\;0.6,\;-0.4,\;-0.8,\;-0.5,\;0.7,\;0,\;0,\;0\right)}^{⊤}. \end{array}$$

The constants $a_{0}$ and $b_{0}$ control the number of events and the proportion of treated respectively. The variable g scales the parts controlled by baseline covariates in both formulas.

From this data‐generating mechanism, we created several scenarios by calibrating $a_{0}$, $b_{0}$, τ, and g in order to:

1. set the probability of an event occurring (i.e., ℙ(Y=1)) to 25% in all scenarios;
2. create scenarios with low, moderate, and high proportions of treated with ℙ(A=1) set to 25%, 50%, and 75% respectively;
3. use a null treatment effect for the main simulation, and repeat the same data‐generating settings with constant treatment effects 0.05 and 0.10 as sensitivity analysis;
4. create scenarios with low, moderate, and high levels of confounding, with the bias of the crude estimator of the ATE (i.e., $𝔼\left[N_{1}^{-1}\sum_{i}A_{i}Y_{i}-N_{0}^{-1}\sum_{i}(1-A_{i})Y_{i}\right]$) approximately equal to 0.05, 0.10, and 0.15, respectively.

Although g is selected through the target crude bias, larger values of g also make the treatment‐assignment and outcome mechanisms more strongly driven by covariates. As a result, larger values of g increase both confounding and practical overlap, and we refer to this factor as the scenario complexity. Under the 50% treatment‐prevalence calibration, the proportions of individuals with propensity scores between 5% and 95% are 99.5%, 95%, and 75% in the low‐, moderate‐, and high‐complexity scenarios, respectively. These values may differ in the 25% and 75% treatment‐prevalence scenarios.

The exact values of $a_{0}$, $b_{0}$, and g for each scenario are provided in the Supporting Information. For more details on how these parameters affect the distribution of data, an R Shiny app is available in the GitHub repository for this article [33].

To assess how weighting methods perform with smaller sample sizes, we created scenarios with 250, 500, 1000, or 2000 observations. In total, for the main simulation, there are 36 scenarios defined by sample size (250, 500, 1000, or 2000), proportion of treated (25%, 50%, or 75%), and level of complexity (low, moderate, or high). and treatment effect (0, 0.05, or 0.10). Sensitivity analysis repeat these scenarios under two non‐null constant treatment effects, yielding 108 simulated settings in total.

### 3.2 Implementation of Weighting Methods and Estimators

This section provides implementation details for the weighting methods described in Section 2.4 and the estimators introduced in Section 2.3, including the practical choices required to apply them and the software used. All the code for this study is available at this article's GitHub repository [33].

#### 3.2.1 Estimators

We implemented the WLS estimator in base R. For the DR estimator, we first fit outcome models within each treatment arm. Because the outcome is binary in our data‐generating mechanism, we used logistic regression including all covariates, with no variable selection or feature engineering. Confidence intervals were derived using method‐specific variance procedures described below. In all cases, the same set of weighting methods was combined with both the WLS and DR estimators for the ATE and ATT.

#### 3.2.2 IPTW

We estimated propensity scores via logistic regression including all covariates (no variable selection or feature engineering) and implemented the weighting in base R. Wald‐type confidence intervals were constructed using robust sandwich standard errors, implemented in base R. The variance estimator was based on a stacked M‐estimation system including the estimating equation for the target estimand and the logistic propensity‐score equations; for the DR estimator, the outcome‐regression score equations were also included.

#### 3.2.3 CBPS Just‐Identified

We implemented CBPS‐JI with the R package WeightIt [34], using method=“cbps” and over=FALSE to obtain the weights for both the ATE and ATT. In line with the original paper [12], we retained the default logistic propensity‐score specification and the default first‐moment balancing specification. Confidence intervals were derived in base R via stacked M‐estimation. For the WLS estimator, we stacked the estimating equation for the target estimand with the CBPS estimating equations. For the DR estimator, we additionally stacked the score equations of the treated and control outcome regressions. The resulting variance estimator was sandwich‐based and accounted for uncertainty due to CBPS‐JI propensity‐score estimation.

#### 3.2.4 CBPS Over‐Identified

We implemented CBPS‐OI with the R package WeightIt [34], using method=“cbps” and over=TRUE to obtain the weights for both the ATE and ATT. As for CBPS‐JI, we used the default logistic propensity‐score specification. We chose the continuous‐updating GMM estimator (twostep=FALSE), which is the version favored in the original article [12]. Wald‐type confidence intervals were derived in base R using robust sandwich standard errors conditional on the estimated CBPS‐OI weights. For both the WLS and DR estimators, the variation induced by the estimated weights was ignored; for the DR estimator, the score equations of the treated and control outcome regressions were still included. Accounting analytically for this source of uncertainty is possible, but over‐identified CBPS is defined through a continuous‐updating GMM optimization problem rather than a just‐identified system of estimating equations, so the direct stacked M‐estimation approach used for CBPS‐JI does not apply directly. Since no off‐the‐shelf implementation, either in the R package CBPS [29] or in WeightIt, provides this derivation for the confidence intervals of our WLS and DR estimators, we did not pursue it here.

#### 3.2.5 CBPS‐TLF

We implemented the CBPS‐TLF method following [13] and the author's R source package covalign [35]. In line with the practical guidance for low‐dimensional covariates, we modeled the propensity score using a Laplacian kernel and selected the regularization parameter λ by cross‐validation (section 5.6), minimizing the average norm of the tailored‐loss gradient on validation folds. The author recommends in [13, section 7] to use universal kernels, particularly the Laplacian, and encourages trying multiple kernels or bandwidths as a sensitivity analysis; no bandwidth‐selection rule is prescribed. Accordingly, we fixed the kernel family to Laplacian and set its bandwidth using the “median heuristic” (or its inverse, depending on parameterization), a standard convention in kernel methods [36, 37, 38, 39]. To reduce computation, we pre‐tuned λ per scenario and estimand via a grid over 50 replicated datasets, choosing the value that minimized the average imbalance proxy advocated in [13, section 5.6].

The author recommends an honest, design‐based CI that starts from a Wald interval and then adds a “worst‐case bias” margin based on a smoothness assumption for the outcome in a specified RKHS. In practice, this requires (i) choosing that function space and an upper bound on the outcome's complexity, which cannot be verified from the data and for which the paper provides limited implementation guidance in low or moderate dimensions; (ii) accepting additional restrictions (for non‐ATT estimands, a constant treatment effect); and (iii) tolerating intervals that can be overly conservative and therefore uninformative. For these reasons, we did not adopt this CI and instead used the same approach as for IPTW: robust (sandwich) standard errors obtained from a stacked M‐estimation system that includes the propensity‐score model but uncertainty due to kernel choice, bandwidth selection, and λ selection was not propagated.

#### 3.2.6 EB

Energy balancing requires no user‐specified tuning. We computed EB weights using the R package WeightIt with method=“energy” [34]. Although the original proposal recommends bootstrap‐based confidence intervals [14], full resampling was computationally prohibitive for our simulation grid. Instead, Wald‐type confidence intervals were constructed using robust sandwich standard errors conditional on the estimated EB weights; for the DR estimator, the outcome‐regression score equations were also included. Thus, the estimated variance did not account for uncertainty in the EB weight‐construction procedure. This was implemented in base R.

#### 3.2.7 KOM

KOM requires choosing, for each treatment arm a∈{0,1}, a kernel ${𝒦}_{a}$ and a bias‐variance penalty $\lambda_{a}$. Once the kernels were chosen, we tuned the kernel hyperparameters following the guidance in [16, section 3.6], within each arm, by maximizing the Gaussian process (GP) marginal likelihood for the outcome and set $\lambda_{a}$ to the noise‐to‐signal ratio. We used a Gaussian kernel for both arms. While [16, section 3.6] suggests a polynomial Mahalanobis kernel as a general default and lists Gaussian/Matérn as alternatives, we adopted a $C_{0}$‐universal kernel in light of broader recommendations for KOM in [15, section 4.7]. Moreover, the polynomial Mahalanobis kernel requires selecting an integer degree d (among other hyperparameters), which makes automated tuning less convenient than for the Gaussian kernel, whose primary length scale is a continuous parameter optimized by marginal likelihood.

To the best of our knowledge, there is no dedicated R package on CRAN implementing KOM. We therefore relied on the author's reference R code from a GitHub repository developed for a related article [40], with the following modifications: (i) we modified the computation of λ to match the recommendation of [16, section 3.6]; (ii) we replaced the quadratic‐programming (QP) solver Gurobi [41] with OSQP [42], an open‐source solver suited to large convex QPs; and (iii) we added analytic derivatives of the GP marginal likelihood to accelerate hyperparameter selection. We followed this strategy (see Section 2.4.5) and implemented the procedure in base R. Wald‐type confidence intervals were constructed using robust sandwich standard errors conditional on the estimated KOM weights; for the DR estimator, the outcome‐regression score equations were also included. Thus, the estimated variance did not account for uncertainty in the KOM weight‐construction procedure, kernel choice, or regularization‐parameter selection.

### 3.3 Performance Metrics

We compared estimators using Monte Carlo performance metrics that summarize both point‐estimation accuracy and confidence interval behavior. Let θ denote the true treatment effect, $\hat{\theta }$ an estimator of θ, and ${\hat{\theta }}_{i}$ the treatment‐effect estimate from replication i (i=1,…,R). For point‐estimation performance, we considered root mean squared error (RMSE), mean absolute error (MAE), empirical bias, and empirical variance:

$$\begin{array}{ll} \text{Bias}(\hat{\theta }) & =\frac{1}{R}\overset{R}{\underset{i=1}{\sum }}{\hat{\theta }}_{i}-\theta ,\;\text{MAE}(\hat{\theta })=R^{-1}\overset{R}{\underset{i=1}{\sum }}\left|{\hat{\theta }}_{i}-\theta \right|,\\ \text{RMSE}(\hat{\theta }) & =\sqrt{R^{-1}\overset{R}{\underset{i=1}{\sum }}{\left({\hat{\theta }}_{i}-\theta \right)}^{2}},\\ \mathrm{Var}(\hat{\theta }) & ={\left(R-1\right)}^{-1}\overset{R}{\underset{i=1}{\sum }}{\left({\hat{\theta }}_{i}-R^{-1}\overset{R}{\underset{j=1}{\sum }}{\hat{\theta }}_{j}\right)}^{2}. \end{array}$$

For confidence interval behavior, we computed 95% confidence interval coverage and the variance ratio, since coverage can be affected by both point‐estimate bias and miscalibrated variance estimation. Let $V_{i}$ denote the variance of ${\hat{\theta }}_{i}$ estimated by the corresponding method in replication i. The variance ratio compares the average method‐estimated variance with the empirical Monte Carlo variance of the point estimates. Then

$$\begin{array}{ll} \text{Coverage}(\hat{\theta }) & =R^{-1}\overset{R}{\underset{i=1}{\sum }}1\left(\theta \in {\text{CI}}_{95\%}({\hat{\theta }}_{i})\right),\\ \text{VR}(\hat{\theta }) & =\frac{R^{-1}\sum_{i=1}^{R}V_{i}}{\mathrm{Var}(\hat{\theta })}. \end{array}$$

The variance ratio was used to help interpret confidence interval coverage. Values below 1 suggest that the variance estimator underestimates the empirical Monte Carlo variance of the point estimates, whereas values above 1 suggest variance overestimation. Together, empirical bias, confidence interval coverage, and the variance ratio provide complementary information for describing the performance of each confidence interval procedure and identifying possible modes of failure, such as biased point estimates, variance underestimation, or overly conservative variance estimation.

For each performance metric, we computed a Monte Carlo standard error (MCSE) to quantify the simulation uncertainty due to using a finite number of replications. The MCSE is the estimated standard error of the Monte Carlo estimate of a performance metric, not the standard error of the treatment‐effect estimator itself. MCSEs were computed from first‐order approximations for each metric, in line with standard approaches for assessing Monte Carlo error in simulation studies [43, 44]. MCSEs were reported for bias, empirical variance, RMSE, MAE, coverage, and variance ratio in the Supporting Information. When missing values occurred, the corresponding replications were excluded from the computation of the affected metric; methods with missing values were reported together with the proportion of replications excluded.

## 4 Results

In this section, we present the main simulation results. All simulations were conducted in R (version 4.2.1; [45]). For each scenario, we generated 5000 datasets and applied IPTW, CBPS‐JI, CBPS‐OI, CBPS‐TLF, EB, and KOM. For each weighting method, estimand, and estimator, the 5000 treatment‐effect estimates were used to compute the performance metrics described in Section 3.3. The patterns reported below were consistent across the constant treatment effects evaluated in the sensitivity analysis, corresponding to ATE values of 0, 0.05, and 0.10. Detailed numerical results, including Monte Carlo standard errors, are provided in the Supporting Information. The full set of metrics can also be explored using the R Shiny application available from the article's GitHub repository [33].

We first report point‐estimation performance using RMSE as the main summary metric. We then briefly use empirical bias and empirical variance to explain the main RMSE patterns. Finally, we report confidence‐interval coverage and interpret it together with the variance ratio.

### 4.1 Point‐Estimation Performance

**FIGURE 1:**
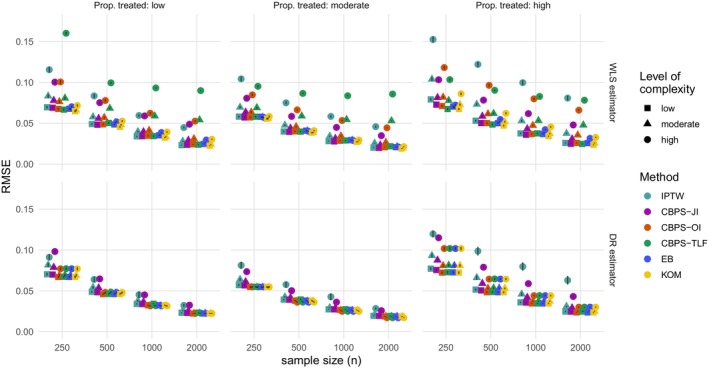
Root mean squared error (RMSE) of ATE estimates across simulation scenarios. Results are stratified by treatment prevalence (columns), estimator (rows), sample size (x‐axis), complexity level (point shape), and weighting method (color). Vertical segments represent 95% Monte Carlo confidence intervals for the estimated RMSE.

Figure 1 summarizes ATE point‐estimation performance. For a fixed treatment prevalence, sample size, and estimator, methods were nearly indistinguishable when complexity was low. Differences between methods appeared mainly as complexity increased. Overall, RMSE decreased with sample size and was lowest when treatment prevalence was moderate, but the impact of complexity was clearly method‐specific. Propensity‐score‐based methods were more sensitive to complexity than EB and KOM, which showed comparatively stable RMSE across scenarios.

Under WLS, EB and KOM had the lowest overall RMSE and behaved very similarly, with differences generally below 0.01 except in a few small‐sample scenarios with high treatment prevalence. Among IPTW, CBPS‐JI, and CBPS‐OI, CBPS‐JI generally had the lowest RMSE, followed by CBPS‐OI and then IPTW. CBPS‐TLF had less favorable performance under WLS: although it did not always have the largest RMSE in small samples, its RMSE plateaued as sample size increased, around 0.05 under moderate complexity and 0.08 under high complexity. As a result, CBPS‐TLF remained among the weakest methods for ATE estimation under WLS in moderate‐ and high‐complexity scenarios.

Under DR, differences between methods were smaller. CBPS‐OI, CBPS‐TLF, EB, and KOM had similar and low RMSE in most scenarios. IPTW remained the most sensitive to complexity, while CBPS‐JI showed intermediate behavior, with greater sensitivity to complexity than CBPS‐OI, CBPS‐TLF, EB, and KOM. Thus, the DR estimator reduced, but did not completely remove, the differences between weighting methods.

**FIGURE 2:**
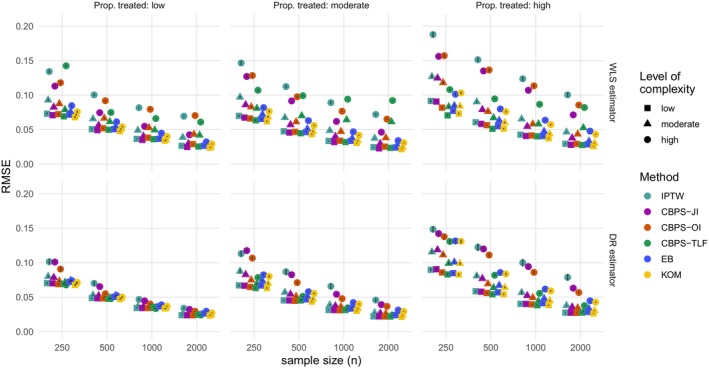
Root mean squared error (RMSE) of ATT estimates across simulation scenarios. Results are stratified by treatment prevalence (columns), estimator (rows), sample size (x‐axis), complexity level (point shape), and weighting method (color). Vertical segments represent 95% Monte Carlo confidence intervals for the estimated RMSE.

Most observations made for the ATE also held for the ATT Figure 2; here we only highlight the main differences. RMSE was generally higher for the ATT than for the ATE, and the prevalence pattern differed: ATT performance was more favorable when the treated proportion was low. Under WLS, CBPS‐TLF was less clearly separated from other propensity‐score methods in some high‐prevalence settings, but it still displayed the same plateau with increasing sample size. Under DR, EB, KOM, and CBPS‐TLF were again comparatively stable across complexity levels, with CBPS‐OI close to this group, while IPTW and CBPS‐JI remained more affected by complexity.

### 4.2 Bias and Empirical Variance

Bias and empirical‐variance results (figures and tables in Supporting Information) help explain the RMSE patterns. Bias generally increased with complexity and decreased with sample size, particularly under WLS. Empirical variance decreased with sample size; for the ATE, it was lowest under moderate treatment prevalence, whereas for the ATT it was lowest when treatment prevalence was low. These trends were more pronounced for propensity‐score‐based methods than for EB and KOM.

CBPS‐TLF deviated from this general pattern. Its empirical variance was very low across settings, but under WLS its bias did not vanish as sample size increased. For the ATE, the CBPS‐TLF bias plateaued at approximately 0.04 under moderate complexity and 0.08 under high complexity. This persistent bias explains the RMSE plateau observed for CBPS‐TLF. In contrast, the DR estimator strongly reduced bias for all methods, with bias approaching zero as sample size increased.

### 4.3 Confidence Interval Coverage and Variance Ratio

**FIGURE 3:**
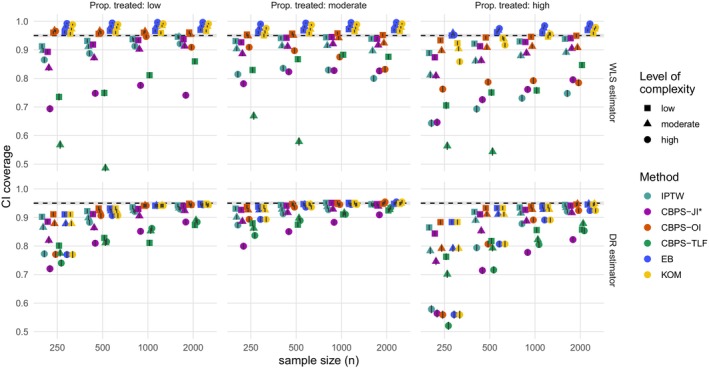
Empirical coverage of the 95% confidence interval for the ATE estimate across simulation scenarios. Results are stratified by treatment prevalence (columns), estimator (rows), sample size (x‐axis), complexity level (point shape), and weighting method (color). Vertical segments represent 95% Monte Carlo confidence intervals for the estimated coverage. The black dashed line is the nominal coverage level. The shaded reference band indicates the range compatible with nominal 95% coverage up to Monte Carlo error, based on 5000 simulation replicates, corresponding to approximately 94.4% to 95.6%.

**FIGURE 4:**
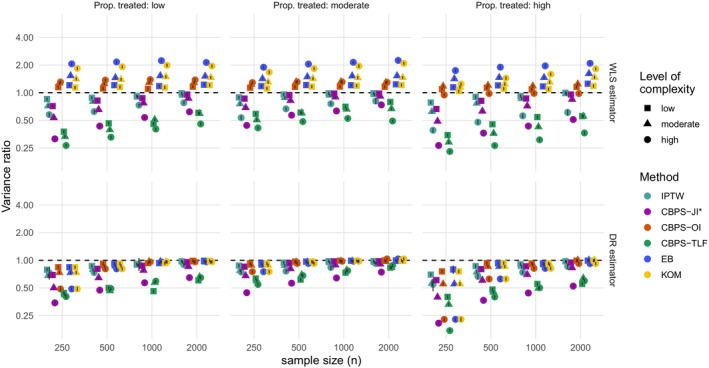
Variance ratio for the ATE estimates across simulation scenarios. Results are stratified by treatment prevalence (columns), estimator (rows), sample size (x‐axis), complexity level (point shape), and weighting method (color). Values below 1 indicate that the average estimated variance is smaller than the empirical Monte Carlo variance of the point estimates, whereas values above 1 indicate variance overestimation. Vertical segments represent 95% Monte Carlo confidence intervals for the estimated variance ratio.

Figures 3 and 4 summarize ATE confidence‐interval performance. Under WLS, EB and KOM tended to over‐cover, whereas propensity‐score‐based methods tended to under‐cover. IPTW was close to nominal coverage in low‐complexity scenarios, but coverage decreased as complexity increased, especially when treatment prevalence was high. CBPS‐OI had better coverage than IPTW and CBPS‐JI. Between IPTW and CBPS‐JI, the ordering depended on treatment prevalence: CBPS‐JI performed better when treatment prevalence was far from 50%, whereas IPTW performed better when treatment prevalence was moderate. CBPS‐TLF had the weakest WLS coverage overall. Its coverage remained below nominal even under low complexity, plateauing around 85% to 90%, and dropped to around 25% under moderate complexity and near 0% under high complexity.

The variance ratio helps explain these coverage patterns. Under WLS for the ATE, CBPS‐OI, EB, and KOM had variance ratios above 1, indicating variance overestimation. This overestimation was mild for CBPS‐OI, generally not exceeding about 1.2, but was more pronounced for EB and KOM where ratios were often around 1.5 and 1.8 under moderate and high complexity respectively. In contrast, IPTW, CBPS‐JI, and CBPS‐TLF tended to underestimate variance, although their variance ratios moved closer to 1 as sample size increased. This separation is consistent with differences in the implemented inference procedures: IPTW, CBPS‐JI, and CBPS‐TLF account for weight‐estimation uncertainty through the implemented stacked procedure, whereas CBPS‐OI, EB, and KOM are treated more conditionally on the constructed weights.

Under DR, coverage patterns were more homogeneous across methods. Most methods under‐covered in small samples but moved toward nominal coverage as sample size increased, with smaller sensitivity to complexity than under WLS. CBPS‐JI retained some sensitivity to complexity, and CBPS‐TLF improved relative to WLS but converged more slowly toward nominal coverage. For all methods, coverage was less favorable when treatment prevalence was far from 50%. Variance ratios under DR followed the same broad pattern: they were often below 1 in small samples and moved toward 1 as sample size increased, with higher complexity generally worsening variance underestimation.

ATT coverage and variance‐ratio results are reported in the Supporting Information and can be explored in the Shiny application. Most ATE conclusions remained true for the ATT, with two differences. First, under WLS, CBPS‐JI tended to over‐cover when treatment prevalence was low, was close to nominal coverage when treatment prevalence was moderate, and slightly under‐covered when treatment prevalence was high; its sensitivity to complexity was lower than that of CBPS‐OI. Second, under DR, the methods behaved similarly: coverage was close to nominal when treatment prevalence was low, whereas moderate and high treatment prevalence produced small‐sample undercoverage that decreased as sample size increased. In some ATT settings, especially with small sample size, high complexity, or high treatment prevalence, the CBPS‐JI variance‐estimation procedure was unstable. In those settings, the CBPS‐JI coverage and variance‐ratio metrics should not be overinterpreted. Outside these CBPS‐JI ATT settings, point‐estimate and standard‐error missingness was negligible.

## 5 Application to PROBITsim Data

In this section, we assess the methods on probitsim [46]. probitsim is a synthetic observational cohort, calibrated to summary characteristics of the PROBIT randomized trial, but not a direct replication. It generates individual mother‐infant pairs and focuses on infant weight at 3 months, measured in grams, as the primary outcome. The data include baseline covariates commonly available in clinical records (maternal age; region: urban/rural, west/east; education: low/intermediate/high; allergy history; smoking during pregnancy) and birth‐related variables (infant sex, birthweight, caesarean section). The sample size is set to n=17044, as in PROBIT, and the data‐generating mechanism induces realistic confounding and selected interactions (e.g., between breastfeeding duration and education, smoking, or birthweight). probitsim was created as a practice‐oriented benchmark for causal‐inference methods: by calibrating to real clinical summaries (covariate mix, prevalences, correlations, and overlap) while preserving a known data‐generating mechanism, it delivers trial‐like realism with a known ground truth for the causal estimands. Treatment assignment is intentionally nonrandom to induce realistic confounding and overlap patterns. We therefore use probitsim as a complementary empirical illustration.

The intervention is represented as a chain of four linked point exposures with the temporal order:

i. $A_{1}$, offer of a breastfeeding encouragement program (BEP);
ii. $A_{2}$, BEP uptake;
iii. $A_{3}$, breastfeeding initiation;
iv. $A_{4}$, breastfeeding maintained for 3 months.

Importantly, BEP uptake $A_{2}$ is defined as conditional on being offered the BEP $A_{1}$: a mother can enroll only if an offer was made. For each unit, probitsim generates potential outcomes under alternative exposure strategies, making the true treatment effects known and allowing us to evaluate estimators against ground truth. For BEP uptake ($A_{2}$), the focus of our analysis, the true treatment effects are ATE=165 g and ATT=153 g for weight at 3 months. Code and further details are available from the authors' materials.

We applied the same weighting methods as in the simulation study. Because infant weight is continuous, linear regression was used for the outcome models in the DR estimator. For IPTW, the propensity score model followed the authors' specification for exposure $A_{2}$. Implementations of CBPS‐JI, CBPS‐OI, CBPS‐TLF, EB, and KOM followed the strategies detailed in Section 3.2 without further modification. Code for the analysis is available in the GitHub repository for this article [33].

The estimates and confidence intervals in Table 1 show that the DR estimates are very similar across methods and close to the true effects for both the ATE and the ATT. For the ATE, all DR estimates are between 164 and 165 g, with similar interval widths. This suggests that, in this application, the outcome‐regression component reduces sensitivity to the choice of weighting method. Under WLS, method‐specific differences are more visible. IPTW, CBPS‐JI, CBPS‐OI, EB, and KOM all give ATE estimates whose confidence intervals include the true value of 165 g. CBPS‐TLF is the main exception, with a WLS ATE estimate of 188 g and a 95% CI of [170,207], which does not include the true value. For the ATT, all confidence intervals include the true value of 153 g. IPTW, CBPS‐JI, CBPS‐OI, EB, and KOM give WLS ATT estimates clustered around 148–149 g, whereas CBPS‐TLF gives a higher estimate of 171 g, with the lower confidence bound equal to 153 g. Overall, these results are consistent with the main simulation findings: DR estimation is less sensitive to the weighting method, EB and KOM behave stably under WLS, standard CBPS behaves similarly to IPTW in this application, and CBPS‐TLF‐WLS deviates more from the true effect than the other WLS analyses.

**TABLE 1:** ATE and ATT estimates (with 95% CIs) for the effect of BEP participation ($A_{2}$) on infant weight (g) at 3 months in probitsim. True effects: ATE = 165 g, ATT = 153 g.

	ATE		ATT	
Method	WLS	DR	WLS	DR
IPTW	165[146,184]	164[145,183]	148[129,167]	149[130,167]
CBPS‐JI	164[145,183]	164[145,183]	149[130,167]	149[130,167]
CBPS‐OI	165[146,184]	165[146,184]	148[130,167]	149[130,168]
CBPS‐TLF	188[170,207]	165[146,184]	171[153,190]	148[130,167]
EB	158[138,178]	165[146,184]	148[128,167]	148[128,167]
KOM	167[148,186]	165[146,184]	149[131,168]	149[130,167]

## 6 Discussion

These results are best interpreted as practical guidance on the strengths, limitations, and failure modes of the evaluated weighting methods, rather than as a definitive ranking. Overall, IPTW and CBPS‐TLF were more sensitive to scenario complexity, with larger deterioration under WLS in moderate‐ and high‐complexity settings. CBPS‐JI and CBPS‐OI showed behavior close to IPTW but with less sensitivity to the level of complexity when treatment prevalence was high. EB and KOM had more stable point‐estimation performance across many scenarios and showed very similar RMSE patterns. Under DR estimation, differences between weighting methods were generally smaller, although confidence‐interval performance remained more heterogeneous.

The favorable performance of the DR estimator should nevertheless be interpreted within the scope of the simulated data‐generating mechanism. In this study, all confounders were included in the fitted nuisance models, treatment effects were homogeneous on the additive scale, and treatment‐covariate interactions were absent. The simulations therefore do not imply that DR estimation would be equally stable under stronger nuisance‐model misspecification or under more severe extrapolation. In particular, when empirical overlap is limited, the outcome‐regression component may rely on predictions in regions with limited data support, and the credibility of this extrapolation depends on the correctness and flexibility of the outcome model.

Confidence‐interval performance should also be interpreted as the performance of a weighting‐method and inference‐procedure combination, rather than as a property of the weighting method alone. Standard stacked M‐estimation works most naturally for smooth finite‐dimensional nuisance models, such as logistic propensity‐score estimation, and was used here for IPTW, CBPS‐JI, and CBPS‐TLF. For CBPS‐OI, EB, and KOM, the sandwich procedures used in this study were conditional on the constructed weights and therefore did not fully account for uncertainty in the weight‐construction step. For CBPS‐TLF, the implemented variance procedure was also conditional on the selected tuning parameters and did not account for kernel bandwidth or lambda selection. This distinction may partly explain why some procedures that condition on the weights tended to overestimate variance, although this pattern may also reflect properties of the corresponding weighting methods themselves. Also, it is worth reminding that the double robustness of the point estimator should not be confused with the automatic validity of the corresponding confidence interval.

### 6.1 On the Relation Between EB and KOM

Although EB is presented as a distance‐based method, it can be seen as an instance of KOM. Indeed, EB chooses weights by minimizing a weighted empirical energy distance between covariate distributions, where the underlying dissimilarity is the squared Euclidean norm ${\left\|\cdot \right\|}_{2}^{2}$ on ${\mathbb{R}}^{p}$ [14]. This energy distance is equal, up to a constant factor, to the squared maximum mean discrepancy computed in a RKHS induced by the distance‐induced kernel [47] $k(x,x^{\prime })=\frac{1}{2}\left({\left\|x-x_{0}\right\|}_{2}^{2}+{\left\|x^{\prime }-x_{0}\right\|}_{2}^{2}-{\left\|x-x^{\prime }\right\|}_{2}^{2}\right)$, for any fixed anchor $x_{0}$. The resulting maximum mean discrepancy between distributions does not depend on the choice of $x_{0}$, and $ℰ(F,G)=2\;{\text{MMD}}_{k}^{2}(F,G)$ [47, Thm. 22]. Consequently, EB is an instance of KOM with kernel k and a variance‐regularization parameter set to λ=0.

### 6.2 Code Accessibility

Accessible, author‐supported implementations are crucial for applied use and reproducible comparisons. IPTW and standard CBPS were the most accessible methods in this study: IPTW can be implemented directly from fitted propensity scores, and CBPS‐JI and CBPS‐OI can be obtained from established R implementations such as WeightIt. EB is now also available in WeightIt [34], although clearly linked author‐supported code was not available at the time this comparison was initiated. For KOM, author reference code facilitated implementation, but some reconciliation between the paper and code was required. For CBPS‐TLF, an R source package, covalign [35], existed but was not referenced in the article and the recommended routine was not exported, forcing users to inspect source internals. Such barriers raise the risk of user‐side re‐implementation, bugs, or omission of impactful details, such as Gram‐matrix diagonalization in CBPS‐TLF or SVD decomposition in CBPS‐JI and CBPS‐OI.

### 6.3 Weight Post‐Processing

We chose not to apply weight post‐processing, such as truncation [8], trimming [9], or stabilization [10], to keep IPTW as a simple and transparent reference method. This choice should not be interpreted as presenting the strongest possible IPTW implementation. IPTW's performance has been extensively studied, and careful propensity‐score modeling combined with diagnostics and post‐processing can work well in practice. Our goal was instead to compare the evaluated methods under a common implementation strategy, without introducing an additional layer of method‐specific choices. For applied work, we refer readers to practical guidance on diagnostics and post‐processing [11].

### 6.4 Possible Explanations for CBPS‐TLF Performance

CBPS‐TLF performed poorly under the tuning strategy used here, especially under WLS. We assessed it using author‐guided and standard choices: the recommended kernel family, a heuristic bandwidth, and a regularization parameter pre‐tuned using the proposed balance criterion. This particular recipe may be mismatched to our design. CBPS‐TLF may be sensitive to the kernel family, bandwidth, and regularization parameter, and other tuning choices discussed by the author could plausibly perform better. However, a full tuning‐sensitivity analysis would require joint exploration of several hyperparameters across many scenarios and replications, which was outside the scope of this study. Developing and validating a practical finite‐sample tuning strategy for CBPS‐TLF is therefore a separate methodological problem. Better finite‐sample guidance for hyperparameter selection is needed before routine applied use in settings like ours.

### 6.5 Scope of Estimands, Outcomes, and Treatment Effects

This study focused on additive marginal effects, namely the ATE and ATT. For binary outcomes, these estimands correspond to risk differences. Risk ratios and odds ratios are important in applied work, but they were outside the scope of this simulation study. This focus is consistent with the compared methods and source‐paper estimators, which are primarily formulated for weighted mean contrasts or additive effects. The conclusions should therefore not be automatically generalized to relative effect measures. Extending the comparison to risk ratios or odds ratios would require additional estimator definitions and confidence‐interval procedures, especially for DR estimators.

The main simulation study was conducted under a null treatment effect, with constant treatment effects of 0.05 and 0.10 considered as sensitivity analyses. Results from the main null‐effect scenarios should therefore be interpreted alongside the non‐null constant‐effect sensitivity analyses, and may not fully generalize to other effect sizes, relative effect measures, or heterogeneous treatment effects. Choosing additional non‐null effect sizes would require further design decisions, especially relative to baseline risk and confounding bias. In addition, the data‐generating mechanism did not include treatment‐covariate interactions, so treatment effects were homogeneous on the additive scale. This makes the outcome‐regression component of the DR estimator less challenging than in settings with heterogeneous treatment effects.

### 6.6 Missing Covariate Data and Treatment‐Type Restrictions

This study also assumed complete covariate data and a binary treatment. These restrictions are important in applied settings. In the source papers reviewed here, missing baseline covariates were generally not treated as a core methodological issue for the compared weighting methods [12, 13, 14, 15, 16]. Later work in the broader propensity‐score literature provides guidance for combining IPTW with multiple imputation, but comparable method‐specific guidance was limited for standard CBPS, CBPS‐TLF, Energy balancing, and KOM [48]. Energy balancing used imputation operationally in applied examples, but did not develop a general missing‐covariate procedure [14]. Extensions beyond binary treatment were more uneven: IPTW has well‐established links to multilevel, continuous, and time‐varying treatment settings [49, 50, 51]; standard CBPS includes multivalued‐treatment extensions and later continuous‐treatment developments [12, 52]; Energy balancing explicitly allows multi‐category treatments [14]; CBPS‐TLF was developed in the binary‐treatment setting, although the source paper briefly notes that extension to multiple or continuous treatments should be possible [13]; and KOM was also binary‐focused in the source papers reviewed here, with continuous‐treatment weighting addressed in later related kernel‐weighting work [53].

### 6.7 Practical Recommendation

When empirical overlap is strong and a reasonably specified propensity‐score model is available, IPTW is a natural and effective starting point and is well covered by existing guidance [1, 2, 11]. Standard CBPS, including CBPS‐JI and CBPS‐OI, provides a useful alternative within the propensity‐score weighting family because it estimates the propensity score while also targeting covariate balance [12]. In our simulations, CBPS‐JI often improved point‐estimation performance relative to IPTW under WLS, whereas CBPS‐OI was useful for understanding the behavior of over‐identified CBPS and generally showed more favorable ATE coverage than IPTW and CBPS‐JI under WLS. These patterns should still be interpreted together with the implemented confidence‐interval procedures.

When empirical overlap is limited or propensity‐score model specification is uncertain, IPTW can become fragile, for example through extreme weights, and often requires expert interventions such as trimming or alternative targets [9, 54, 55, 56]. In such settings, it can be useful to triangulate results using alternative weighting approaches and to examine the induced target population, weight behavior, overlap, and, when relevant, covariate balance. EB and KOM are two practical candidates for such sensitivity analyses. EB can be deployed with few tuning choices and is implemented in WeightIt [14, 34], whereas KOM offers an explicit bias‐variance trade‐off in a RKHS but requires selecting a kernel and a regularization parameter and is typically used via authors' research code [15, 16, 40]. Since EB relies on Euclidean distances and KOM may also rely on Euclidean distances through the kernel, for example when using a Gaussian kernel, both approaches can be sensitive to the scaling of covariates; standardization is therefore advisable before applying these methods [30]. Although CBPS‐TLF is a promising approach [13], our simulations indicate that the author‐guided tuning strategy used here did not yield strong finite‐sample performance for WLS treatment‐effect estimates. Until more practical finite‐sample tuning guidance is available, applied teams without specialized expertise may wish to prioritize IPTW, standard CBPS, EB, and KOM, while treating CBPS‐TLF results as more exploratory.

## Author Contributions

All authors were involved in the study concept and design, the analysis and interpretation of the data and, the drafting of the manuscript. E.P. did the code implementation, the figures and the tables.

## Funding

This work was supported by the Agence Nationale de la Recherche (Grant Nos. ANR‐22‐CPJ1‐0047‐01, ANR‐23‐IACL‐0008, and ANR‐18‐CE36‐0010‐01).

## Disclosure

Etienne Peyrot acknowledges support from the Université Paris Cité. Francois Petit acknowledges support from the French Agence Nationale de la Recherche through the project reference ANR‐22‐CPJ1‐0047‐01. Raphaël Porcher acknowledges support from the French Agence Nationale de la Recherche as part of the “Investissements d'avenir” program, reference ANR‐23‐IACL‐0008 (PR[AI]RIE‐PSAI IA cluster). This work was partially funded by the Agence Nationale de la Recherche, under grant agreement no. ANR‐18‐CE36‐0010‐01.

## Conflicts of Interest

The authors declare no conflicts of interest.

## Supporting information




**Data S1:** Supporting Information.

## Data Availability

R code for data generation and analysis for the current study are available in this article GitHub repository [33]. Docker used to run the simulation available for download at https://cloud.sylabs.io/library/ep123456/collection/v7. The probitsim dataset [46] analysed during the current study is available in the GitHub repository https://github.com/IngWae/Formulating‐causal‐questions. The data that support the findings of this study are openly available in benchmark_balancing_methods at https://github.com/EtiennePeyrot.

## References

[1] P. R. Rosenbaum and D. B. Rubin , “The Central Role of the Propensity Score in Observational Studies for Causal Effects,” Biometrika 70, no. 1 (1983): 41–55.

[2] J. K. Lunceford and M. Davidian , “Stratification and Weighting via the Propensity Score in Estimation of Causal Treatment Effects: A Comparative Study,” Statistics in Medicine 23, no. 19 (2004): 2937–2960. 15351954 10.1002/sim.1903

[3] J. Hahn , “On the Role of the Propensity Score in Efficient Semiparametric Estimation of Average Treatment Effects,” Econometrica 66, no. 2 (1998): 315–331.

[4] J. M. Smit , J. H. Krijthe , K. WMR , et al., “Causal Inference Using Observational Intensive Care Unit Data: A Scoping Review and Recommendations for Future Practice,” NPJ Digital Medicine 6, no. 1 (2023): 221. 38012221 10.1038/s41746-023-00961-1 PMC10682453

[5] H. Zuo , L. Yu , S. M. Campbell , S. S. Yamamoto , and Y. Yuan , “The Implementation of Target Trial Emulation for Causal Inference: A Scoping Review,” Journal of Clinical Epidemiology 162 (2023): 29–37. 37562726 10.1016/j.jclinepi.2023.08.003

[6] P. R. Rosenbaum , “Model‐Based Direct Adjustment,” Journal of the American Statistical Association 82, no. 398 (1987): 387–394.

[7] D. G. Horvitz and D. J. Thompson , “A Generalization of Sampling Without Replacement From a Finite Universe,” Journal of the American Statistical Association 47, no. 260 (1952): 663–685.

[8] Y. Xiao , E. E. M. Moodie , and M. Abrahamowicz , “Comparison of Approaches to Weight Truncation for Marginal Structural Cox Models,” Epidemiological Methods 2, no. 1 (2013): 1–20.

[9] T. Stürmer , M. Webster‐Clark , J. L. Lund , et al., “Propensity Score Weighting and Trimming Strategies for Reducing Variance and Bias of Treatment Effect Estimates: A Simulation Study,” American Journal of Epidemiology 190, no. 8 (2021): 1659–1670. 33615349 10.1093/aje/kwab041 PMC8327194

[10] S. Xu , C. Ross , M. A. Raebel , S. Shetterly , C. Blanchette , and D. Smith , “Use of Stabilized Inverse Propensity Scores as Weights to Directly Estimate Relative Risk and Its Confidence Intervals,” Value in Health 13, no. 2 (2010): 273–277. 19912596 10.1111/j.1524-4733.2009.00671.x PMC4351790

[11] P. C. Austin and E. A. Stuart , “Moving Towards Best Practice When Using Inverse Probability of Treatment Weighting (IPTW) Using the Propensity Score to Estimate Causal Treatment Effects in Observational Studies,” Statistics in Medicine 34, no. 28 (2015): 3661–3679. 26238958 10.1002/sim.6607 PMC4626409

[12] K. Imai and M. Ratkovic , “Covariate Balancing Propensity Score,” Journal of the Royal Statistical Society. Series B, Statistical Methodology 76, no. 1 (2014): 243–263.

[13] Q. Zhao , “Covariate Balancing Propensity Score by Tailored Loss Functions,” Annals of Statistics 47, no. 2 (2019): 965–993.

[14] J. D. Huling and S. Mak , “Energy Balancing of Covariate Distributions,” Journal of Causal Inference 12, no. 1 (2024): 20220029.

[15] N. Kallus , “Generalized Optimal Matching Methods for Causal Inference,” Journal of Machine Learning Research 21, no. 62 (2020): 1–54.34305477

[16] N. Kallus and M. Santacatterina , “Optimal Estimation of Generalized Average Treatment Effects Using Kernel Optimal Matching,” arXiv Preprint arXiv:190804748, (2019).

[17] J. M. Franklin , J. A. Rassen , D. Ackermann , D. B. Bartels , and S. Schneeweiss , “Metrics for Covariate Balance in Cohort Studies of Causal Effects,” Statistics in Medicine 33, no. 10 (2013): 1685–1699. 24323618 10.1002/sim.6058

[18] J. M. Robins , A. Rotnitzky , and L. P. Zhao , “Estimation of Regression Coefficients When Some Regressors Are Not Always Observed,” Journal of the American Statistical Association 89, no. 427 (1994): 846–866.

[19] D. O. Scharfstein , A. Rotnitzky , and J. M. Robins , “Adjusting for Nonignorable Drop‐Out Using Semiparametric Nonresponse Models,” Journal of the American Statistical Association 94, no. 448 (1999): 1096–1120.

[20] A. Tsiatis , Semiparametric Theory and Missing Data, vol. 73 (Springer New York, 2006).

[21] M. J. van der Laan and D. Rubin , “Targeted Maximum Likelihood Learning,” International Journal of Biostatistics 2, no. 1 (2006): 1–40.

[22] D. B. Rubin , “Estimating Causal Effects of Treatments in Randomized and Nonrandomized Studies,” Journal of Educational Psychology 66, no. 5 (1974): 688–701.

[23] J. Neyman , “On the Application of Probability Theory to Agricultural Experiments. Essay on Principles. Section 9 (Translation Published in 1990),” Statistical Science 5 (1923): 472–480.

[24] J. Hájek , “Comment on “an Essay on the Logical Foundations of Survey Sampling, Part One” by D. Basu,” in Foundations of Statistical Inference, ed. V. P. Godambe and D. A. Sprott (Holt, Rinehart and Winston, 1971), 236–248.

[25] W. K. Newey and D. McFadden , “Chapter 36 Large Sample Estimation and Hypothesis Testing,” in Handbook of Econometrics, vol. 4 (Elsevier, 1994), 2111–2245.

[26] A. W. van der Vaart , Asymptotic Statistics. Cambridge Series in Statistical and Probabilistic Mathematics (Cambridge University Press, 1998).

[27] L. A. Stefanski and D. D. Boos , “The Calculus of M‐Estimation,” American Statistician 56, no. 1 (2002): 29–38.

[28] L. P. Hansen , “Large Sample Properties of Generalized Method of Moments Estimators,” Econometrica 50, no. 4 (1982): 1029–1054.

[29] C. Fong , M. Ratkovic , and K. Imai , “CBPS: Covariate Balancing Propensity Score,” Comprehensive R Archive Network, R package version 0.24, (2025), https://CRAN.R‐project.org/package=CBPS.

[30] G. J. Székely and M. L. Rizzo , “Energy Statistics: A Class of Statistics Based on Distances,” Journal of Statistical Planning and Inference 143, no. 8 (2013): 1249–1272.

[31] D. A. Freedman , “On the So‐Called “Huber Sandwich Estimator” and “Robust Standard Errors”,” American Statistician 60, no. 4 (2006): 299–302.

[32] J. D. Y. Kang and J. L. Schafer , “Demystifying Double Robustness: A Comparison of Alternative Strategies for Estimating a Population Mean From Incomplete Data,” Statistical Science 22, no. 4 (2007): 523–539. 10.1214/07-STS227 PMC2397555 18516239

[33] E. Peyrot , “Choosing Covariate Balancing Methods for Causal Inference: Practical Insights from a Simulation Study ‐ Analysis Code and Materials,” GitHub, (2025), https://github.com/EtiennePeyrot/benchmark_balancing_methods.10.1002/sim.7067242420803

[34] N. Greifer , “WeightIt: Weighting for Covariate Balance in Observational Studies,” R package version 0.13.1. Comprehensive R Archive Network, (2022), https://CRAN.R‐project.org/package=WeightIt.

[35] Q. Zhao , Covalign: R Source Code for Covariate Balancing by Tailored Loss Functions (CBSR/TLF) (University of Cambridge, 2019), https://www.statslab.cam.ac.uk/qz280/publication/balancing‐loss/.

[36] A. Gretton , K. M. Borgwardt , M. J. Rasch , B. Schölkopf , and A. Smola , “A Kernel Two‐Sample Test,” Journal of Machine Learning Research 13, no. 25 (2012): 723–773.

[37] D. Garreau , W. Jitkrittum , and M. Kanagawa , “Large Sample Analysis of the Median Heuristic,” arXiv Preprint arXiv:170707269, (2017).

[38] A. Ramdas , S. J. Reddi , B. Póczos , A. Singh , and L. Wasserman , “On the Decreasing Power of Kernel and Distance Based Nonparametric Hypothesis Tests in High Dimensions,” Proceedings of the AAAI Conference on Artificial Intelligence 29, no. 1 (2015): 3571–3577.

[39] A. Schrab , I. Kim , M. Albert , B. Laurent , B. Guedj , and A. Gretton , “MMD Aggregated Two‐Sample Test,” Journal of Machine Learning Research 24, no. 194 (2023): 1–81.

[40] N. Kallus , B. Pennicooke , and M. Santacatterina , “More Robust Estimation of Sample Average Treatment Effects Using Kernel Optimal Matching in an Observational Study of Spine Surgical Interventions,” arXiv Preprint arXiv:181104274, (2018), https://github.com/CausalML/KOM‐SATE. 10.1002/sim.8904 33665870

[41] Gurobi Optimization, LLC , “Gurobi Optimizer Reference Manual,” Gurobi Optimization, LLC, (2024), https://www.gurobi.com.

[42] B. Stellato , G. Banjac , P. Goulart , and S. Boyd , “OSQP: Quadratic Programming Solver Using the OSQP Library,” R package version 0.6.0.5. Comprehensive R Archive Network; (2021), https://CRAN.R‐project.org/package=osqp.

[43] I. R. White , “Simsum: Analyses of Simulation Studies Including Monte Carlo Error,” Stata Journal 10, no. 3 (2010): 369–385.

[44] E. Koehler , E. Brown , and S. J. P. A. Haneuse , “On the Assessment of Monte Carlo Error in Simulation‐Based Statistical Analyses,” American Statistician 63, no. 2 (2009): 155–162. 22544972 10.1198/tast.2009.0030 PMC3337209

[45] R Core Team , R: A Language and Environment for Statistical Computing (R Foundation for Statistical Computing, 2022), https://www.R‐project.org/.

[46] E. Goetghebeur , S. le Cessie , B. De Stavola , M. EEM , and I. Waernbaum , “Formulating Causal Questions and Principled Statistical Answers,” Statistics in Medicine 39, no. 30 (2020): 4922–4948. 32964526 10.1002/sim.8741 PMC7756489

[47] D. Sejdinovic , B. Sriperumbudur , A. Gretton , and K. Fukumizu , “Equivalence of Distance‐Based and RKHS‐Based Statistics in Hypothesis Testing,” Annals of Statistics 41, no. 5 (2013): 2263–2291.

[48] C. Leyrat , S. R. Seaman , I. R. White , et al., “Propensity Score Analysis With Partially Observed Covariates: How Should Multiple Imputation Be Used?,” Statistical Methods in Medical Research 28, no. 1 (2019): 3–19. 28573919 10.1177/0962280217713032 PMC6313366

[49] J. M. Robins , M. A. Hernán , and B. Brumback , “Marginal Structural Models and Causal Inference in Epidemiology,” Epidemiology 11, no. 5 (2000): 550–560. 10955408 10.1097/00001648-200009000-00011

[50] G. W. Imbens , “The Role of the Propensity Score in Estimating Dose‐Response Functions,” Biometrika 87, no. 3 (2000): 706–710.

[51] K. Hirano and G. W. Imbens , “The Propensity Score With Continuous Treatments,” in Applied Bayesian Modeling and Causal Inference From Incomplete‐Data Perspectives, ed. A. Gelman and X. L. Meng (John Wiley & Sons, Ltd., 2004), 73–84.

[52] C. Fong , C. Hazlett , and K. Imai , “Covariate Balancing Propensity Score for a Continuous Treatment: Application to the Efficacy of Political Advertisements,” Annals of Applied Statistics 12, no. 1 (2018): 156–177.

[53] N. Kallus and M. Santacatterina , “Kernel Optimal Orthogonality Weighting: A Balancing Approach to Estimating Effects of Continuous Treatments,” arXiv Preprint arXiv:191011972, (2019).

[54] R. K. Crump , V. J. Hotz , G. W. Imbens , and O. A. Mitnik , “Dealing With Limited Overlap in Estimation of Average Treatment Effects,” Biometrika 96, no. 1 (2009): 187–199.

[55] B. K. Lee , J. Lessler , and E. A. Stuart , “Weight Trimming and Propensity Score Weighting,” PLoS One 6, no. 3 (2011): e18174. 21483818 10.1371/journal.pone.0018174 PMC3069059

[56] F. Li and L. E. Thomas , “Addressing Extreme Propensity Scores via the Overlap Weights,” American Journal of Epidemiology 188, no. 1 (2018): 250–257. 10.1093/aje/kwy201 30189042

