# Supplementary material for: Choosing Covariate Balancing Methods for Causal Inference: Practical Insights From a Simulation Study
Source: Stat Med. 2026 Jul 8;45(15-17):e70672. doi: 10.1002/sim.70672 (PMC13346537; doi:10.1002/sim.70672)
Supplement: Supplementary file 1 — Data S1: Supporting Information. [file SIM-45-0-s001.zip › sim70672-sup-0001-Supinfo/supp_material.pdf]

Choosing Covariate Balancing Methods for Causal Inference: Practical Insights from a Simulation Study

Etienne Peyrot[1], Raphaël Porcher[1,2], and François Petit[1]

[1]Université Paris Cité and Université Sorbonne Paris Nord, Inserm, INRAE, Center for Research in Epidemiology and StatisticS (CRESS), F-75004 Paris, France

[2]Centre d'Épidémiologie Clinique, Assistance Publique-Hôpitaux de Paris, Hôtel-Dieu, Paris, France

1 Supplementary material

1.1 Data generative mechanism

The values of the data-generative mechanism's hyperparameters a_0, b_0, and g for each scenario are reported below. The parameter g depends only on the level of complexity, b_0 depends on the level of complexity and the proportion of treated units, and a_0 depends on the level of complexity, the proportion of treated units, and the treatment-effect level.

Table 1: g by level of complexity.

Complexity	g
low	0.70
moderate	1.20
high	2.15

Table 2: b_0 by proportion of treated and level of complexity.

Complexity	Prop. treated		
	low	moderate	high
low	-1.48	-0.19	1.07
moderate	-1.87	-0.32	1.20
high	-2.73	-0.54	1.59

1.2 Supplementary figures

In this section, we present the figures summarizing the results of our main simulations. For each of the 36 scenarios, we show the bias, variance, RMSE, MAE, and coverage of the 95% confidence

Table 3: a_0 by treatment-effect level, proportion of treated, and level of complexity.

Effect level	Complexity	Prop. treated		
		low	moderate	high
none	low	-1.299	-1.300	-1.296
	moderate	-1.645	-1.639	-1.649
	high	-2.485	-2.487	-2.461
small	low	-1.382	-1.479	-1.558
	moderate	-1.767	-1.877	-1.976
	high	-2.653	-2.814	-2.975
high	low	-1.469	-1.650	-1.856
	moderate	-1.878	-2.104	-2.375
	high	-2.814	-3.155	-3.551

intervals for each combination method-estimator-estimand.

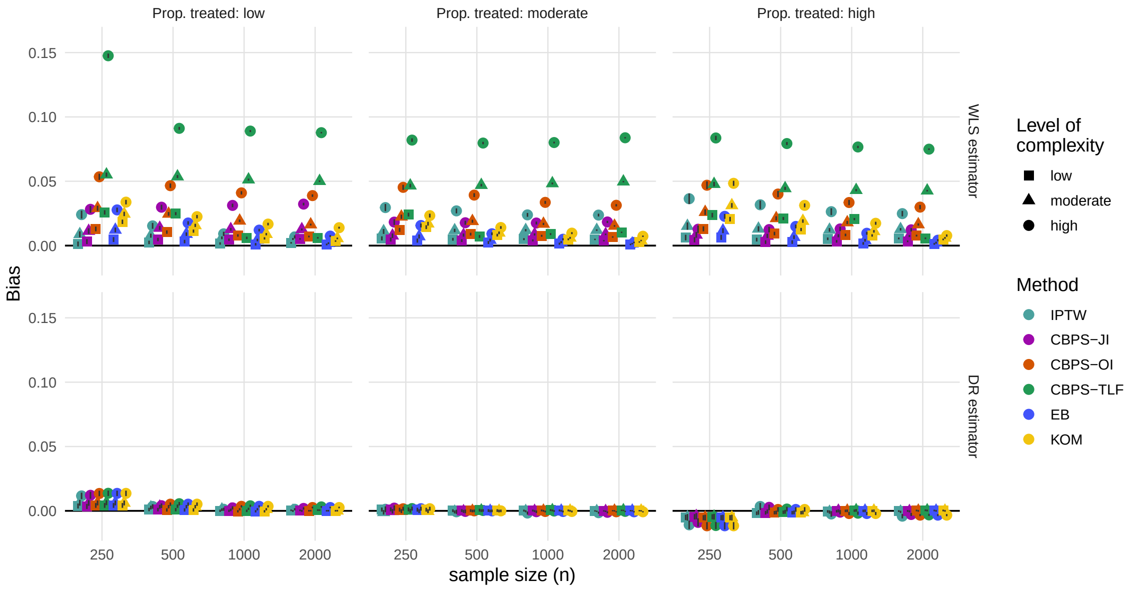

Figure 1: Bias of ATE estimates across simulation scenarios. Results are stratified by treatment prevalence (columns), estimator (rows), sample size (x-axis), complexity level (point shape), and weighting method (color). Vertical segments represent 95% Monte Carlo confidence intervals for the estimated bias.

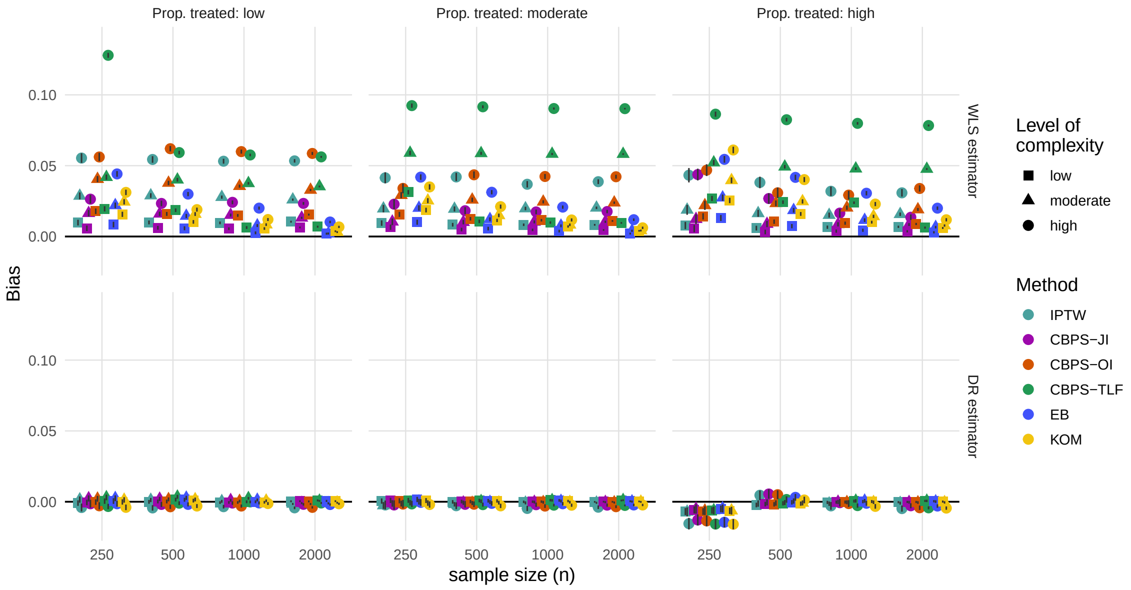

Figure 2: Bias of ATT estimates across simulation scenarios. Results are stratified by treatment prevalence (columns), estimator (rows), sample size (x-axis), complexity level (point shape), and weighting method (color). Vertical segments represent 95% Monte Carlo confidence intervals for the estimated bias.

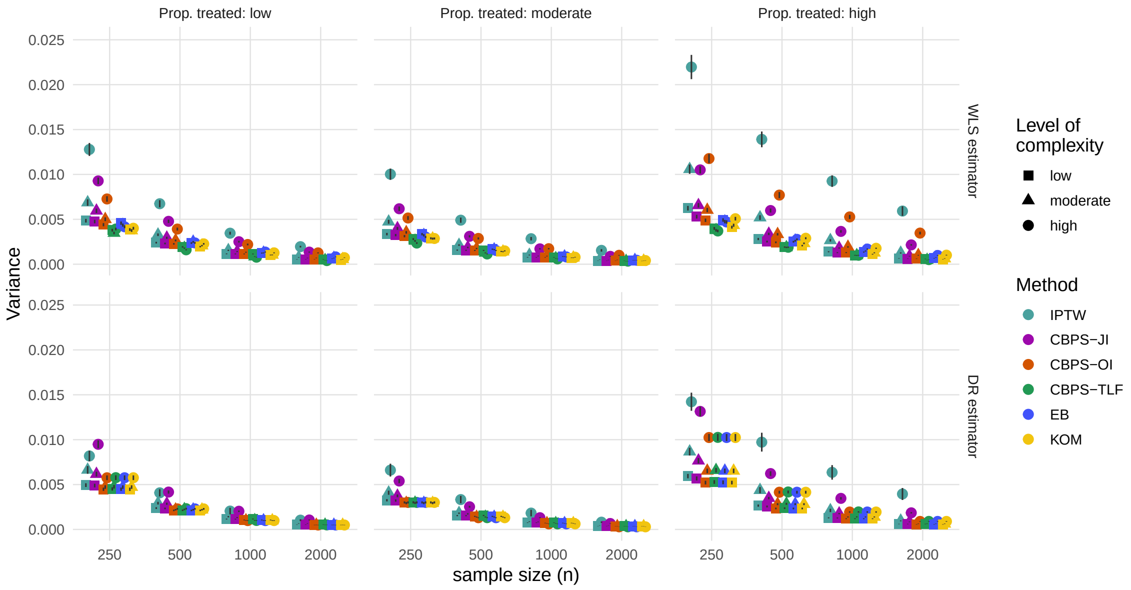

Figure 3: Variance of ATE estimates across simulation scenarios. Results are stratified by treatment prevalence (columns), estimator (rows), sample size (x-axis), complexity level (point shape), and weighting method (color). Vertical segments represent 95% Monte Carlo confidence intervals for the estimated variance.

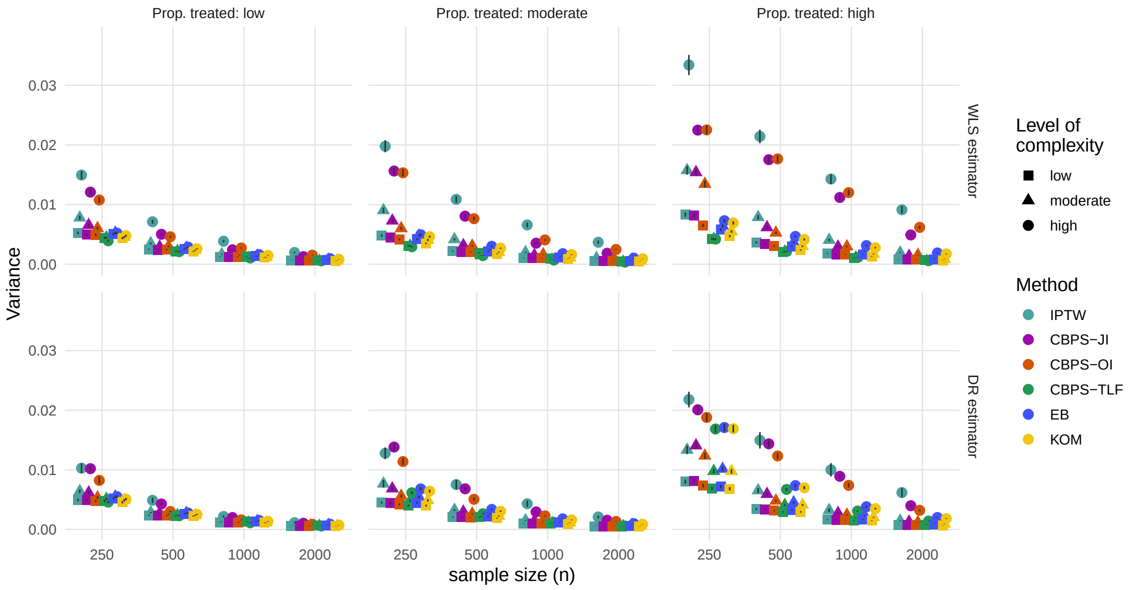

Figure 4: Variance of ATT estimates across simulation scenarios. Results are stratified by treatment prevalence (columns), estimator (rows), sample size (x-axis), complexity level (point shape), and weighting method (color). Vertical segments represent 95% Monte Carlo confidence intervals for the estimated variance.

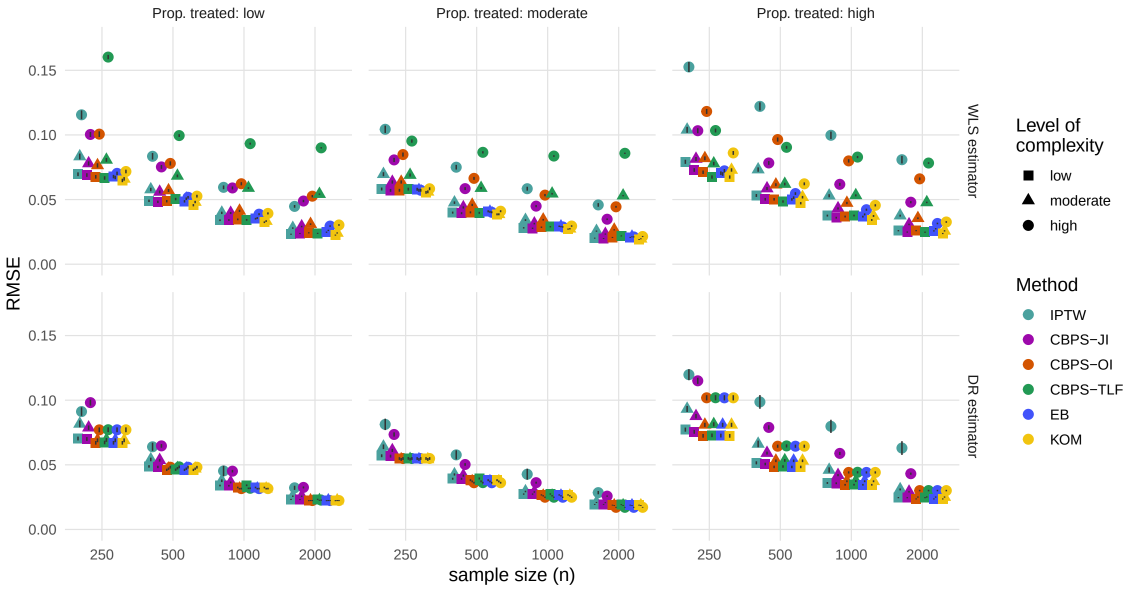

Figure 5: Root mean squared error (RMSE) of ATE estimates across simulation scenarios. Results are stratified by treatment prevalence (columns), estimator (rows), sample size (x-axis), complexity level (point shape), and weighting method (color). Vertical segments represent 95% Monte Carlo confidence intervals for the estimated RMSE.

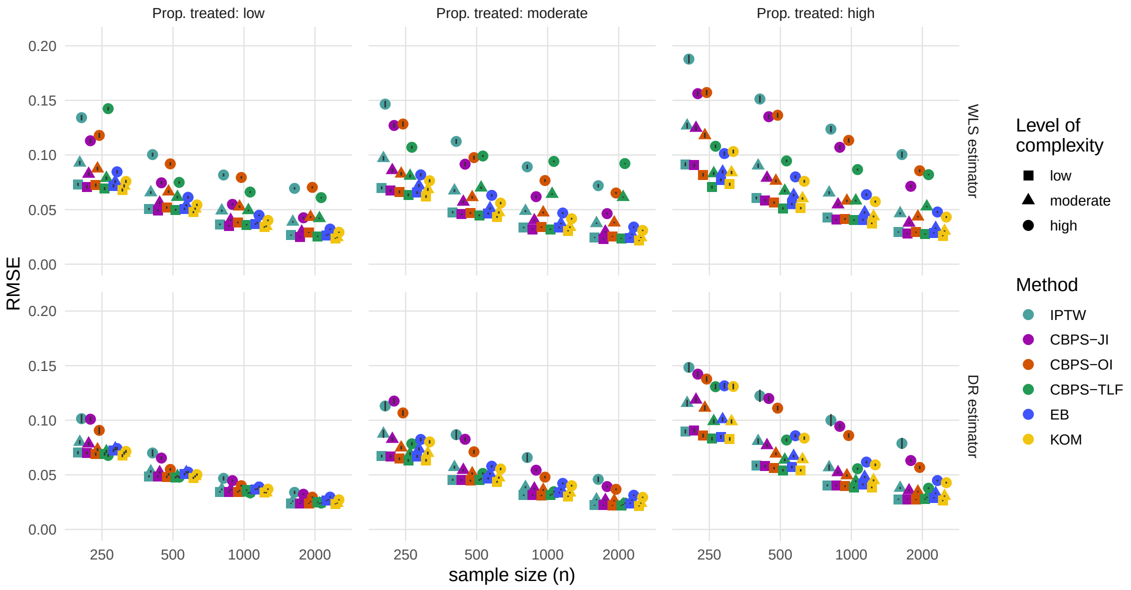

Figure 6: Root mean squared error (RMSE) of ATT estimates across simulation scenarios. Results are stratified by treatment prevalence (columns), estimator (rows), sample size (x-axis), complexity level (point shape), and weighting method (color). Vertical segments represent 95% Monte Carlo confidence intervals for the estimated RMSE.

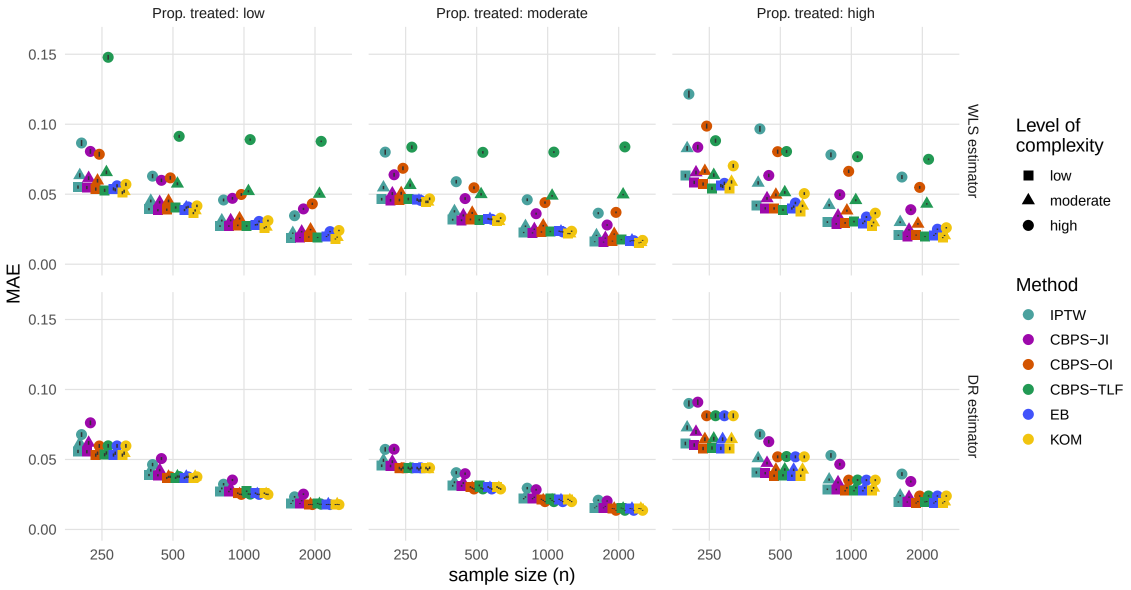

Figure 7: Mean absolute error (MAE) of ATE estimates across simulation scenarios. Results are stratified by treatment prevalence (columns), estimator (rows), sample size (x-axis), complexity level (point shape), and weighting method (color). Vertical segments represent 95% Monte Carlo confidence intervals for the estimated MAE.

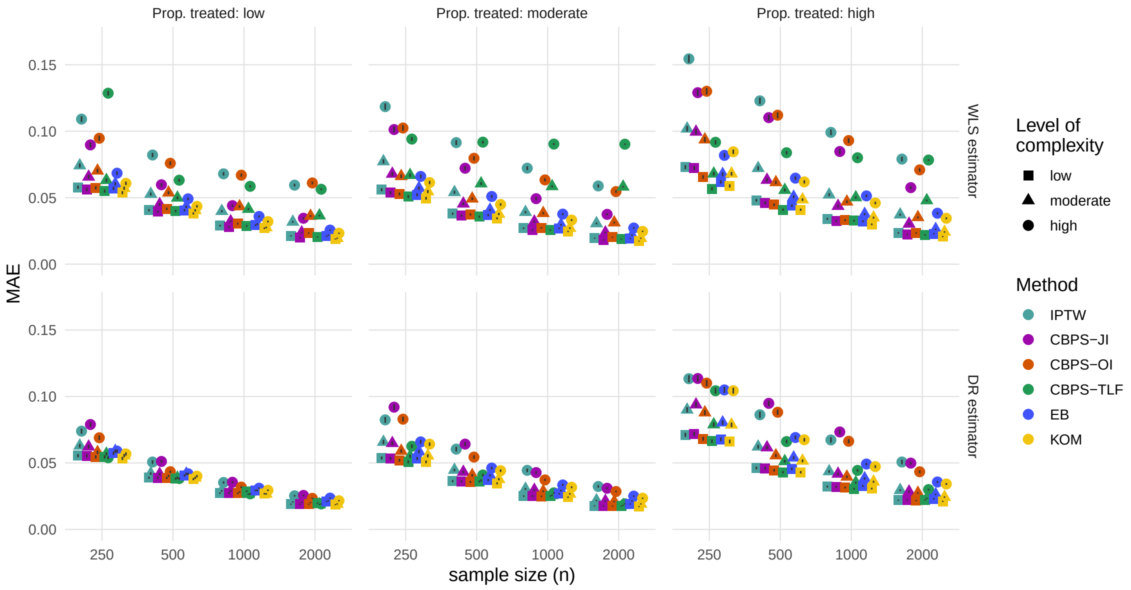

Figure 8: Mean absolute error (MAE) of ATT estimates across simulation scenarios. Results are stratified by treatment prevalence (columns), estimator (rows), sample size (x-axis), complexity level (point shape), and weighting method (color). Vertical segments represent 95% Monte Carlo confidence intervals for the estimated MAE.

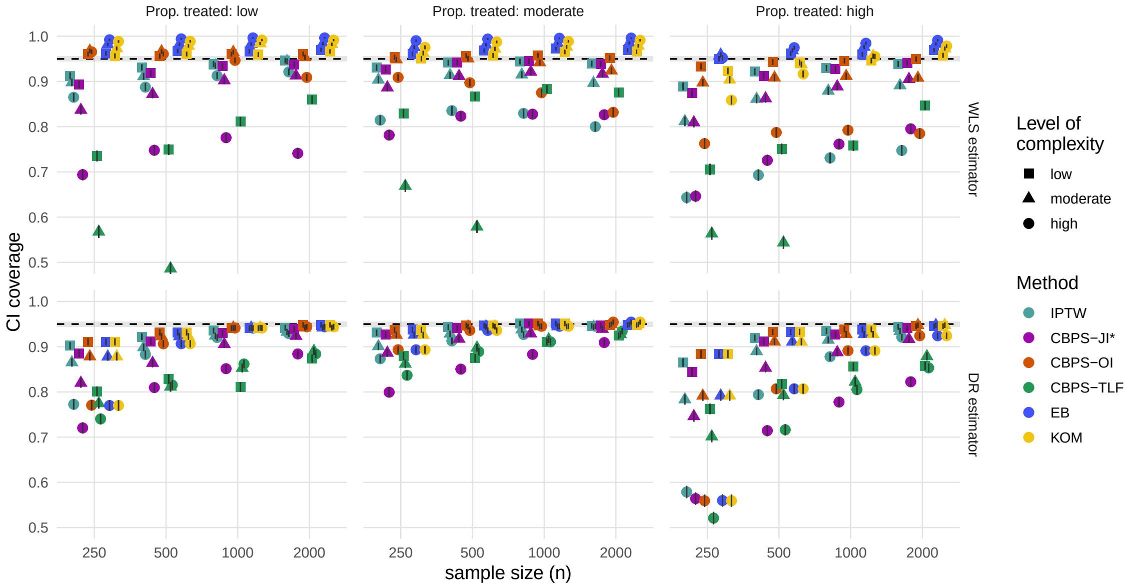

Figure 9: Empirical coverage of the 95% confidence interval for the ATE estimate across simulation scenarios. Results are stratified by treatment prevalence (columns), estimator (rows), sample size (x-axis), complexity level (point shape), and weighting method (color). Vertical segments represent 95% Monte Carlo confidence intervals for the estimated coverage. The black dashed line is the nominal coverage level. The shaded reference band indicates the range compatible with nominal 95% coverage up to Monte Carlo error, based on 5000 simulation replicates, corresponding to approximately 94.4% to 95.6%.

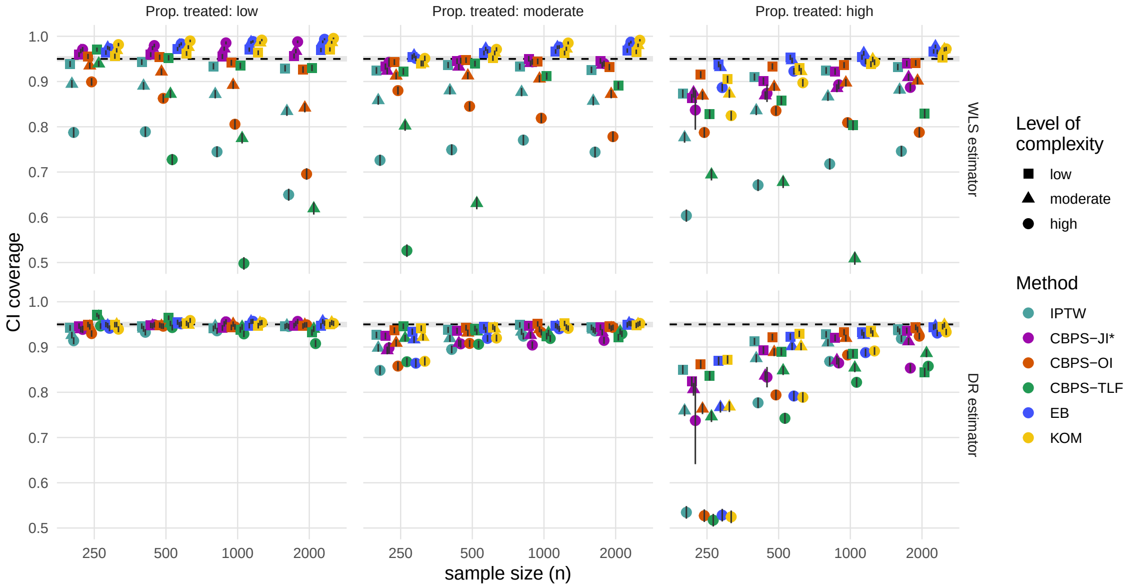

Figure 10: Empirical coverage of the 95% confidence interval for the ATT estimate across simulation scenarios. Results are stratified by treatment prevalence (columns), estimator (rows), sample size (x-axis), complexity level (point shape), and weighting method (color). Vertical segments represent 95% Monte Carlo confidence intervals for the estimated coverage. The black dashed line is the nominal coverage level. The shaded reference band indicates the range compatible with nominal 95% coverage up to Monte Carlo error, based on 5000 simulation replicates, corresponding to approximately 94.4% to 95.6%.

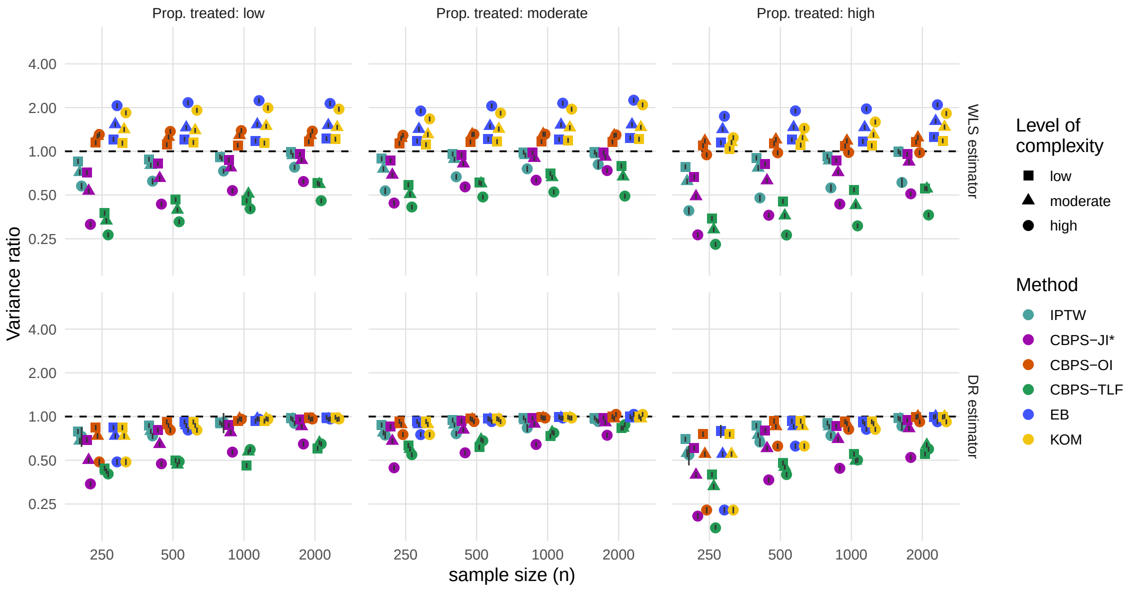

Figure 11: Variance ratio for the ATE estimates across simulation scenarios. Results are stratified by treatment prevalence (columns), estimator (rows), sample size (x-axis), complexity level (point shape), and weighting method (color). Values below 1 indicate that the average estimated variance is smaller than the empirical Monte Carlo variance of the point estimates, whereas values above 1 indicate variance overestimation. Vertical segments represent 95% Monte Carlo confidence intervals for the estimated variance ratio.

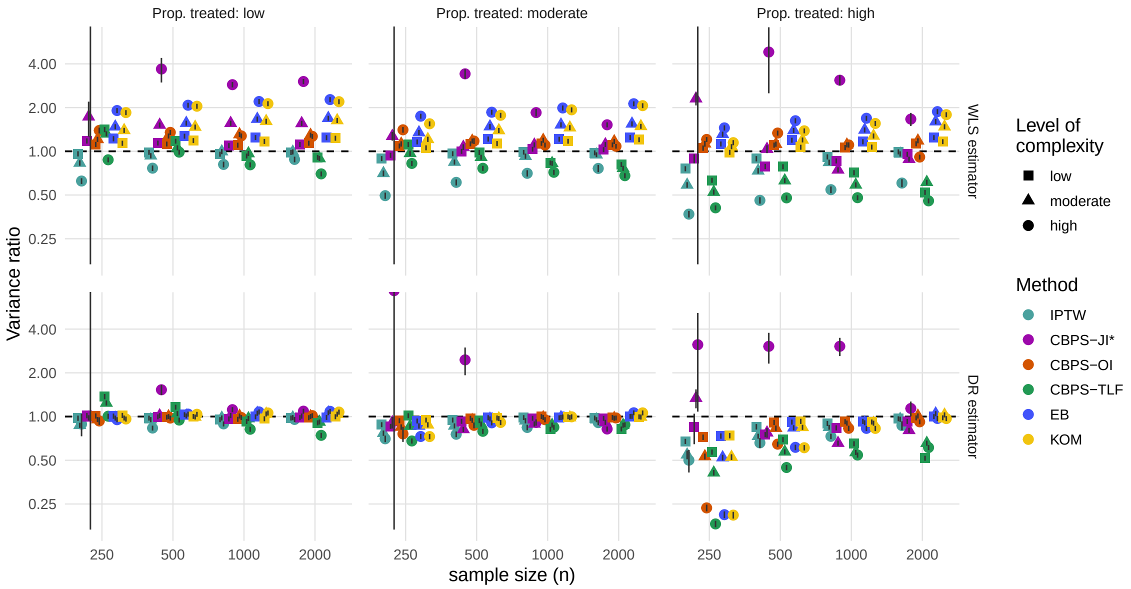

Figure 12: Variance ratio for the ATT estimates across simulation scenarios. Results are stratified by treatment prevalence (columns), estimator (rows), sample size (x-axis), complexity level (point shape), and weighting method (color). Values below 1 indicate that the average estimated variance is smaller than the empirical Monte Carlo variance of the point estimates, whereas values above 1 indicate variance overestimation. Vertical segments represent 95% Monte Carlo confidence intervals for the estimated variance ratio.

1.3 Metrics tables

In this section, we report the main simulation results for the metric tables. For each scenario, we show bias, variance, RMSE, MAE, empirical coverage of the 95% confidence interval, and the variance ratio for each combination of estimand, estimator, and weighting method. Values are reported as the estimate followed by its Monte Carlo standard error in parentheses. The missingness is also reported, the proportions of missing point estimates and missing standard-error estimates, respectively. Results from the sensitivity analysis are available on the GitHub repository for this article (https://github.com/EtiennePeyrot/benchmark_balancing_methods) along with an R shiny application to visualize and better navigate the results.

Because the tables are numerous, the section begins with an index organized by sample size, proportion treated, and complexity level. Each entry links to the corresponding table.

Scenario index

Sample size $n = 250$

Proportion treated: low

Complexity: low: Table 4, p. 17
Complexity: moderate: Table 5, p. 18
Complexity: high: Table 6, p. 19

Proportion treated: moderate

Complexity: low: Table 7, p. 20
Complexity: moderate: Table 8, p. 21
Complexity: high: Table 9, p. 22

Proportion treated: high

Complexity: low: Table 10, p. 23
Complexity: moderate: Table 11, p. 24
Complexity: high: Table 12, p. 25

Sample size $n = 500$

Proportion treated: low

Complexity: low: Table 13, p. 26
Complexity: moderate: Table 14, p. 27
Complexity: high: Table 15, p. 28

Proportion treated: moderate

Complexity: low: Table 16, p. 29
Complexity: moderate: Table 17, p. 30
Complexity: high: Table 18, p. 31

Proportion treated: high

Complexity: low: Table 19, p. 32
Complexity: moderate: Table 20, p. 33
Complexity: high: Table 21, p. 34

Sample size $n = 1000$

Proportion treated: low

Complexity: low: Table 22, p. 35
Complexity: moderate: Table 23, p. 36

Complexity: high: Table 24, p. 37
Proportion treated: moderate
Complexity: low: Table 25, p. 38
Complexity: moderate: Table 26, p. 39
Complexity: high: Table 27, p. 40
Proportion treated: high
Complexity: low: Table 28, p. 41
Complexity: moderate: Table 29, p. 42
Complexity: high: Table 30, p. 43

Sample size $n = 2000$
Proportion treated: low
Complexity: low: Table 31, p. 44
Complexity: moderate: Table 32, p. 45
Complexity: high: Table 33, p. 46
Proportion treated: moderate
Complexity: low: Table 34, p. 47
Complexity: moderate: Table 35, p. 48
Complexity: high: Table 36, p. 49
Proportion treated: high
Complexity: low: Table 37, p. 50
Complexity: moderate: Table 38, p. 51
Complexity: high: Table 39, p. 52

Tables

Table 4: Scenario: $n = 250$, proportion treated: low; complexity: low; treatment-effect level: none. Values are reported as estimate (Monte Carlo standard error), except for the missingness columns, which are reported without Monte Carlo standard error.

Estimand	Estimator	Method	Bias	Var	RMSE	MAE	Coverage	Var. ratio	Missingness	
									Point	SE
ATE	WLS	IPTW	0.001 ($< 10^{-3}$)	0.005 ($< 10^{-3}$)	0.070 ($< 10^{-3}$)	0.055 ($< 10^{-3}$)	0.912 (0.004)	0.853 (0.018)	0.000	0.000
		CBPS-JI	0.003 ($< 10^{-3}$)	0.005 ($< 10^{-3}$)	0.069 ($< 10^{-3}$)	0.055 ($< 10^{-3}$)	0.893 (0.004)	0.714 (0.015)	0.000	0.000
		CBPS-OI	0.013 ($< 10^{-3}$)	0.004 ($< 10^{-3}$)	0.068 ($< 10^{-3}$)	0.054 ($< 10^{-3}$)	0.961 (0.003)	1.149 (0.023)	0.000	0.000
		CBPS-TLF	0.026 ($< 10^{-3}$)	0.004 ($< 10^{-3}$)	0.067 ($< 10^{-3}$)	0.053 ($< 10^{-3}$)	0.735 (0.006)	0.376 (0.008)	0.000	0.000
		EB	0.005 ($< 10^{-3}$)	0.005 ($< 10^{-3}$)	0.068 ($< 10^{-3}$)	0.054 ($< 10^{-3}$)	0.962 (0.003)	1.207 (0.025)	0.000	0.000
		KOM	0.018 ($< 10^{-3}$)	0.004 ($< 10^{-3}$)	0.065 ($< 10^{-3}$)	0.051 ($< 10^{-3}$)	0.957 (0.003)	1.138 (0.024)	0.000	0.000
	DR	IPTW	0.004 ($< 10^{-3}$)	0.005 ($< 10^{-3}$)	0.070 ($< 10^{-3}$)	0.056 ($< 10^{-3}$)	0.902 (0.004)	0.790 (0.017)	0.000	0.001
		CBPS-JI	0.003 ($< 10^{-3}$)	0.005 ($< 10^{-3}$)	0.070 ($< 10^{-3}$)	0.056 ($< 10^{-3}$)	0.885 (0.005)	0.692 (0.015)	0.000	0.001
		CBPS-OI	0.004 ($< 10^{-3}$)	0.004 ($< 10^{-3}$)	0.067 ($< 10^{-3}$)	0.053 ($< 10^{-3}$)	0.911 (0.004)	0.840 (0.018)	0.001	0.001
		CBPS-TLF	0.004 ($< 10^{-3}$)	0.005 ($< 10^{-3}$)	0.067 ($< 10^{-3}$)	0.053 ($< 10^{-3}$)	0.801 (0.006)	0.440 (0.009)	0.000	0.001
		EB	0.004 ($< 10^{-3}$)	0.004 ($< 10^{-3}$)	0.067 ($< 10^{-3}$)	0.053 ($< 10^{-3}$)	0.911 (0.004)	0.840 (0.018)	0.001	0.001
		KOM	0.004 ($< 10^{-3}$)	0.004 ($< 10^{-3}$)	0.067 ($< 10^{-3}$)	0.053 ($< 10^{-3}$)	0.911 (0.004)	0.840 (0.018)	0.001	0.001
ATT	WLS	IPTW	0.010 (0.001)	0.005 ($< 10^{-3}$)	0.073 ($< 10^{-3}$)	0.058 ($< 10^{-3}$)	0.939 (0.003)	0.961 (0.020)	0.000	0.000
		CBPS-JI	0.006 ($< 10^{-3}$)	0.005 ($< 10^{-3}$)	0.071 ($< 10^{-3}$)	0.056 ($< 10^{-3}$)	0.959 (0.003)	1.179 (0.025)	0.000	0.000
		CBPS-OI	0.018 ($< 10^{-3}$)	0.005 ($< 10^{-3}$)	0.072 ($< 10^{-3}$)	0.057 ($< 10^{-3}$)	0.955 (0.003)	1.115 (0.023)	0.000	0.000
		CBPS-TLF	0.020 ($< 10^{-3}$)	0.004 ($< 10^{-3}$)	0.069 ($< 10^{-3}$)	0.055 ($< 10^{-3}$)	0.970 (0.002)	1.411 (0.029)	0.000	0.000
		EB	0.008 (0.001)	0.005 ($< 10^{-3}$)	0.072 ($< 10^{-3}$)	0.057 ($< 10^{-3}$)	0.964 (0.003)	1.221 (0.026)	0.000	0.000
		KOM	0.016 ($< 10^{-3}$)	0.004 ($< 10^{-3}$)	0.068 ($< 10^{-3}$)	0.054 ($< 10^{-3}$)	0.956 (0.003)	1.137 (0.024)	0.000	0.000
	DR	IPTW	$< 10^{-3}$ ($< 10^{-3}$)	0.005 ($< 10^{-3}$)	0.070 ($< 10^{-3}$)	0.055 ($< 10^{-3}$)	0.942 (0.003)	0.975 (0.021)	0.000	0.001
		CBPS-JI	$< 10^{-3}$ ($< 10^{-3}$)	0.005 ($< 10^{-3}$)	0.070 ($< 10^{-3}$)	0.055 ($< 10^{-3}$)	0.946 (0.003)	1.015 (0.022)	0.000	0.001
		CBPS-OI	$< 10^{-3}$ ($< 10^{-3}$)	0.005 ($< 10^{-3}$)	0.069 ($< 10^{-3}$)	0.055 ($< 10^{-3}$)	0.950 (0.003)	1.009 (0.021)	0.001	0.001
		CBPS-TLF	$< 10^{-3}$ ($< 10^{-3}$)	0.005 ($< 10^{-3}$)	0.069 ($< 10^{-3}$)	0.055 ($< 10^{-3}$)	0.971 (0.002)	1.372 (0.029)	0.000	0.001
		EB	$< 10^{-3}$ (0.001)	0.005 ($< 10^{-3}$)	0.072 ($< 10^{-3}$)	0.057 ($< 10^{-3}$)	0.950 (0.003)	1.007 (0.021)	0.001	0.001
		KOM	$< 10^{-3}$ ($< 10^{-3}$)	0.005 ($< 10^{-3}$)	0.068 ($< 10^{-3}$)	0.053 ($< 10^{-3}$)	0.949 (0.003)	1.018 (0.021)	0.001	0.001

Table 5: Scenario: $n = 250$, proportion treated: low; complexity: moderate; treatment-effect level: none. Values are reported as estimate (Monte Carlo standard error), except for the missingness columns, which are reported without Monte Carlo standard error.

Estimand	Estimator	Method	Bias	Var	RMSE	MAE	Coverage	Var. ratio	Missingness Point	Missingness SE
ATE	WLS	IPTW	0.009 (0.001)	0.007 ($< 10^{-3}$)	0.083 (0.001)	0.063 ($< 10^{-3}$)	0.898 (0.004)	0.717 (0.017)	0.000	0.000
		CBPS-JI	0.011 (0.001)	0.006 ($< 10^{-3}$)	0.078 ($< 10^{-3}$)	0.061 ($< 10^{-3}$)	0.837 (0.005)	0.533 (0.011)	0.000	0.000
		CBPS-OI	0.029 (0.001)	0.005 ($< 10^{-3}$)	0.076 ($< 10^{-3}$)	0.060 ($< 10^{-3}$)	0.968 (0.003)	1.272 (0.026)	0.000	0.000
		CBPS-TLF	0.055 ($< 10^{-3}$)	0.003 ($< 10^{-3}$)	0.081 ($< 10^{-3}$)	0.066 ($< 10^{-3}$)	0.567 (0.007)	0.332 (0.007)	0.000	0.000
		EB	0.012 ($< 10^{-3}$)	0.004 ($< 10^{-3}$)	0.067 ($< 10^{-3}$)	0.053 ($< 10^{-3}$)	0.982 (0.002)	1.531 (0.031)	0.000	0.000
		KOM	0.025 ($< 10^{-3}$)	0.004 ($< 10^{-3}$)	0.066 ($< 10^{-3}$)	0.052 ($< 10^{-3}$)	0.973 (0.002)	1.413 (0.029)	0.000	0.000
	DR	IPTW	0.006 (0.001)	0.007 ($< 10^{-3}$)	0.082 (0.001)	0.062 ($< 10^{-3}$)	0.865 (0.005)	0.680 (0.017)	0.000	0.002
		CBPS-JI	0.006 (0.001)	0.006 ($< 10^{-3}$)	0.079 ($< 10^{-3}$)	0.062 ($< 10^{-3}$)	0.820 (0.005)	0.501 (0.011)	0.000	0.004
		CBPS-OI	0.006 ($< 10^{-3}$)	0.005 ($< 10^{-3}$)	0.069 ($< 10^{-3}$)	0.054 ($< 10^{-3}$)	0.878 (0.005)	0.735 (0.016)	0.002	0.002
		CBPS-TLF	0.006 ($< 10^{-3}$)	0.005 ($< 10^{-3}$)	0.069 ($< 10^{-3}$)	0.055 ($< 10^{-3}$)	0.775 (0.006)	0.421 (0.009)	0.000	0.002
		EB	0.006 ($< 10^{-3}$)	0.005 ($< 10^{-3}$)	0.069 ($< 10^{-3}$)	0.054 ($< 10^{-3}$)	0.878 (0.005)	0.735 (0.016)	0.002	0.002
		KOM	0.006 ($< 10^{-3}$)	0.005 ($< 10^{-3}$)	0.069 ($< 10^{-3}$)	0.054 ($< 10^{-3}$)	0.878 (0.005)	0.735 (0.016)	0.002	0.002
ATT	WLS	IPTW	0.029 (0.001)	0.008 ($< 10^{-3}$)	0.093 ($< 10^{-3}$)	0.074 ($< 10^{-3}$)	0.895 (0.004)	0.831 (0.018)	0.000	0.000
		CBPS-JI	0.016 (0.001)	0.007 ($< 10^{-3}$)	0.083 ($< 10^{-3}$)	0.066 ($< 10^{-3}$)	0.967 (0.003)	1.729 (0.238)	0.000	0.001
		CBPS-OI	0.040 (0.001)	0.006 ($< 10^{-3}$)	0.087 ($< 10^{-3}$)	0.070 ($< 10^{-3}$)	0.935 (0.003)	1.209 (0.025)	0.000	0.000
		CBPS-TLF	0.042 ($< 10^{-3}$)	0.004 ($< 10^{-3}$)	0.079 ($< 10^{-3}$)	0.063 ($< 10^{-3}$)	0.939 (0.003)	1.319 (0.027)	0.000	0.000
		EB	0.022 (0.001)	0.005 ($< 10^{-3}$)	0.076 ($< 10^{-3}$)	0.060 ($< 10^{-3}$)	0.975 (0.002)	1.477 (0.030)	0.000	0.000
		KOM	0.024 ($< 10^{-3}$)	0.005 ($< 10^{-3}$)	0.072 ($< 10^{-3}$)	0.057 ($< 10^{-3}$)	0.969 (0.002)	1.394 (0.028)	0.000	0.000
	DR	IPTW	0.002 (0.001)	0.006 ($< 10^{-3}$)	0.080 ($< 10^{-3}$)	0.063 ($< 10^{-3}$)	0.926 (0.004)	0.871 (0.018)	0.000	0.002
		CBPS-JI	0.002 (0.001)	0.006 ($< 10^{-3}$)	0.079 ($< 10^{-3}$)	0.062 ($< 10^{-3}$)	0.939 (0.003)	0.980 (0.021)	0.000	0.008
		CBPS-OI	0.002 (0.001)	0.005 ($< 10^{-3}$)	0.073 ($< 10^{-3}$)	0.058 ($< 10^{-3}$)	0.945 (0.003)	0.954 (0.020)	0.002	0.002
		CBPS-TLF	0.003 (0.001)	0.005 ($< 10^{-3}$)	0.071 ($< 10^{-3}$)	0.057 ($< 10^{-3}$)	0.967 (0.003)	1.240 (0.026)	0.000	0.002
		EB	0.002 (0.001)	0.005 ($< 10^{-3}$)	0.074 ($< 10^{-3}$)	0.059 ($< 10^{-3}$)	0.947 (0.003)	0.977 (0.020)	0.002	0.002
		KOM	0.001 ($< 10^{-3}$)	0.005 ($< 10^{-3}$)	0.070 ($< 10^{-3}$)	0.056 ($< 10^{-3}$)	0.950 (0.003)	0.977 (0.020)	0.002	0.002

Table 6: Scenario: $n = 250$, proportion treated: low; complexity: high; treatment-effect level: none. Values are reported as estimate (Monte Carlo standard error), except for the missingness columns, which are reported without Monte Carlo standard error.

Estimand	Estimator	Method	Bias	Var	RMSE	MAE	Coverage	Var. ratio	Missingness Point	Missingness SE
ATE	WLS	IPTW	0.024 (0.002)	0.013 ($< 10^{-3}$)	0.116 (0.002)	0.087 (0.001)	0.865 (0.005)	0.577 (0.015)	0.000	0.000
		CBPS-JI	0.028 (0.001)	0.009 ($< 10^{-3}$)	0.100 ($< 10^{-3}$)	0.081 ($< 10^{-3}$)	0.694 (0.007)	0.314 (0.007)	0.000	0.000
		CBPS-OI	0.053 (0.001)	0.007 ($< 10^{-3}$)	0.101 (0.001)	0.079 ($< 10^{-3}$)	0.966 (0.003)	1.306 (0.027)	0.002	0.002
		CBPS-TLF	0.148 ($< 10^{-3}$)	0.004 ($< 10^{-3}$)	0.160 ($< 10^{-3}$)	0.148 ($< 10^{-3}$)	0.084 (0.004)	0.266 (0.005)	0.000	0.000
		EB	0.028 ($< 10^{-3}$)	0.004 ($< 10^{-3}$)	0.070 ($< 10^{-3}$)	0.056 ($< 10^{-3}$)	0.992 (0.001)	2.061 (0.041)	0.000	0.000
		KOM	0.034 ($< 10^{-3}$)	0.004 ($< 10^{-3}$)	0.072 ($< 10^{-3}$)	0.057 ($< 10^{-3}$)	0.988 (0.002)	1.841 (0.037)	0.000	0.000
	DR	IPTW	0.012 (0.001)	0.008 ($< 10^{-3}$)	0.091 (0.002)	0.068 ($< 10^{-3}$)	0.773 (0.006)	0.730 (0.057)	0.000	0.010
		CBPS-JI	0.012 (0.001)	0.009 ($< 10^{-3}$)	0.098 (0.001)	0.076 ($< 10^{-3}$)	0.720 (0.006)	0.344 (0.008)	0.000	0.027
		CBPS-OI	0.014 (0.001)	0.006 ($< 10^{-3}$)	0.077 ($< 10^{-3}$)	0.060 ($< 10^{-3}$)	0.771 (0.006)	0.487 (0.012)	0.011	0.011
		CBPS-TLF	0.014 (0.001)	0.006 ($< 10^{-3}$)	0.077 ($< 10^{-3}$)	0.060 ($< 10^{-3}$)	0.740 (0.006)	0.403 (0.010)	0.000	0.011
		EB	0.014 (0.001)	0.006 ($< 10^{-3}$)	0.077 ($< 10^{-3}$)	0.060 ($< 10^{-3}$)	0.770 (0.006)	0.487 (0.012)	0.010	0.010
		KOM	0.014 (0.001)	0.006 ($< 10^{-3}$)	0.077 ($< 10^{-3}$)	0.060 ($< 10^{-3}$)	0.770 (0.006)	0.487 (0.012)	0.010	0.010
ATT	WLS	IPTW	0.055 (0.002)	0.015 ($< 10^{-3}$)	0.134 (0.001)	0.109 (0.001)	0.787 (0.006)	0.624 (0.014)	0.000	0.000
		CBPS-JI	0.026 (0.002)	0.012 ($< 10^{-3}$)	0.113 (0.001)	0.090 ($< 10^{-3}$)	0.971 (0.003)	188456.148 (151818.910)	0.000	0.197
		CBPS-OI	0.056 (0.001)	0.011 ($< 10^{-3}$)	0.118 (0.001)	0.095 ($< 10^{-3}$)	0.899 (0.004)	1.391 (0.032)	0.002	0.002
		CBPS-TLF	0.128 ($< 10^{-3}$)	0.004 ($< 10^{-3}$)	0.142 ($< 10^{-3}$)	0.129 ($< 10^{-3}$)	0.408 (0.007)	0.874 (0.017)	0.000	0.000
		EB	0.044 (0.001)	0.005 ($< 10^{-3}$)	0.085 ($< 10^{-3}$)	0.068 ($< 10^{-3}$)	0.973 (0.002)	1.908 (0.039)	0.000	0.000
		KOM	0.031 ($< 10^{-3}$)	0.005 ($< 10^{-3}$)	0.076 ($< 10^{-3}$)	0.061 ($< 10^{-3}$)	0.982 (0.002)	1.847 (0.037)	0.000	0.000
	DR	IPTW	-0.004 (0.001)	0.010 ($< 10^{-3}$)	0.101 (0.002)	0.074 ($< 10^{-3}$)	0.914 (0.004)	0.887 (0.080)	0.001	0.008
		CBPS-JI	-0.001 (0.001)	0.010 ($< 10^{-3}$)	0.101 (0.001)	0.079 ($< 10^{-3}$)	0.939 (0.004)	42.480 (39.193)	0.000	0.329
		CBPS-OI	-0.003 (0.001)	0.008 ($< 10^{-3}$)	0.091 (0.002)	0.069 ($< 10^{-3}$)	0.930 (0.004)	0.932 (0.028)	0.010	0.010
		CBPS-TLF	-0.003 ($< 10^{-3}$)	0.005 ($< 10^{-3}$)	0.068 ($< 10^{-3}$)	0.054 ($< 10^{-3}$)	0.946 (0.003)	1.005 (0.021)	0.000	0.008
		EB	-0.001 (0.001)	0.005 ($< 10^{-3}$)	0.074 ($< 10^{-3}$)	0.059 ($< 10^{-3}$)	0.941 (0.003)	0.951 (0.019)	0.007	0.007
		KOM	-0.004 (0.001)	0.005 ($< 10^{-3}$)	0.071 ($< 10^{-3}$)	0.057 ($< 10^{-3}$)	0.939 (0.003)	0.962 (0.020)	0.007	0.007

Table 7: Scenario: $n = 250$, proportion treated: moderate; complexity: low; treatment-effect level: none. Values are reported as estimate (Monte Carlo standard error), except for the missingness columns, which are reported without Monte Carlo standard error.

Estimand	Estimator	Method	Bias	Var	RMSE	MAE	Coverage	Var. ratio	Missingness Point	Missingness SE
ATE	WLS	IPTW	0.006 ($< 10^{-3}$)	0.003 ($< 10^{-3}$)	0.058 ($< 10^{-3}$)	0.046 ($< 10^{-3}$)	0.931 (0.004)	0.889 (0.018)	0.000	0.000
		CBPS-JI	0.005 ($< 10^{-3}$)	0.003 ($< 10^{-3}$)	0.057 ($< 10^{-3}$)	0.046 ($< 10^{-3}$)	0.927 (0.004)	0.863 (0.018)	0.000	0.000
		CBPS-OI	0.012 ($< 10^{-3}$)	0.003 ($< 10^{-3}$)	0.057 ($< 10^{-3}$)	0.046 ($< 10^{-3}$)	0.954 (0.003)	1.133 (0.023)	0.000	0.000
		CBPS-TLF	0.024 ($< 10^{-3}$)	0.003 ($< 10^{-3}$)	0.058 ($< 10^{-3}$)	0.047 ($< 10^{-3}$)	0.829 (0.005)	0.587 (0.012)	0.000	0.000
		EB	0.004 ($< 10^{-3}$)	0.003 ($< 10^{-3}$)	0.058 ($< 10^{-3}$)	0.046 ($< 10^{-3}$)	0.961 (0.003)	1.177 (0.024)	0.000	0.000
		KOM	0.014 ($< 10^{-3}$)	0.003 ($< 10^{-3}$)	0.056 ($< 10^{-3}$)	0.045 ($< 10^{-3}$)	0.950 (0.003)	1.112 (0.022)	0.000	0.000
	DR	IPTW	0.001 ($< 10^{-3}$)	0.003 ($< 10^{-3}$)	0.057 ($< 10^{-3}$)	0.046 ($< 10^{-3}$)	0.931 (0.004)	0.874 (0.018)	0.000	0.000
		CBPS-JI	0.001 ($< 10^{-3}$)	0.003 ($< 10^{-3}$)	0.057 ($< 10^{-3}$)	0.045 ($< 10^{-3}$)	0.927 (0.004)	0.852 (0.017)	0.000	0.000
		CBPS-OI	$< 10^{-3}$ ($< 10^{-3}$)	0.003 ($< 10^{-3}$)	0.055 ($< 10^{-3}$)	0.044 ($< 10^{-3}$)	0.937 (0.003)	0.924 (0.019)	0.000	0.000
		CBPS-TLF	0.001 ($< 10^{-3}$)	0.003 ($< 10^{-3}$)	0.055 ($< 10^{-3}$)	0.044 ($< 10^{-3}$)	0.879 (0.005)	0.633 (0.013)	0.000	0.000
		EB	$< 10^{-3}$ ($< 10^{-3}$)	0.003 ($< 10^{-3}$)	0.055 ($< 10^{-3}$)	0.044 ($< 10^{-3}$)	0.938 (0.003)	0.924 (0.019)	0.000	0.000
		KOM	$< 10^{-3}$ ($< 10^{-3}$)	0.003 ($< 10^{-3}$)	0.055 ($< 10^{-3}$)	0.044 ($< 10^{-3}$)	0.937 (0.003)	0.924 (0.019)	0.000	0.000
ATT	WLS	IPTW	0.009 ($< 10^{-3}$)	0.005 ($< 10^{-3}$)	0.070 ($< 10^{-3}$)	0.056 ($< 10^{-3}$)	0.924 (0.004)	0.891 (0.018)	0.000	0.000
		CBPS-JI	0.007 ($< 10^{-3}$)	0.004 ($< 10^{-3}$)	0.067 ($< 10^{-3}$)	0.054 ($< 10^{-3}$)	0.933 (0.004)	0.939 (0.019)	0.000	0.000
		CBPS-OI	0.016 ($< 10^{-3}$)	0.004 ($< 10^{-3}$)	0.066 ($< 10^{-3}$)	0.053 ($< 10^{-3}$)	0.943 (0.003)	1.089 (0.022)	0.000	0.000
		CBPS-TLF	0.031 ($< 10^{-3}$)	0.003 ($< 10^{-3}$)	0.063 ($< 10^{-3}$)	0.051 ($< 10^{-3}$)	0.922 (0.004)	1.114 (0.022)	0.000	0.000
		EB	0.010 ($< 10^{-3}$)	0.004 ($< 10^{-3}$)	0.066 ($< 10^{-3}$)	0.052 ($< 10^{-3}$)	0.954 (0.003)	1.161 (0.023)	0.000	0.000
		KOM	0.019 ($< 10^{-3}$)	0.003 ($< 10^{-3}$)	0.062 ($< 10^{-3}$)	0.050 ($< 10^{-3}$)	0.940 (0.003)	1.058 (0.021)	0.000	0.000
	DR	IPTW	$< 10^{-3}$ ($< 10^{-3}$)	0.005 ($< 10^{-3}$)	0.067 ($< 10^{-3}$)	0.054 ($< 10^{-3}$)	0.927 (0.004)	0.879 (0.018)	0.000	0.000
		CBPS-JI	$< 10^{-3}$ ($< 10^{-3}$)	0.004 ($< 10^{-3}$)	0.067 ($< 10^{-3}$)	0.053 ($< 10^{-3}$)	0.924 (0.004)	0.856 (0.017)	0.000	0.000
		CBPS-OI	$< 10^{-3}$ ($< 10^{-3}$)	0.004 ($< 10^{-3}$)	0.065 ($< 10^{-3}$)	0.052 ($< 10^{-3}$)	0.937 (0.003)	0.937 (0.019)	0.000	0.000
		CBPS-TLF	$< 10^{-3}$ ($< 10^{-3}$)	0.004 ($< 10^{-3}$)	0.063 ($< 10^{-3}$)	0.051 ($< 10^{-3}$)	0.946 (0.003)	1.014 (0.020)	0.000	0.000
		EB	0.002 ($< 10^{-3}$)	0.004 ($< 10^{-3}$)	0.067 ($< 10^{-3}$)	0.053 ($< 10^{-3}$)	0.934 (0.004)	0.932 (0.019)	0.000	0.000
		KOM	$< 10^{-3}$ ($< 10^{-3}$)	0.004 ($< 10^{-3}$)	0.063 ($< 10^{-3}$)	0.051 ($< 10^{-3}$)	0.941 (0.003)	0.946 (0.019)	0.000	0.000

Table 8: Scenario: $n = 250$, proportion treated: moderate; complexity: moderate; treatment-effect level: none. Values are reported as estimate (Monte Carlo standard error), except for the missingness columns, which are reported without Monte Carlo standard error.

Estimand	Estimator	Method	Bias	Var	RMSE	MAE	Coverage	Var. ratio	Missingness Point	Missingness SE
ATE	WLS	IPTW	0.011 ($< 10^{-3}$)	0.005 ($< 10^{-3}$)	0.070 ($< 10^{-3}$)	0.055 ($< 10^{-3}$)	0.904 (0.004)	0.756 (0.016)	0.000	0.000
		CBPS-JI	0.008 ($< 10^{-3}$)	0.004 ($< 10^{-3}$)	0.064 ($< 10^{-3}$)	0.050 ($< 10^{-3}$)	0.886 (0.004)	0.687 (0.014)	0.000	0.000
		CBPS-OI	0.022 ($< 10^{-3}$)	0.004 ($< 10^{-3}$)	0.064 ($< 10^{-3}$)	0.051 ($< 10^{-3}$)	0.949 (0.003)	1.232 (0.025)	0.000	0.000
		CBPS-TLF	0.047 ($< 10^{-3}$)	0.003 ($< 10^{-3}$)	0.069 ($< 10^{-3}$)	0.056 ($< 10^{-3}$)	0.669 (0.007)	0.506 (0.010)	0.000	0.000
		EB	0.007 ($< 10^{-3}$)	0.003 ($< 10^{-3}$)	0.057 ($< 10^{-3}$)	0.045 ($< 10^{-3}$)	0.976 (0.002)	1.421 (0.030)	0.000	0.000
		KOM	0.017 ($< 10^{-3}$)	0.003 ($< 10^{-3}$)	0.056 ($< 10^{-3}$)	0.045 ($< 10^{-3}$)	0.963 (0.003)	1.305 (0.027)	0.000	0.000
	DR	IPTW	-0.001 ($< 10^{-3}$)	0.004 ($< 10^{-3}$)	0.064 ($< 10^{-3}$)	0.049 ($< 10^{-3}$)	0.901 (0.004)	0.774 (0.016)	0.000	0.000
		CBPS-JI	$< 10^{-3}$ ($< 10^{-3}$)	0.004 ($< 10^{-3}$)	0.061 ($< 10^{-3}$)	0.048 ($< 10^{-3}$)	0.886 (0.004)	0.679 (0.014)	0.000	0.000
		CBPS-OI	$< 10^{-3}$ ($< 10^{-3}$)	0.003 ($< 10^{-3}$)	0.054 ($< 10^{-3}$)	0.043 ($< 10^{-3}$)	0.926 (0.004)	0.883 (0.019)	0.000	0.000
		CBPS-TLF	$< 10^{-3}$ ($< 10^{-3}$)	0.003 ($< 10^{-3}$)	0.054 ($< 10^{-3}$)	0.043 ($< 10^{-3}$)	0.862 (0.005)	0.597 (0.013)	0.000	0.000
		EB	$< 10^{-3}$ ($< 10^{-3}$)	0.003 ($< 10^{-3}$)	0.054 ($< 10^{-3}$)	0.043 ($< 10^{-3}$)	0.926 (0.004)	0.883 (0.019)	0.000	0.000
		KOM	$< 10^{-3}$ ($< 10^{-3}$)	0.003 ($< 10^{-3}$)	0.054 ($< 10^{-3}$)	0.043 ($< 10^{-3}$)	0.926 (0.004)	0.883 (0.019)	0.000	0.000
ATT	WLS	IPTW	0.020 (0.001)	0.009 ($< 10^{-3}$)	0.097 (0.001)	0.077 ($< 10^{-3}$)	0.858 (0.005)	0.705 (0.016)	0.000	0.000
		CBPS-JI	0.010 (0.001)	0.007 ($< 10^{-3}$)	0.086 ($< 10^{-3}$)	0.068 ($< 10^{-3}$)	0.923 (0.004)	1.271 (0.044)	0.000	0.009
		CBPS-OI	0.029 (0.001)	0.006 ($< 10^{-3}$)	0.083 ($< 10^{-3}$)	0.066 ($< 10^{-3}$)	0.913 (0.004)	1.125 (0.024)	0.000	0.000
		CBPS-TLF	0.059 ($< 10^{-3}$)	0.003 ($< 10^{-3}$)	0.081 ($< 10^{-3}$)	0.067 ($< 10^{-3}$)	0.802 (0.006)	0.969 (0.020)	0.000	0.000
		EB	0.020 ($< 10^{-3}$)	0.005 ($< 10^{-3}$)	0.072 ($< 10^{-3}$)	0.057 ($< 10^{-3}$)	0.957 (0.003)	1.346 (0.029)	0.000	0.000
		KOM	0.025 ($< 10^{-3}$)	0.004 ($< 10^{-3}$)	0.068 ($< 10^{-3}$)	0.055 ($< 10^{-3}$)	0.940 (0.003)	1.209 (0.025)	0.000	0.000
	DR	IPTW	-0.003 (0.001)	0.008 ($< 10^{-3}$)	0.088 (0.002)	0.066 ($< 10^{-3}$)	0.898 (0.004)	0.769 (0.016)	0.000	0.000
		CBPS-JI	-0.001 (0.001)	0.007 ($< 10^{-3}$)	0.083 ($< 10^{-3}$)	0.065 ($< 10^{-3}$)	0.892 (0.004)	0.910 (0.042)	0.000	0.009
		CBPS-OI	-0.001 (0.001)	0.006 ($< 10^{-3}$)	0.075 ($< 10^{-3}$)	0.059 ($< 10^{-3}$)	0.909 (0.004)	0.847 (0.018)	0.000	0.000
		CBPS-TLF	$< 10^{-3}$ ($< 10^{-3}$)	0.005 ($< 10^{-3}$)	0.069 ($< 10^{-3}$)	0.055 ($< 10^{-3}$)	0.920 (0.004)	0.859 (0.018)	0.000	0.000
		EB	$< 10^{-3}$ (0.001)	0.005 ($< 10^{-3}$)	0.074 ($< 10^{-3}$)	0.059 ($< 10^{-3}$)	0.916 (0.004)	0.862 (0.018)	0.000	0.000
		KOM	$< 10^{-3}$ ($< 10^{-3}$)	0.005 ($< 10^{-3}$)	0.070 ($< 10^{-3}$)	0.055 ($< 10^{-3}$)	0.922 (0.004)	0.877 (0.019)	0.000	0.000

Table 9: Scenario: $n = 250$, proportion treated: moderate; complexity: high; treatment-effect level: none. Values are reported as estimate (Monte Carlo standard error), except for the missingness columns, which are reported without Monte Carlo standard error.

Estimand	Estimator	Method	Bias	Var	RMSE	MAE	Coverage	Var. ratio	Missingness Point	Missingness SE
ATE	WLS	IPTW	0.030 (0.001)	0.010 ($< 10^{-3}$)	0.104 (0.001)	0.080 ($< 10^{-3}$)	0.814 (0.005)	0.534 (0.014)	0.000	0.000
		CBPS-JI	0.018 (0.001)	0.006 ($< 10^{-3}$)	0.081 ($< 10^{-3}$)	0.064 ($< 10^{-3}$)	0.781 (0.006)	0.440 (0.009)	0.000	0.000
		CBPS-OI	0.045 (0.001)	0.005 ($< 10^{-3}$)	0.085 ($< 10^{-3}$)	0.069 ($< 10^{-3}$)	0.909 (0.004)	1.289 (0.026)	0.002	0.002
		CBPS-TLF	0.082 ($< 10^{-3}$)	0.002 ($< 10^{-3}$)	0.095 ($< 10^{-3}$)	0.084 ($< 10^{-3}$)	0.331 (0.007)	0.413 (0.008)	0.000	0.000
		EB	0.016 ($< 10^{-3}$)	0.003 ($< 10^{-3}$)	0.057 ($< 10^{-3}$)	0.046 ($< 10^{-3}$)	0.990 (0.001)	1.895 (0.038)	0.000	0.000
		KOM	0.023 ($< 10^{-3}$)	0.003 ($< 10^{-3}$)	0.058 ($< 10^{-3}$)	0.047 ($< 10^{-3}$)	0.976 (0.002)	1.672 (0.034)	0.000	0.000
	DR	IPTW	0.001 (0.001)	0.007 ($< 10^{-3}$)	0.081 (0.002)	0.057 ($< 10^{-3}$)	0.873 (0.005)	0.745 (0.022)	0.001	0.001
		CBPS-JI	0.002 (0.001)	0.005 ($< 10^{-3}$)	0.073 ($< 10^{-3}$)	0.057 ($< 10^{-3}$)	0.799 (0.006)	0.444 (0.010)	0.000	0.000
		CBPS-OI	0.002 ($< 10^{-3}$)	0.003 ($< 10^{-3}$)	0.055 ($< 10^{-3}$)	0.044 ($< 10^{-3}$)	0.893 (0.004)	0.751 (0.015)	0.002	0.002
		CBPS-TLF	0.002 ($< 10^{-3}$)	0.003 ($< 10^{-3}$)	0.055 ($< 10^{-3}$)	0.044 ($< 10^{-3}$)	0.837 (0.005)	0.546 (0.011)	0.000	0.000
		EB	0.002 ($< 10^{-3}$)	0.003 ($< 10^{-3}$)	0.055 ($< 10^{-3}$)	0.044 ($< 10^{-3}$)	0.893 (0.004)	0.751 (0.015)	0.000	0.000
		KOM	0.002 ($< 10^{-3}$)	0.003 ($< 10^{-3}$)	0.055 ($< 10^{-3}$)	0.044 ($< 10^{-3}$)	0.893 (0.004)	0.751 (0.015)	0.000	0.000
ATT	WLS	IPTW	0.041 (0.002)	0.020 ($< 10^{-3}$)	0.147 (0.002)	0.118 (0.001)	0.726 (0.006)	0.495 (0.012)	0.000	0.000
		CBPS-JI	0.023 (0.002)	0.016 ($< 10^{-3}$)	0.127 (0.001)	0.101 (0.001)	0.942 (0.005)	16.971 (9.104)	0.000	0.566
		CBPS-OI	0.034 (0.002)	0.015 ($< 10^{-3}$)	0.128 (0.001)	0.102 (0.001)	0.880 (0.005)	1.406 (0.032)	0.002	0.002
		CBPS-TLF	0.092 ($< 10^{-3}$)	0.003 ($< 10^{-3}$)	0.107 ($< 10^{-3}$)	0.094 ($< 10^{-3}$)	0.526 (0.007)	0.825 (0.017)	0.000	0.000
		EB	0.042 ($< 10^{-3}$)	0.005 ($< 10^{-3}$)	0.082 ($< 10^{-3}$)	0.066 ($< 10^{-3}$)	0.951 (0.003)	1.745 (0.035)	0.000	0.000
		KOM	0.035 ($< 10^{-3}$)	0.005 ($< 10^{-3}$)	0.076 ($< 10^{-3}$)	0.061 ($< 10^{-3}$)	0.951 (0.003)	1.547 (0.031)	0.000	0.000
	DR	IPTW	-0.002 (0.002)	0.013 ($< 10^{-3}$)	0.113 (0.002)	0.082 (0.001)	0.848 (0.005)	0.703 (0.019)	0.002	0.002
		CBPS-JI	-0.002 (0.002)	0.014 ($< 10^{-3}$)	0.118 (0.001)	0.092 (0.001)	0.898 (0.007)	7.356 (4.813)	0.000	0.582
		CBPS-OI	-0.002 (0.002)	0.011 ($< 10^{-3}$)	0.107 (0.001)	0.083 ($< 10^{-3}$)	0.858 (0.005)	0.759 (0.046)	0.003	0.003
		CBPS-TLF	-0.002 (0.001)	0.006 ($< 10^{-3}$)	0.078 ($< 10^{-3}$)	0.063 ($< 10^{-3}$)	0.867 (0.005)	0.679 (0.014)	0.000	0.000
		EB	$< 10^{-3}$ (0.001)	0.007 ($< 10^{-3}$)	0.082 ($< 10^{-3}$)	0.066 ($< 10^{-3}$)	0.864 (0.005)	0.729 (0.015)	0.000	0.000
		KOM	-0.002 (0.001)	0.006 ($< 10^{-3}$)	0.080 ($< 10^{-3}$)	0.064 ($< 10^{-3}$)	0.868 (0.005)	0.728 (0.015)	0.000	0.000

Table 10: Scenario: $n = 250$, proportion treated: high; complexity: low; treatment-effect level: none. Values are reported as estimate (Monte Carlo standard error), except for the missingness columns, which are reported without Monte Carlo standard error.

Estimand	Estimator	Method	Bias	Var	RMSE	MAE	Coverage	Var. ratio	Missingness	
									Point	SE
ATE	WLS	IPTW	0.006 (0.001)	0.006 ($< 10^{-3}$)	0.079 ($< 10^{-3}$)	0.063 ($< 10^{-3}$)	0.889 (0.004)	0.782 (0.016)	0.000	0.000
		CBPS-JI	0.005 (0.001)	0.005 ($< 10^{-3}$)	0.073 ($< 10^{-3}$)	0.058 ($< 10^{-3}$)	0.874 (0.005)	0.664 (0.014)	0.000	0.000
		CBPS-OI	0.013 ($< 10^{-3}$)	0.005 ($< 10^{-3}$)	0.071 ($< 10^{-3}$)	0.057 ($< 10^{-3}$)	0.933 (0.004)	1.095 (0.022)	0.000	0.000
		CBPS-TLF	0.024 ($< 10^{-3}$)	0.004 ($< 10^{-3}$)	0.067 ($< 10^{-3}$)	0.054 ($< 10^{-3}$)	0.705 (0.006)	0.346 (0.007)	0.000	0.000
		EB	0.006 ($< 10^{-3}$)	0.005 ($< 10^{-3}$)	0.070 ($< 10^{-3}$)	0.056 ($< 10^{-3}$)	0.950 (0.003)	1.146 (0.023)	0.000	0.000
		KOM	0.021 ($< 10^{-3}$)	0.004 ($< 10^{-3}$)	0.067 ($< 10^{-3}$)	0.054 ($< 10^{-3}$)	0.923 (0.004)	1.039 (0.021)	0.000	0.000
	DR	IPTW	-0.005 (0.001)	0.006 ($< 10^{-3}$)	0.077 ($< 10^{-3}$)	0.061 ($< 10^{-3}$)	0.865 (0.005)	0.699 (0.015)	0.000	0.001
		CBPS-JI	-0.005 (0.001)	0.006 ($< 10^{-3}$)	0.076 ($< 10^{-3}$)	0.060 ($< 10^{-3}$)	0.844 (0.005)	0.608 (0.013)	0.000	0.001
		CBPS-OI	-0.005 (0.001)	0.005 ($< 10^{-3}$)	0.072 ($< 10^{-3}$)	0.058 ($< 10^{-3}$)	0.884 (0.005)	0.756 (0.016)	0.001	0.001
		CBPS-TLF	-0.005 (0.001)	0.005 ($< 10^{-3}$)	0.073 ($< 10^{-3}$)	0.058 ($< 10^{-3}$)	0.762 (0.006)	0.399 (0.008)	0.000	0.001
		EB	-0.005 (0.001)	0.005 ($< 10^{-3}$)	0.073 ($< 10^{-3}$)	0.058 ($< 10^{-3}$)	0.884 (0.005)	0.794 (0.042)	0.002	0.002
		KOM	-0.005 (0.001)	0.005 ($< 10^{-3}$)	0.072 ($< 10^{-3}$)	0.058 ($< 10^{-3}$)	0.884 (0.005)	0.756 (0.016)	0.001	0.001
ATT	WLS	IPTW	0.008 (0.001)	0.008 ($< 10^{-3}$)	0.091 ($< 10^{-3}$)	0.073 ($< 10^{-3}$)	0.873 (0.005)	0.759 (0.016)	0.000	0.000
		CBPS-JI	0.006 (0.001)	0.008 ($< 10^{-3}$)	0.091 ($< 10^{-3}$)	0.072 ($< 10^{-3}$)	0.863 (0.005)	0.889 (0.033)	0.000	0.016
		CBPS-OI	0.014 (0.001)	0.007 ($< 10^{-3}$)	0.082 ($< 10^{-3}$)	0.066 ($< 10^{-3}$)	0.915 (0.004)	1.052 (0.022)	0.000	0.000
		CBPS-TLF	0.027 ($< 10^{-3}$)	0.004 ($< 10^{-3}$)	0.071 ($< 10^{-3}$)	0.057 ($< 10^{-3}$)	0.828 (0.005)	0.632 (0.013)	0.000	0.000
		EB	0.013 (0.001)	0.006 ($< 10^{-3}$)	0.077 ($< 10^{-3}$)	0.062 ($< 10^{-3}$)	0.938 (0.003)	1.124 (0.023)	0.000	0.000
		KOM	0.025 ($< 10^{-3}$)	0.005 ($< 10^{-3}$)	0.073 ($< 10^{-3}$)	0.059 ($< 10^{-3}$)	0.905 (0.004)	0.984 (0.020)	0.000	0.000
	DR	IPTW	-0.007 (0.001)	0.008 ($< 10^{-3}$)	0.090 ($< 10^{-3}$)	0.071 ($< 10^{-3}$)	0.850 (0.005)	0.675 (0.014)	0.000	0.001
		CBPS-JI	-0.006 (0.001)	0.008 ($< 10^{-3}$)	0.091 ($< 10^{-3}$)	0.072 ($< 10^{-3}$)	0.824 (0.005)	0.846 (0.103)	0.000	0.019
		CBPS-OI	-0.007 (0.001)	0.007 ($< 10^{-3}$)	0.086 ($< 10^{-3}$)	0.068 ($< 10^{-3}$)	0.862 (0.005)	0.721 (0.016)	0.001	0.001
		CBPS-TLF	-0.006 (0.001)	0.007 ($< 10^{-3}$)	0.083 ($< 10^{-3}$)	0.066 ($< 10^{-3}$)	0.836 (0.005)	0.569 (0.012)	0.000	0.001
		EB	-0.005 (0.001)	0.007 ($< 10^{-3}$)	0.085 ($< 10^{-3}$)	0.068 ($< 10^{-3}$)	0.869 (0.005)	0.735 (0.016)	0.001	0.001
		KOM	-0.006 (0.001)	0.007 ($< 10^{-3}$)	0.083 ($< 10^{-3}$)	0.066 ($< 10^{-3}$)	0.872 (0.005)	0.738 (0.016)	0.001	0.001

Table 11: Scenario: $n = 250$, proportion treated: high; complexity: moderate; treatment-effect level: none. Values are reported as estimate (Monte Carlo standard error), except for the missingness columns, which are reported without Monte Carlo standard error.

Estimand	Estimator	Method	Bias	Var	RMSE	MAE	Coverage	Var. ratio	Missingness	
									Point	SE
ATE	WLS	IPTW	0.015 (0.001)	0.011 ($< 10^{-3}$)	0.104 (0.001)	0.083 ($< 10^{-3}$)	0.812 (0.006)	0.622 (0.014)	0.000	0.000
		CBPS-JI	0.008 (0.001)	0.007 ($< 10^{-3}$)	0.081 ($< 10^{-3}$)	0.065 ($< 10^{-3}$)	0.809 (0.006)	0.489 (0.010)	0.000	0.000
		CBPS-OI	0.026 (0.001)	0.006 ($< 10^{-3}$)	0.082 ($< 10^{-3}$)	0.067 ($< 10^{-3}$)	0.897 (0.004)	1.178 (0.024)	0.000	0.000
		CBPS-TLF	0.048 ($< 10^{-3}$)	0.004 ($< 10^{-3}$)	0.078 ($< 10^{-3}$)	0.064 ($< 10^{-3}$)	0.563 (0.007)	0.289 (0.006)	0.000	0.000
		EB	0.012 ($< 10^{-3}$)	0.005 ($< 10^{-3}$)	0.070 ($< 10^{-3}$)	0.056 ($< 10^{-3}$)	0.959 (0.003)	1.417 (0.030)	0.000	0.000
		KOM	0.031 ($< 10^{-3}$)	0.004 ($< 10^{-3}$)	0.073 ($< 10^{-3}$)	0.059 ($< 10^{-3}$)	0.904 (0.004)	1.152 (0.023)	0.000	0.000
	DR	IPTW	-0.005 (0.001)	0.009 ($< 10^{-3}$)	0.093 (0.001)	0.073 ($< 10^{-3}$)	0.783 (0.006)	0.560 (0.018)	0.000	0.005
		CBPS-JI	-0.004 (0.001)	0.008 ($< 10^{-3}$)	0.088 ($< 10^{-3}$)	0.070 ($< 10^{-3}$)	0.746 (0.006)	0.397 (0.009)	0.000	0.009
		CBPS-OI	-0.005 (0.001)	0.007 ($< 10^{-3}$)	0.081 ($< 10^{-3}$)	0.064 ($< 10^{-3}$)	0.791 (0.006)	0.552 (0.012)	0.006	0.006
		CBPS-TLF	-0.005 (0.001)	0.007 ($< 10^{-3}$)	0.081 ($< 10^{-3}$)	0.065 ($< 10^{-3}$)	0.701 (0.006)	0.331 (0.007)	0.000	0.006
		EB	-0.005 (0.001)	0.007 ($< 10^{-3}$)	0.081 ($< 10^{-3}$)	0.064 ($< 10^{-3}$)	0.792 (0.006)	0.552 (0.012)	0.005	0.005
		KOM	-0.005 (0.001)	0.007 ($< 10^{-3}$)	0.081 ($< 10^{-3}$)	0.064 ($< 10^{-3}$)	0.792 (0.006)	0.552 (0.012)	0.005	0.005
ATT	WLS	IPTW	0.019 (0.002)	0.016 ($< 10^{-3}$)	0.127 (0.001)	0.102 (0.001)	0.776 (0.006)	0.587 (0.013)	0.000	0.000
		CBPS-JI	0.012 (0.002)	0.015 ($< 10^{-3}$)	0.125 (0.001)	0.100 (0.001)	0.876 (0.006)	2.304 (0.120)	0.000	0.341
		CBPS-OI	0.022 (0.002)	0.013 ($< 10^{-3}$)	0.118 (0.001)	0.094 (0.001)	0.869 (0.005)	1.127 (0.026)	0.000	0.000
		CBPS-TLF	0.052 ($< 10^{-3}$)	0.004 ($< 10^{-3}$)	0.083 ($< 10^{-3}$)	0.068 ($< 10^{-3}$)	0.694 (0.007)	0.524 (0.011)	0.000	0.000
		EB	0.027 (0.001)	0.006 ($< 10^{-3}$)	0.085 ($< 10^{-3}$)	0.068 ($< 10^{-3}$)	0.931 (0.004)	1.312 (0.027)	0.000	0.000
		KOM	0.040 (0.001)	0.005 ($< 10^{-3}$)	0.084 ($< 10^{-3}$)	0.068 ($< 10^{-3}$)	0.873 (0.005)	1.077 (0.022)	0.000	0.000
	DR	IPTW	-0.007 (0.002)	0.013 ($< 10^{-3}$)	0.116 (0.002)	0.090 (0.001)	0.759 (0.006)	0.546 (0.019)	0.000	0.005
		CBPS-JI	-0.005 (0.002)	0.014 ($< 10^{-3}$)	0.119 (0.001)	0.094 (0.001)	0.806 (0.007)	1.339 (0.102)	0.000	0.392
		CBPS-OI	-0.006 (0.002)	0.012 ($< 10^{-3}$)	0.111 (0.001)	0.088 ($< 10^{-3}$)	0.763 (0.006)	0.531 (0.013)	0.006	0.006
		CBPS-TLF	-0.006 (0.001)	0.010 ($< 10^{-3}$)	0.099 ($< 10^{-3}$)	0.079 ($< 10^{-3}$)	0.746 (0.006)	0.410 (0.009)	0.000	0.006
		EB	-0.005 (0.001)	0.010 ($< 10^{-3}$)	0.101 (0.001)	0.080 ($< 10^{-3}$)	0.767 (0.006)	0.522 (0.012)	0.005	0.005
		KOM	-0.006 (0.001)	0.010 ($< 10^{-3}$)	0.099 (0.001)	0.079 ($< 10^{-3}$)	0.768 (0.006)	0.526 (0.012)	0.005	0.005

Table 12: Scenario: $n = 250$, proportion treated: high; complexity: high; treatment-effect level: none. Values are reported as estimate (Monte Carlo standard error), except for the missingness columns, which are reported without Monte Carlo standard error.

Estimand	Estimator	Method	Bias	Var	RMSE	MAE	Coverage	Var. ratio	Missingness Point	Missingness SE
ATE	WLS	IPTW	0.036 (0.002)	0.022 ($< 10^{-3}$)	0.153 (0.002)	0.122 (0.001)	0.643 (0.007)	0.390 (0.011)	0.000	0.000
		CBPS-JI	0.013 (0.001)	0.011 ($< 10^{-3}$)	0.103 ($< 10^{-3}$)	0.084 ($< 10^{-3}$)	0.646 (0.007)	0.266 (0.006)	0.000	0.000
		CBPS-OI	0.047 (0.002)	0.012 ($< 10^{-3}$)	0.118 ($< 10^{-3}$)	0.099 ($< 10^{-3}$)	0.763 (0.006)	0.946 (0.019)	0.000	0.000
		CBPS-TLF	0.084 ($< 10^{-3}$)	0.004 ($< 10^{-3}$)	0.103 ($< 10^{-3}$)	0.088 ($< 10^{-3}$)	0.332 (0.007)	0.229 (0.004)	0.000	0.000
		EB	0.023 ($< 10^{-3}$)	0.005 ($< 10^{-3}$)	0.072 ($< 10^{-3}$)	0.058 ($< 10^{-3}$)	0.952 (0.003)	1.742 (0.036)	0.000	0.000
		KOM	0.048 (0.001)	0.005 ($< 10^{-3}$)	0.086 ($< 10^{-3}$)	0.070 ($< 10^{-3}$)	0.859 (0.005)	1.240 (0.025)	0.000	0.000
	DR	IPTW	-0.011 (0.002)	0.014 ($< 10^{-3}$)	0.120 (0.002)	0.090 (0.001)	0.579 (0.007)	0.545 (0.043)	0.001	0.021
		CBPS-JI	-0.009 (0.002)	0.013 ($< 10^{-3}$)	0.115 (0.001)	0.091 ($< 10^{-3}$)	0.564 (0.007)	0.206 (0.005)	0.000	0.053
		CBPS-OI	-0.012 (0.001)	0.010 ($< 10^{-3}$)	0.102 (0.001)	0.081 ($< 10^{-3}$)	0.559 (0.007)	0.227 (0.006)	0.020	0.020
		CBPS-TLF	-0.011 (0.001)	0.010 ($< 10^{-3}$)	0.102 (0.001)	0.081 ($< 10^{-3}$)	0.521 (0.007)	0.171 (0.004)	0.000	0.020
		EB	-0.012 (0.001)	0.010 ($< 10^{-3}$)	0.102 (0.001)	0.081 ($< 10^{-3}$)	0.560 (0.007)	0.227 (0.006)	0.020	0.020
		KOM	-0.012 (0.001)	0.010 ($< 10^{-3}$)	0.102 (0.001)	0.081 ($< 10^{-3}$)	0.559 (0.007)	0.227 (0.006)	0.020	0.020
ATT	WLS	IPTW	0.043 (0.003)	0.033 ($< 10^{-3}$)	0.188 (0.002)	0.154 (0.002)	0.604 (0.007)	0.369 (0.010)	0.000	0.000
		CBPS-JI	0.044 (0.002)	0.022 ($< 10^{-3}$)	0.156 (0.001)	0.129 (0.001)	0.837 (0.022)	2813.006 (2368.570)	0.000	0.945
		CBPS-OI	0.047 (0.002)	0.023 ($< 10^{-3}$)	0.157 (0.001)	0.130 (0.001)	0.787 (0.006)	1.207 (0.024)	0.000	0.000
		CBPS-TLF	0.086 ($< 10^{-3}$)	0.004 ($< 10^{-3}$)	0.108 ($< 10^{-3}$)	0.092 ($< 10^{-3}$)	0.457 (0.007)	0.408 (0.008)	0.000	0.000
		EB	0.054 (0.001)	0.007 ($< 10^{-3}$)	0.101 ($< 10^{-3}$)	0.082 ($< 10^{-3}$)	0.887 (0.004)	1.449 (0.031)	0.000	0.000
		KOM	0.061 (0.001)	0.007 ($< 10^{-3}$)	0.103 ($< 10^{-3}$)	0.085 ($< 10^{-3}$)	0.825 (0.005)	1.147 (0.024)	0.000	0.000
	DR	IPTW	-0.015 (0.002)	0.022 ($< 10^{-3}$)	0.148 (0.002)	0.113 (0.001)	0.535 (0.007)	0.500 (0.045)	0.002	0.021
		CBPS-JI	-0.013 (0.002)	0.020 ($< 10^{-3}$)	0.142 (0.001)	0.114 (0.001)	0.738 (0.049)	3.124 (1.042)	0.000	0.984
		CBPS-OI	-0.014 (0.002)	0.019 ($< 10^{-3}$)	0.138 (0.001)	0.110 (0.001)	0.527 (0.007)	0.235 (0.006)	0.019	0.019
		CBPS-TLF	-0.016 (0.002)	0.017 ($< 10^{-3}$)	0.131 (0.001)	0.104 (0.001)	0.517 (0.007)	0.182 (0.005)	0.000	0.021
		EB	-0.014 (0.002)	0.017 ($< 10^{-3}$)	0.132 (0.001)	0.105 (0.001)	0.528 (0.007)	0.211 (0.006)	0.019	0.019
		KOM	-0.016 (0.002)	0.017 ($< 10^{-3}$)	0.131 (0.001)	0.104 (0.001)	0.525 (0.007)	0.210 (0.006)	0.019	0.019

Table 13: Scenario: $n = 500$, proportion treated: low; complexity: low; treatment-effect level: none. Values are reported as estimate (Monte Carlo standard error), except for the missingness columns, which are reported without Monte Carlo standard error.

Estimand	Estimator	Method	Bias	Var	RMSE	MAE	Coverage	Var. ratio	Missingness Point	Missingness SE
ATE	WLS	IPTW	0.002 ($< 10^{-3}$)	0.002 ($< 10^{-3}$)	0.049 ($< 10^{-3}$)	0.039 ($< 10^{-3}$)	0.931 (0.004)	0.881 (0.017)	0.000	0.000
		CBPS-JI	0.005 ($< 10^{-3}$)	0.002 ($< 10^{-3}$)	0.048 ($< 10^{-3}$)	0.039 ($< 10^{-3}$)	0.919 (0.004)	0.821 (0.016)	0.000	0.000
		CBPS-OI	0.010 ($< 10^{-3}$)	0.002 ($< 10^{-3}$)	0.049 ($< 10^{-3}$)	0.039 ($< 10^{-3}$)	0.956 (0.003)	1.110 (0.022)	0.000	0.000
		CBPS-TLF	0.025 ($< 10^{-3}$)	0.002 ($< 10^{-3}$)	0.050 ($< 10^{-3}$)	0.041 ($< 10^{-3}$)	0.749 (0.006)	0.464 (0.009)	0.000	0.000
		EB	0.003 ($< 10^{-3}$)	0.002 ($< 10^{-3}$)	0.049 ($< 10^{-3}$)	0.039 ($< 10^{-3}$)	0.967 (0.003)	1.209 (0.024)	0.000	0.000
		KOM	0.011 ($< 10^{-3}$)	0.002 ($< 10^{-3}$)	0.046 ($< 10^{-3}$)	0.036 ($< 10^{-3}$)	0.961 (0.003)	1.151 (0.023)	0.000	0.000
	DR	IPTW	0.001 ($< 10^{-3}$)	0.002 ($< 10^{-3}$)	0.049 ($< 10^{-3}$)	0.039 ($< 10^{-3}$)	0.922 (0.004)	0.866 (0.017)	0.000	0.000
		CBPS-JI	$< 10^{-3}$ ($< 10^{-3}$)	0.002 ($< 10^{-3}$)	0.048 ($< 10^{-3}$)	0.039 ($< 10^{-3}$)	0.912 (0.004)	0.807 (0.016)	0.000	0.000
		CBPS-OI	$< 10^{-3}$ ($< 10^{-3}$)	0.002 ($< 10^{-3}$)	0.046 ($< 10^{-3}$)	0.037 ($< 10^{-3}$)	0.932 (0.004)	0.918 (0.018)	0.000	0.000
		CBPS-TLF	$< 10^{-3}$ ($< 10^{-3}$)	0.002 ($< 10^{-3}$)	0.046 ($< 10^{-3}$)	0.037 ($< 10^{-3}$)	0.828 (0.005)	0.498 (0.010)	0.000	0.000
		EB	$< 10^{-3}$ ($< 10^{-3}$)	0.002 ($< 10^{-3}$)	0.046 ($< 10^{-3}$)	0.037 ($< 10^{-3}$)	0.932 (0.004)	0.918 (0.018)	0.000	0.000
		KOM	$< 10^{-3}$ ($< 10^{-3}$)	0.002 ($< 10^{-3}$)	0.046 ($< 10^{-3}$)	0.037 ($< 10^{-3}$)	0.932 (0.004)	0.918 (0.018)	0.000	0.000
ATT	WLS	IPTW	0.010 ($< 10^{-3}$)	0.002 ($< 10^{-3}$)	0.051 ($< 10^{-3}$)	0.041 ($< 10^{-3}$)	0.943 (0.003)	0.984 (0.020)	0.000	0.000
		CBPS-JI	0.006 ($< 10^{-3}$)	0.002 ($< 10^{-3}$)	0.049 ($< 10^{-3}$)	0.040 ($< 10^{-3}$)	0.959 (0.003)	1.140 (0.023)	0.000	0.000
		CBPS-OI	0.016 ($< 10^{-3}$)	0.002 ($< 10^{-3}$)	0.052 ($< 10^{-3}$)	0.042 ($< 10^{-3}$)	0.954 (0.003)	1.123 (0.022)	0.000	0.000
		CBPS-TLF	0.019 ($< 10^{-3}$)	0.002 ($< 10^{-3}$)	0.050 ($< 10^{-3}$)	0.040 ($< 10^{-3}$)	0.952 (0.003)	1.175 (0.023)	0.000	0.000
		EB	0.006 ($< 10^{-3}$)	0.003 ($< 10^{-3}$)	0.051 ($< 10^{-3}$)	0.041 ($< 10^{-3}$)	0.972 (0.002)	1.272 (0.025)	0.000	0.000
		KOM	0.010 ($< 10^{-3}$)	0.002 ($< 10^{-3}$)	0.048 ($< 10^{-3}$)	0.038 ($< 10^{-3}$)	0.962 (0.003)	1.186 (0.024)	0.000	0.000
	DR	IPTW	$< 10^{-3}$ ($< 10^{-3}$)	0.002 ($< 10^{-3}$)	0.049 ($< 10^{-3}$)	0.039 ($< 10^{-3}$)	0.946 (0.003)	0.976 (0.019)	0.000	0.000
		CBPS-JI	$< 10^{-3}$ ($< 10^{-3}$)	0.002 ($< 10^{-3}$)	0.049 ($< 10^{-3}$)	0.039 ($< 10^{-3}$)	0.948 (0.003)	0.996 (0.020)	0.000	0.000
		CBPS-OI	$< 10^{-3}$ ($< 10^{-3}$)	0.002 ($< 10^{-3}$)	0.048 ($< 10^{-3}$)	0.039 ($< 10^{-3}$)	0.948 (0.003)	0.990 (0.019)	0.000	0.000
		CBPS-TLF	0.002 ($< 10^{-3}$)	0.002 ($< 10^{-3}$)	0.048 ($< 10^{-3}$)	0.038 ($< 10^{-3}$)	0.965 (0.003)	1.161 (0.023)	0.000	0.000
		EB	0.001 ($< 10^{-3}$)	0.003 ($< 10^{-3}$)	0.051 ($< 10^{-3}$)	0.041 ($< 10^{-3}$)	0.955 (0.003)	1.031 (0.020)	0.000	0.000
		KOM	$< 10^{-3}$ ($< 10^{-3}$)	0.002 ($< 10^{-3}$)	0.047 ($< 10^{-3}$)	0.038 ($< 10^{-3}$)	0.952 (0.003)	1.002 (0.020)	0.000	0.000

Table 14: Scenario: $n = 500$, proportion treated: low; complexity: moderate; treatment-effect level: none. Values are reported as estimate (Monte Carlo standard error), except for the missingness columns, which are reported without Monte Carlo standard error.

Estimand	Estimator	Method	Bias	Var	RMSE	MAE	Coverage	Var. ratio	Missingness Point	Missingness SE
ATE	WLS	IPTW	0.008 ($< 10^{-3}$)	0.003 ($< 10^{-3}$)	0.058 ($< 10^{-3}$)	0.045 ($< 10^{-3}$)	0.911 (0.004)	0.802 (0.017)	0.000	0.000
		CBPS-JI	0.014 ($< 10^{-3}$)	0.003 ($< 10^{-3}$)	0.056 ($< 10^{-3}$)	0.044 ($< 10^{-3}$)	0.872 (0.005)	0.653 (0.013)	0.000	0.000
		CBPS-OI	0.025 ($< 10^{-3}$)	0.003 ($< 10^{-3}$)	0.057 ($< 10^{-3}$)	0.045 ($< 10^{-3}$)	0.966 (0.003)	1.253 (0.025)	0.000	0.000
		CBPS-TLF	0.054 ($< 10^{-3}$)	0.002 ($< 10^{-3}$)	0.068 ($< 10^{-3}$)	0.057 ($< 10^{-3}$)	0.486 (0.007)	0.395 (0.008)	0.000	0.000
		EB	0.009 ($< 10^{-3}$)	0.002 ($< 10^{-3}$)	0.051 ($< 10^{-3}$)	0.040 ($< 10^{-3}$)	0.982 (0.002)	1.457 (0.029)	0.000	0.000
		KOM	0.016 ($< 10^{-3}$)	0.002 ($< 10^{-3}$)	0.048 ($< 10^{-3}$)	0.038 ($< 10^{-3}$)	0.979 (0.002)	1.392 (0.027)	0.000	0.000
	DR	IPTW	0.003 ($< 10^{-3}$)	0.003 ($< 10^{-3}$)	0.054 ($< 10^{-3}$)	0.042 ($< 10^{-3}$)	0.899 (0.004)	0.792 (0.017)	0.000	0.000
		CBPS-JI	0.003 ($< 10^{-3}$)	0.003 ($< 10^{-3}$)	0.054 ($< 10^{-3}$)	0.042 ($< 10^{-3}$)	0.864 (0.005)	0.645 (0.013)	0.000	0.000
		CBPS-OI	0.003 ($< 10^{-3}$)	0.002 ($< 10^{-3}$)	0.047 ($< 10^{-3}$)	0.038 ($< 10^{-3}$)	0.923 (0.004)	0.868 (0.017)	0.000	0.000
		CBPS-TLF	0.004 ($< 10^{-3}$)	0.002 ($< 10^{-3}$)	0.047 ($< 10^{-3}$)	0.038 ($< 10^{-3}$)	0.811 (0.006)	0.465 (0.009)	0.000	0.000
		EB	0.003 ($< 10^{-3}$)	0.002 ($< 10^{-3}$)	0.047 ($< 10^{-3}$)	0.038 ($< 10^{-3}$)	0.923 (0.004)	0.868 (0.017)	0.000	0.000
		KOM	0.003 ($< 10^{-3}$)	0.002 ($< 10^{-3}$)	0.047 ($< 10^{-3}$)	0.038 ($< 10^{-3}$)	0.923 (0.004)	0.868 (0.017)	0.000	0.000
ATT	WLS	IPTW	0.029 ($< 10^{-3}$)	0.003 ($< 10^{-3}$)	0.066 ($< 10^{-3}$)	0.053 ($< 10^{-3}$)	0.890 (0.004)	0.932 (0.020)	0.000	0.000
		CBPS-JI	0.016 ($< 10^{-3}$)	0.003 ($< 10^{-3}$)	0.056 ($< 10^{-3}$)	0.045 ($< 10^{-3}$)	0.974 (0.002)	1.518 (0.031)	0.000	0.000
		CBPS-OI	0.038 ($< 10^{-3}$)	0.003 ($< 10^{-3}$)	0.066 ($< 10^{-3}$)	0.054 ($< 10^{-3}$)	0.922 (0.004)	1.275 (0.026)	0.000	0.000
		CBPS-TLF	0.040 ($< 10^{-3}$)	0.002 ($< 10^{-3}$)	0.061 ($< 10^{-3}$)	0.050 ($< 10^{-3}$)	0.872 (0.005)	1.057 (0.021)	0.000	0.000
		EB	0.014 ($< 10^{-3}$)	0.003 ($< 10^{-3}$)	0.054 ($< 10^{-3}$)	0.043 ($< 10^{-3}$)	0.980 (0.002)	1.563 (0.032)	0.000	0.000
		KOM	0.016 ($< 10^{-3}$)	0.002 ($< 10^{-3}$)	0.051 ($< 10^{-3}$)	0.041 ($< 10^{-3}$)	0.976 (0.002)	1.470 (0.029)	0.000	0.000
	DR	IPTW	0.001 ($< 10^{-3}$)	0.003 ($< 10^{-3}$)	0.053 ($< 10^{-3}$)	0.042 ($< 10^{-3}$)	0.940 (0.003)	0.953 (0.020)	0.000	0.000
		CBPS-JI	0.002 ($< 10^{-3}$)	0.003 ($< 10^{-3}$)	0.052 ($< 10^{-3}$)	0.042 ($< 10^{-3}$)	0.947 (0.003)	1.016 (0.021)	0.000	0.000
		CBPS-OI	0.002 ($< 10^{-3}$)	0.003 ($< 10^{-3}$)	0.050 ($< 10^{-3}$)	0.040 ($< 10^{-3}$)	0.946 (0.003)	1.001 (0.020)	0.000	0.000
		CBPS-TLF	0.004 ($< 10^{-3}$)	0.002 ($< 10^{-3}$)	0.049 ($< 10^{-3}$)	0.039 ($< 10^{-3}$)	0.952 (0.003)	1.049 (0.021)	0.000	0.000
		EB	0.003 ($< 10^{-3}$)	0.003 ($< 10^{-3}$)	0.052 ($< 10^{-3}$)	0.042 ($< 10^{-3}$)	0.949 (0.003)	1.011 (0.020)	0.000	0.000
		KOM	0.002 ($< 10^{-3}$)	0.002 ($< 10^{-3}$)	0.049 ($< 10^{-3}$)	0.039 ($< 10^{-3}$)	0.949 (0.003)	0.993 (0.020)	0.000	0.000

Table 15: Scenario: $n = 500$, proportion treated: low; complexity: high; treatment-effect level: none. Values are reported as estimate (Monte Carlo standard error), except for the missingness columns, which are reported without Monte Carlo standard error.

Estimand	Estimator	Method	Bias	Var	RMSE	MAE	Coverage	Var. ratio	Missingness	
									Point	SE
ATE	WLS	IPTW	0.015 (0.001)	0.007 ($< 10^{-3}$)	0.083 (0.001)	0.063 ($< 10^{-3}$)	0.887 (0.004)	0.622 (0.016)	0.000	0.000
		CBPS-JI	0.030 ($< 10^{-3}$)	0.005 ($< 10^{-3}$)	0.075 ($< 10^{-3}$)	0.060 ($< 10^{-3}$)	0.748 (0.006)	0.433 (0.009)	0.000	0.000
		CBPS-OI	0.047 ($< 10^{-3}$)	0.004 ($< 10^{-3}$)	0.078 ($< 10^{-3}$)	0.062 ($< 10^{-3}$)	0.961 (0.003)	1.370 (0.028)	0.000	0.000
		CBPS-TLF	0.091 ($< 10^{-3}$)	0.002 ($< 10^{-3}$)	0.100 ($< 10^{-3}$)	0.091 ($< 10^{-3}$)	0.122 (0.005)	0.327 (0.007)	0.000	0.000
		EB	0.018 ($< 10^{-3}$)	0.002 ($< 10^{-3}$)	0.052 ($< 10^{-3}$)	0.041 ($< 10^{-3}$)	0.994 (0.001)	2.163 (0.044)	0.000	0.000
		KOM	0.022 ($< 10^{-3}$)	0.002 ($< 10^{-3}$)	0.053 ($< 10^{-3}$)	0.042 ($< 10^{-3}$)	0.989 (0.001)	1.917 (0.039)	0.000	0.000
	DR	IPTW	0.003 ($< 10^{-3}$)	0.004 ($< 10^{-3}$)	0.064 (0.002)	0.046 ($< 10^{-3}$)	0.883 (0.005)	0.736 (0.018)	0.000	0.000
		CBPS-JI	0.004 ($< 10^{-3}$)	0.004 ($< 10^{-3}$)	0.065 ($< 10^{-3}$)	0.051 ($< 10^{-3}$)	0.810 (0.006)	0.473 (0.010)	0.000	0.000
		CBPS-OI	0.005 ($< 10^{-3}$)	0.002 ($< 10^{-3}$)	0.048 ($< 10^{-3}$)	0.038 ($< 10^{-3}$)	0.906 (0.004)	0.807 (0.017)	0.000	0.000
		CBPS-TLF	0.006 ($< 10^{-3}$)	0.002 ($< 10^{-3}$)	0.048 ($< 10^{-3}$)	0.038 ($< 10^{-3}$)	0.815 (0.005)	0.490 (0.011)	0.000	0.000
		EB	0.005 ($< 10^{-3}$)	0.002 ($< 10^{-3}$)	0.048 ($< 10^{-3}$)	0.038 ($< 10^{-3}$)	0.907 (0.004)	0.807 (0.017)	0.000	0.000
		KOM	0.005 ($< 10^{-3}$)	0.002 ($< 10^{-3}$)	0.048 ($< 10^{-3}$)	0.038 ($< 10^{-3}$)	0.907 (0.004)	0.807 (0.017)	0.000	0.000
ATT	WLS	IPTW	0.054 (0.001)	0.007 ($< 10^{-3}$)	0.100 ($< 10^{-3}$)	0.082 ($< 10^{-3}$)	0.789 (0.006)	0.765 (0.017)	0.000	0.000
		CBPS-JI	0.023 (0.001)	0.005 ($< 10^{-3}$)	0.075 ($< 10^{-3}$)	0.060 ($< 10^{-3}$)	0.980 (0.002)	3.686 (0.362)	0.000	0.004
		CBPS-OI	0.062 ($< 10^{-3}$)	0.005 ($< 10^{-3}$)	0.092 ($< 10^{-3}$)	0.076 ($< 10^{-3}$)	0.863 (0.005)	1.350 (0.027)	0.000	0.000
		CBPS-TLF	0.059 ($< 10^{-3}$)	0.002 ($< 10^{-3}$)	0.075 ($< 10^{-3}$)	0.063 ($< 10^{-3}$)	0.727 (0.006)	0.987 (0.020)	0.000	0.000
		EB	0.030 ($< 10^{-3}$)	0.003 ($< 10^{-3}$)	0.061 ($< 10^{-3}$)	0.049 ($< 10^{-3}$)	0.983 (0.002)	2.076 (0.042)	0.000	0.000
		KOM	0.019 ($< 10^{-3}$)	0.003 ($< 10^{-3}$)	0.054 ($< 10^{-3}$)	0.044 ($< 10^{-3}$)	0.990 (0.001)	2.043 (0.041)	0.000	0.000
	DR	IPTW	-0.004 ($< 10^{-3}$)	0.005 ($< 10^{-3}$)	0.070 (0.002)	0.051 ($< 10^{-3}$)	0.933 (0.004)	0.833 (0.017)	0.000	0.000
		CBPS-JI	-0.002 ($< 10^{-3}$)	0.004 ($< 10^{-3}$)	0.065 ($< 10^{-3}$)	0.051 ($< 10^{-3}$)	0.949 (0.003)	1.529 (0.063)	0.000	0.005
		CBPS-OI	-0.004 ($< 10^{-3}$)	0.003 ($< 10^{-3}$)	0.055 ($< 10^{-3}$)	0.043 ($< 10^{-3}$)	0.946 (0.003)	0.976 (0.020)	0.000	0.000
		CBPS-TLF	-0.001 ($< 10^{-3}$)	0.002 ($< 10^{-3}$)	0.048 ($< 10^{-3}$)	0.038 ($< 10^{-3}$)	0.943 (0.003)	0.946 (0.019)	0.000	0.000
		EB	-0.002 ($< 10^{-3}$)	0.003 ($< 10^{-3}$)	0.052 ($< 10^{-3}$)	0.042 ($< 10^{-3}$)	0.951 (0.003)	1.039 (0.021)	0.000	0.000
		KOM	-0.003 ($< 10^{-3}$)	0.003 ($< 10^{-3}$)	0.050 ($< 10^{-3}$)	0.040 ($< 10^{-3}$)	0.959 (0.003)	1.036 (0.021)	0.000	0.000

Table 16: Scenario: $n = 500$, proportion treated: moderate; complexity: low; treatment-effect level: none. Values are reported as estimate (Monte Carlo standard error), except for the missingness columns, which are reported without Monte Carlo standard error.

Estimand	Estimator	Method	Bias	Var	RMSE	MAE	Coverage	Var. ratio	Missingness	
									Point	SE
ATE	WLS	IPTW	0.005 ($< 10^{-3}$)	0.002 ($< 10^{-3}$)	0.040 ($< 10^{-3}$)	0.032 ($< 10^{-3}$)	0.941 (0.003)	0.958 (0.020)	0.000	0.000
		CBPS-JI	0.004 ($< 10^{-3}$)	0.002 ($< 10^{-3}$)	0.039 ($< 10^{-3}$)	0.031 ($< 10^{-3}$)	0.942 (0.003)	0.944 (0.019)	0.000	0.000
		CBPS-OI	0.009 ($< 10^{-3}$)	0.002 ($< 10^{-3}$)	0.040 ($< 10^{-3}$)	0.032 ($< 10^{-3}$)	0.957 (0.003)	1.158 (0.024)	0.000	0.000
		CBPS-TLF	0.007 ($< 10^{-3}$)	0.002 ($< 10^{-3}$)	0.040 ($< 10^{-3}$)	0.032 ($< 10^{-3}$)	0.867 (0.005)	0.608 (0.012)	0.000	0.000
		EB	0.002 ($< 10^{-3}$)	0.002 ($< 10^{-3}$)	0.041 ($< 10^{-3}$)	0.032 ($< 10^{-3}$)	0.968 (0.002)	1.212 (0.025)	0.000	0.000
		KOM	0.008 ($< 10^{-3}$)	0.001 ($< 10^{-3}$)	0.039 ($< 10^{-3}$)	0.031 ($< 10^{-3}$)	0.958 (0.003)	1.165 (0.024)	0.000	0.000
	DR	IPTW	$< 10^{-3}$ ($< 10^{-3}$)	0.002 ($< 10^{-3}$)	0.039 ($< 10^{-3}$)	0.031 ($< 10^{-3}$)	0.943 (0.003)	0.944 (0.019)	0.000	0.000
		CBPS-JI	$< 10^{-3}$ ($< 10^{-3}$)	0.002 ($< 10^{-3}$)	0.039 ($< 10^{-3}$)	0.031 ($< 10^{-3}$)	0.941 (0.003)	0.930 (0.019)	0.000	0.000
		CBPS-OI	$< 10^{-3}$ ($< 10^{-3}$)	0.001 ($< 10^{-3}$)	0.038 ($< 10^{-3}$)	0.030 ($< 10^{-3}$)	0.947 (0.003)	0.967 (0.020)	0.000	0.000
		CBPS-TLF	$< 10^{-3}$ ($< 10^{-3}$)	0.002 ($< 10^{-3}$)	0.039 ($< 10^{-3}$)	0.031 ($< 10^{-3}$)	0.875 (0.005)	0.615 (0.013)	0.000	0.000
		EB	$< 10^{-3}$ ($< 10^{-3}$)	0.001 ($< 10^{-3}$)	0.038 ($< 10^{-3}$)	0.030 ($< 10^{-3}$)	0.947 (0.003)	0.967 (0.020)	0.000	0.000
		KOM	$< 10^{-3}$ ($< 10^{-3}$)	0.001 ($< 10^{-3}$)	0.038 ($< 10^{-3}$)	0.030 ($< 10^{-3}$)	0.947 (0.003)	0.967 (0.020)	0.000	0.000
ATT	WLS	IPTW	0.008 ($< 10^{-3}$)	0.002 ($< 10^{-3}$)	0.048 ($< 10^{-3}$)	0.038 ($< 10^{-3}$)	0.936 (0.003)	0.963 (0.019)	0.000	0.000
		CBPS-JI	0.005 ($< 10^{-3}$)	0.002 ($< 10^{-3}$)	0.046 ($< 10^{-3}$)	0.037 ($< 10^{-3}$)	0.945 (0.003)	1.000 (0.020)	0.000	0.000
		CBPS-OI	0.013 ($< 10^{-3}$)	0.002 ($< 10^{-3}$)	0.047 ($< 10^{-3}$)	0.037 ($< 10^{-3}$)	0.947 (0.003)	1.127 (0.023)	0.000	0.000
		CBPS-TLF	0.011 ($< 10^{-3}$)	0.002 ($< 10^{-3}$)	0.045 ($< 10^{-3}$)	0.036 ($< 10^{-3}$)	0.940 (0.003)	0.983 (0.020)	0.000	0.000
		EB	0.006 ($< 10^{-3}$)	0.002 ($< 10^{-3}$)	0.046 ($< 10^{-3}$)	0.037 ($< 10^{-3}$)	0.963 (0.003)	1.215 (0.025)	0.000	0.000
		KOM	0.011 ($< 10^{-3}$)	0.002 ($< 10^{-3}$)	0.043 ($< 10^{-3}$)	0.035 ($< 10^{-3}$)	0.952 (0.003)	1.131 (0.023)	0.000	0.000
	DR	IPTW	$< 10^{-3}$ ($< 10^{-3}$)	0.002 ($< 10^{-3}$)	0.045 ($< 10^{-3}$)	0.036 ($< 10^{-3}$)	0.938 (0.003)	0.946 (0.019)	0.000	0.000
		CBPS-JI	$< 10^{-3}$ ($< 10^{-3}$)	0.002 ($< 10^{-3}$)	0.045 ($< 10^{-3}$)	0.036 ($< 10^{-3}$)	0.936 (0.003)	0.928 (0.019)	0.000	0.000
		CBPS-OI	$< 10^{-3}$ ($< 10^{-3}$)	0.002 ($< 10^{-3}$)	0.045 ($< 10^{-3}$)	0.036 ($< 10^{-3}$)	0.942 (0.003)	0.973 (0.020)	0.000	0.000
		CBPS-TLF	$< 10^{-3}$ ($< 10^{-3}$)	0.002 ($< 10^{-3}$)	0.045 ($< 10^{-3}$)	0.036 ($< 10^{-3}$)	0.941 (0.003)	0.938 (0.019)	0.000	0.000
		EB	$< 10^{-3}$ ($< 10^{-3}$)	0.002 ($< 10^{-3}$)	0.047 ($< 10^{-3}$)	0.037 ($< 10^{-3}$)	0.944 (0.003)	0.982 (0.020)	0.000	0.000
		KOM	$< 10^{-3}$ ($< 10^{-3}$)	0.002 ($< 10^{-3}$)	0.044 ($< 10^{-3}$)	0.035 ($< 10^{-3}$)	0.944 (0.003)	0.977 (0.020)	0.000	0.000

Table 17: Scenario: $n = 500$, proportion treated: moderate; complexity: moderate; treatment-effect level: none. Values are reported as estimate (Monte Carlo standard error), except for the missingness columns, which are reported without Monte Carlo standard error.

Estimand	Estimator	Method	Bias	Var	RMSE	MAE	Coverage	Var. ratio	Missingness	
									Point	SE
ATE	WLS	IPTW	0.012 ($< 10^{-3}$)	0.002 ($< 10^{-3}$)	0.048 ($< 10^{-3}$)	0.038 ($< 10^{-3}$)	0.914 (0.004)	0.883 (0.018)	0.000	0.000
		CBPS-JI	0.008 ($< 10^{-3}$)	0.002 ($< 10^{-3}$)	0.044 ($< 10^{-3}$)	0.035 ($< 10^{-3}$)	0.912 (0.004)	0.822 (0.017)	0.000	0.000
		CBPS-OI	0.019 ($< 10^{-3}$)	0.002 ($< 10^{-3}$)	0.046 ($< 10^{-3}$)	0.037 ($< 10^{-3}$)	0.951 (0.003)	1.307 (0.026)	0.000	0.000
		CBPS-TLF	0.047 ($< 10^{-3}$)	0.001 ($< 10^{-3}$)	0.059 ($< 10^{-3}$)	0.050 ($< 10^{-3}$)	0.579 (0.007)	0.600 (0.012)	0.000	0.000
		EB	0.004 ($< 10^{-3}$)	0.002 ($< 10^{-3}$)	0.040 ($< 10^{-3}$)	0.032 ($< 10^{-3}$)	0.981 (0.002)	1.490 (0.030)	0.000	0.000
		KOM	0.010 ($< 10^{-3}$)	0.001 ($< 10^{-3}$)	0.039 ($< 10^{-3}$)	0.031 ($< 10^{-3}$)	0.972 (0.002)	1.423 (0.029)	0.000	0.000
	DR	IPTW	$< 10^{-3}$ ($< 10^{-3}$)	0.002 ($< 10^{-3}$)	0.043 ($< 10^{-3}$)	0.034 ($< 10^{-3}$)	0.929 (0.004)	0.878 (0.018)	0.000	0.000
		CBPS-JI	$< 10^{-3}$ ($< 10^{-3}$)	0.002 ($< 10^{-3}$)	0.042 ($< 10^{-3}$)	0.033 ($< 10^{-3}$)	0.917 (0.004)	0.810 (0.017)	0.000	0.000
		CBPS-OI	$< 10^{-3}$ ($< 10^{-3}$)	0.001 ($< 10^{-3}$)	0.037 ($< 10^{-3}$)	0.030 ($< 10^{-3}$)	0.944 (0.003)	0.953 (0.019)	0.000	0.000
		CBPS-TLF	$< 10^{-3}$ ($< 10^{-3}$)	0.001 ($< 10^{-3}$)	0.037 ($< 10^{-3}$)	0.030 ($< 10^{-3}$)	0.898 (0.004)	0.694 (0.014)	0.000	0.000
		EB	$< 10^{-3}$ ($< 10^{-3}$)	0.001 ($< 10^{-3}$)	0.037 ($< 10^{-3}$)	0.030 ($< 10^{-3}$)	0.944 (0.003)	0.953 (0.019)	0.000	0.000
		KOM	$< 10^{-3}$ ($< 10^{-3}$)	0.001 ($< 10^{-3}$)	0.037 ($< 10^{-3}$)	0.030 ($< 10^{-3}$)	0.944 (0.003)	0.953 (0.019)	0.000	0.000
ATT	WLS	IPTW	0.019 ($< 10^{-3}$)	0.004 ($< 10^{-3}$)	0.067 ($< 10^{-3}$)	0.054 ($< 10^{-3}$)	0.880 (0.005)	0.843 (0.017)	0.000	0.000
		CBPS-JI	0.010 ($< 10^{-3}$)	0.003 ($< 10^{-3}$)	0.057 ($< 10^{-3}$)	0.045 ($< 10^{-3}$)	0.932 (0.004)	1.069 (0.025)	0.000	0.000
		CBPS-OI	0.026 ($< 10^{-3}$)	0.003 ($< 10^{-3}$)	0.061 ($< 10^{-3}$)	0.049 ($< 10^{-3}$)	0.913 (0.004)	1.178 (0.024)	0.000	0.000
		CBPS-TLF	0.059 ($< 10^{-3}$)	0.001 ($< 10^{-3}$)	0.070 ($< 10^{-3}$)	0.061 ($< 10^{-3}$)	0.631 (0.007)	0.911 (0.018)	0.000	0.000
		EB	0.012 ($< 10^{-3}$)	0.002 ($< 10^{-3}$)	0.051 ($< 10^{-3}$)	0.041 ($< 10^{-3}$)	0.972 (0.002)	1.480 (0.030)	0.000	0.000
		KOM	0.015 ($< 10^{-3}$)	0.002 ($< 10^{-3}$)	0.047 ($< 10^{-3}$)	0.038 ($< 10^{-3}$)	0.963 (0.003)	1.391 (0.028)	0.000	0.000
	DR	IPTW	-0.002 ($< 10^{-3}$)	0.003 ($< 10^{-3}$)	0.057 ($< 10^{-3}$)	0.045 ($< 10^{-3}$)	0.919 (0.004)	0.863 (0.018)	0.000	0.000
		CBPS-JI	$< 10^{-3}$ ($< 10^{-3}$)	0.003 ($< 10^{-3}$)	0.054 ($< 10^{-3}$)	0.043 ($< 10^{-3}$)	0.909 (0.004)	0.816 (0.017)	0.000	0.000
		CBPS-OI	$< 10^{-3}$ ($< 10^{-3}$)	0.003 ($< 10^{-3}$)	0.051 ($< 10^{-3}$)	0.041 ($< 10^{-3}$)	0.931 (0.004)	0.943 (0.019)	0.000	0.000
		CBPS-TLF	$< 10^{-3}$ ($< 10^{-3}$)	0.002 ($< 10^{-3}$)	0.046 ($< 10^{-3}$)	0.037 ($< 10^{-3}$)	0.934 (0.004)	0.888 (0.018)	0.000	0.000
		EB	$< 10^{-3}$ ($< 10^{-3}$)	0.003 ($< 10^{-3}$)	0.051 ($< 10^{-3}$)	0.041 ($< 10^{-3}$)	0.938 (0.003)	0.964 (0.020)	0.000	0.000
		KOM	$< 10^{-3}$ ($< 10^{-3}$)	0.002 ($< 10^{-3}$)	0.047 ($< 10^{-3}$)	0.038 ($< 10^{-3}$)	0.940 (0.003)	0.969 (0.020)	0.000	0.000

Table 18: Scenario: $n = 500$, proportion treated: moderate; complexity: high; treatment-effect level: none. Values are reported as estimate (Monte Carlo standard error), except for the missingness columns, which are reported without Monte Carlo standard error.

Estimand	Estimator	Method	Bias	Var	RMSE	MAE	Coverage	Var. ratio	Missingness Point	Missingness SE
ATE	WLS	IPTW	0.027 ($< 10^{-3}$)	0.005 ($< 10^{-3}$)	0.075 ($< 10^{-3}$)	0.059 ($< 10^{-3}$)	0.835 (0.005)	0.667 (0.016)	0.000	0.000
		CBPS-JI	0.018 ($< 10^{-3}$)	0.003 ($< 10^{-3}$)	0.058 ($< 10^{-3}$)	0.047 ($< 10^{-3}$)	0.823 (0.005)	0.569 (0.012)	0.000	0.000
		CBPS-OI	0.039 ($< 10^{-3}$)	0.003 ($< 10^{-3}$)	0.066 ($< 10^{-3}$)	0.055 ($< 10^{-3}$)	0.897 (0.004)	1.310 (0.026)	0.000	0.000
		CBPS-TLF	0.080 ($< 10^{-3}$)	0.001 ($< 10^{-3}$)	0.087 ($< 10^{-3}$)	0.080 ($< 10^{-3}$)	0.161 (0.005)	0.485 (0.010)	0.000	0.000
		EB	0.009 ($< 10^{-3}$)	0.002 ($< 10^{-3}$)	0.040 ($< 10^{-3}$)	0.032 ($< 10^{-3}$)	0.994 (0.001)	2.051 (0.041)	0.000	0.000
		KOM	0.014 ($< 10^{-3}$)	0.001 ($< 10^{-3}$)	0.041 ($< 10^{-3}$)	0.033 ($< 10^{-3}$)	0.987 (0.002)	1.832 (0.036)	0.000	0.000
	DR	IPTW	$< 10^{-3}$ ($< 10^{-3}$)	0.003 ($< 10^{-3}$)	0.058 (0.002)	0.041 ($< 10^{-3}$)	0.913 (0.004)	0.762 (0.017)	0.000	0.000
		CBPS-JI	$< 10^{-3}$ ($< 10^{-3}$)	0.003 ($< 10^{-3}$)	0.050 ($< 10^{-3}$)	0.040 ($< 10^{-3}$)	0.850 (0.005)	0.563 (0.012)	0.000	0.000
		CBPS-OI	$< 10^{-3}$ ($< 10^{-3}$)	0.001 ($< 10^{-3}$)	0.036 ($< 10^{-3}$)	0.029 ($< 10^{-3}$)	0.936 (0.003)	0.923 (0.019)	0.000	0.000
		CBPS-TLF	$< 10^{-3}$ ($< 10^{-3}$)	0.001 ($< 10^{-3}$)	0.036 ($< 10^{-3}$)	0.029 ($< 10^{-3}$)	0.889 (0.004)	0.682 (0.014)	0.000	0.000
		EB	$< 10^{-3}$ ($< 10^{-3}$)	0.001 ($< 10^{-3}$)	0.036 ($< 10^{-3}$)	0.029 ($< 10^{-3}$)	0.936 (0.003)	0.923 (0.019)	0.000	0.000
		KOM	$< 10^{-3}$ ($< 10^{-3}$)	0.001 ($< 10^{-3}$)	0.036 ($< 10^{-3}$)	0.029 ($< 10^{-3}$)	0.936 (0.003)	0.923 (0.019)	0.000	0.000
ATT	WLS	IPTW	0.042 (0.001)	0.011 ($< 10^{-3}$)	0.112 (0.001)	0.091 ($< 10^{-3}$)	0.749 (0.006)	0.611 (0.015)	0.000	0.000
		CBPS-JI	0.018 (0.001)	0.008 ($< 10^{-3}$)	0.092 ($< 10^{-3}$)	0.072 ($< 10^{-3}$)	0.946 (0.003)	3.415 (0.132)	0.000	0.087
		CBPS-OI	0.044 (0.001)	0.008 ($< 10^{-3}$)	0.098 ($< 10^{-3}$)	0.080 ($< 10^{-3}$)	0.845 (0.005)	1.177 (0.028)	0.000	0.000
		CBPS-TLF	0.092 ($< 10^{-3}$)	0.001 ($< 10^{-3}$)	0.099 ($< 10^{-3}$)	0.092 ($< 10^{-3}$)	0.237 (0.006)	0.764 (0.015)	0.000	0.000
		EB	0.031 ($< 10^{-3}$)	0.003 ($< 10^{-3}$)	0.063 ($< 10^{-3}$)	0.051 ($< 10^{-3}$)	0.967 (0.003)	1.858 (0.038)	0.000	0.000
		KOM	0.021 ($< 10^{-3}$)	0.003 ($< 10^{-3}$)	0.056 ($< 10^{-3}$)	0.045 ($< 10^{-3}$)	0.971 (0.002)	1.770 (0.036)	0.000	0.000
	DR	IPTW	-0.003 (0.001)	0.008 ($< 10^{-3}$)	0.087 (0.002)	0.060 ($< 10^{-3}$)	0.895 (0.004)	0.755 (0.017)	0.001	0.001
		CBPS-JI	-0.002 (0.001)	0.007 ($< 10^{-3}$)	0.083 ($< 10^{-3}$)	0.064 ($< 10^{-3}$)	0.907 (0.004)	2.456 (0.271)	0.000	0.093
		CBPS-OI	-0.002 (0.001)	0.005 ($< 10^{-3}$)	0.071 ($< 10^{-3}$)	0.054 ($< 10^{-3}$)	0.908 (0.004)	0.870 (0.021)	0.000	0.000
		CBPS-TLF	-0.002 ($< 10^{-3}$)	0.003 ($< 10^{-3}$)	0.051 ($< 10^{-3}$)	0.041 ($< 10^{-3}$)	0.906 (0.004)	0.791 (0.016)	0.000	0.000
		EB	-0.001 ($< 10^{-3}$)	0.003 ($< 10^{-3}$)	0.058 ($< 10^{-3}$)	0.046 ($< 10^{-3}$)	0.919 (0.004)	0.905 (0.019)	0.000	0.000
		KOM	-0.003 ($< 10^{-3}$)	0.003 ($< 10^{-3}$)	0.055 ($< 10^{-3}$)	0.044 ($< 10^{-3}$)	0.920 (0.004)	0.910 (0.019)	0.000	0.000

Table 19: Scenario: $n = 500$, proportion treated: high; complexity: low; treatment-effect level: none. Values are reported as estimate (Monte Carlo standard error), except for the missingness columns, which are reported without Monte Carlo standard error.

Estimand	Estimator	Method	Bias	Var	RMSE	MAE	Coverage	Var. ratio	Missingness	
									Point	SE
ATE	WLS	IPTW	0.005 ($< 10^{-3}$)	0.003 ($< 10^{-3}$)	0.053 ($< 10^{-3}$)	0.042 ($< 10^{-3}$)	0.922 (0.004)	0.901 (0.019)	0.000	0.000
		CBPS-JI	0.003 ($< 10^{-3}$)	0.003 ($< 10^{-3}$)	0.050 ($< 10^{-3}$)	0.040 ($< 10^{-3}$)	0.913 (0.004)	0.816 (0.017)	0.000	0.000
		CBPS-OI	0.009 ($< 10^{-3}$)	0.002 ($< 10^{-3}$)	0.050 ($< 10^{-3}$)	0.040 ($< 10^{-3}$)	0.943 (0.003)	1.132 (0.023)	0.000	0.000
		CBPS-TLF	0.021 ($< 10^{-3}$)	0.002 ($< 10^{-3}$)	0.049 ($< 10^{-3}$)	0.039 ($< 10^{-3}$)	0.751 (0.006)	0.453 (0.009)	0.000	0.000
		EB	0.003 ($< 10^{-3}$)	0.003 ($< 10^{-3}$)	0.050 ($< 10^{-3}$)	0.040 ($< 10^{-3}$)	0.962 (0.003)	1.201 (0.024)	0.000	0.000
		KOM	0.012 ($< 10^{-3}$)	0.002 ($< 10^{-3}$)	0.047 ($< 10^{-3}$)	0.038 ($< 10^{-3}$)	0.942 (0.003)	1.104 (0.022)	0.000	0.000
	DR	IPTW	-0.002 ($< 10^{-3}$)	0.003 ($< 10^{-3}$)	0.052 ($< 10^{-3}$)	0.041 ($< 10^{-3}$)	0.919 (0.004)	0.865 (0.019)	0.000	0.000
		CBPS-JI	-0.001 ($< 10^{-3}$)	0.003 ($< 10^{-3}$)	0.051 ($< 10^{-3}$)	0.040 ($< 10^{-3}$)	0.911 (0.004)	0.801 (0.017)	0.000	0.000
		CBPS-OI	-0.001 ($< 10^{-3}$)	0.002 ($< 10^{-3}$)	0.048 ($< 10^{-3}$)	0.038 ($< 10^{-3}$)	0.933 (0.004)	0.930 (0.019)	0.000	0.000
		CBPS-TLF	-0.001 ($< 10^{-3}$)	0.002 ($< 10^{-3}$)	0.049 ($< 10^{-3}$)	0.039 ($< 10^{-3}$)	0.818 (0.005)	0.479 (0.010)	0.000	0.000
		EB	-0.001 ($< 10^{-3}$)	0.002 ($< 10^{-3}$)	0.048 ($< 10^{-3}$)	0.038 ($< 10^{-3}$)	0.933 (0.004)	0.930 (0.019)	0.000	0.000
		KOM	-0.001 ($< 10^{-3}$)	0.002 ($< 10^{-3}$)	0.048 ($< 10^{-3}$)	0.038 ($< 10^{-3}$)	0.933 (0.004)	0.930 (0.019)	0.000	0.000
ATT	WLS	IPTW	0.006 ($< 10^{-3}$)	0.004 ($< 10^{-3}$)	0.061 ($< 10^{-3}$)	0.048 ($< 10^{-3}$)	0.910 (0.004)	0.889 (0.019)	0.000	0.000
		CBPS-JI	0.003 ($< 10^{-3}$)	0.003 ($< 10^{-3}$)	0.058 ($< 10^{-3}$)	0.046 ($< 10^{-3}$)	0.901 (0.004)	0.786 (0.017)	0.000	0.000
		CBPS-OI	0.010 ($< 10^{-3}$)	0.003 ($< 10^{-3}$)	0.056 ($< 10^{-3}$)	0.045 ($< 10^{-3}$)	0.933 (0.004)	1.104 (0.023)	0.000	0.000
		CBPS-TLF	0.024 ($< 10^{-3}$)	0.002 ($< 10^{-3}$)	0.051 ($< 10^{-3}$)	0.041 ($< 10^{-3}$)	0.858 (0.005)	0.784 (0.016)	0.000	0.000
		EB	0.007 ($< 10^{-3}$)	0.003 ($< 10^{-3}$)	0.055 ($< 10^{-3}$)	0.044 ($< 10^{-3}$)	0.953 (0.003)	1.192 (0.024)	0.000	0.000
		KOM	0.016 ($< 10^{-3}$)	0.002 ($< 10^{-3}$)	0.051 ($< 10^{-3}$)	0.041 ($< 10^{-3}$)	0.930 (0.004)	1.067 (0.022)	0.000	0.000
	DR	IPTW	-0.002 ($< 10^{-3}$)	0.003 ($< 10^{-3}$)	0.059 ($< 10^{-3}$)	0.046 ($< 10^{-3}$)	0.912 (0.004)	0.851 (0.019)	0.000	0.000
		CBPS-JI	-0.002 ($< 10^{-3}$)	0.003 ($< 10^{-3}$)	0.058 ($< 10^{-3}$)	0.046 ($< 10^{-3}$)	0.893 (0.004)	0.751 (0.016)	0.000	0.000
		CBPS-OI	-0.002 ($< 10^{-3}$)	0.003 ($< 10^{-3}$)	0.056 ($< 10^{-3}$)	0.045 ($< 10^{-3}$)	0.922 (0.004)	0.911 (0.019)	0.000	0.000
		CBPS-TLF	-0.001 ($< 10^{-3}$)	0.003 ($< 10^{-3}$)	0.054 ($< 10^{-3}$)	0.043 ($< 10^{-3}$)	0.890 (0.004)	0.694 (0.014)	0.000	0.000
		EB	$< 10^{-3}$ ($< 10^{-3}$)	0.003 ($< 10^{-3}$)	0.057 ($< 10^{-3}$)	0.046 ($< 10^{-3}$)	0.923 (0.004)	0.917 (0.019)	0.000	0.000
		KOM	-0.001 ($< 10^{-3}$)	0.003 ($< 10^{-3}$)	0.054 ($< 10^{-3}$)	0.043 ($< 10^{-3}$)	0.929 (0.004)	0.927 (0.019)	0.000	0.000

Table 20: Scenario: $n = 500$, proportion treated: high; complexity: moderate; treatment-effect level: none. Values are reported as estimate (Monte Carlo standard error), except for the missingness columns, which are reported without Monte Carlo standard error.

Estimand	Estimator	Method	Bias	Var	RMSE	MAE	Coverage	Var. ratio	Missingness	
									Point	SE
ATE	WLS	IPTW	0.013 (0.001)	0.005 ($< 10^{-3}$)	0.073 ($< 10^{-3}$)	0.058 ($< 10^{-3}$)	0.861 (0.005)	0.769 (0.018)	0.000	0.000
		CBPS-JI	0.008 ($< 10^{-3}$)	0.003 ($< 10^{-3}$)	0.059 ($< 10^{-3}$)	0.047 ($< 10^{-3}$)	0.862 (0.005)	0.629 (0.013)	0.000	0.000
		CBPS-OI	0.021 ($< 10^{-3}$)	0.003 ($< 10^{-3}$)	0.062 ($< 10^{-3}$)	0.050 ($< 10^{-3}$)	0.908 (0.004)	1.203 (0.025)	0.000	0.000
		CBPS-TLF	0.044 ($< 10^{-3}$)	0.002 ($< 10^{-3}$)	0.062 ($< 10^{-3}$)	0.051 ($< 10^{-3}$)	0.543 (0.007)	0.361 (0.007)	0.000	0.000
		EB	0.007 ($< 10^{-3}$)	0.003 ($< 10^{-3}$)	0.052 ($< 10^{-3}$)	0.041 ($< 10^{-3}$)	0.969 (0.002)	1.464 (0.030)	0.000	0.000
		KOM	0.019 ($< 10^{-3}$)	0.002 ($< 10^{-3}$)	0.052 ($< 10^{-3}$)	0.042 ($< 10^{-3}$)	0.938 (0.003)	1.243 (0.025)	0.000	0.000
	DR	IPTW	-0.001 ($< 10^{-3}$)	0.004 ($< 10^{-3}$)	0.066 (0.001)	0.051 ($< 10^{-3}$)	0.890 (0.004)	0.744 (0.017)	0.000	0.000
		CBPS-JI	$< 10^{-3}$ ($< 10^{-3}$)	0.003 ($< 10^{-3}$)	0.059 ($< 10^{-3}$)	0.047 ($< 10^{-3}$)	0.853 (0.005)	0.604 (0.012)	0.000	0.000
		CBPS-OI	-0.001 ($< 10^{-3}$)	0.003 ($< 10^{-3}$)	0.053 ($< 10^{-3}$)	0.043 ($< 10^{-3}$)	0.911 (0.004)	0.856 (0.018)	0.000	0.000
		CBPS-TLF	$< 10^{-3}$ ($< 10^{-3}$)	0.003 ($< 10^{-3}$)	0.053 ($< 10^{-3}$)	0.043 ($< 10^{-3}$)	0.793 (0.006)	0.449 (0.009)	0.000	0.000
		EB	-0.001 ($< 10^{-3}$)	0.003 ($< 10^{-3}$)	0.053 ($< 10^{-3}$)	0.043 ($< 10^{-3}$)	0.911 (0.004)	0.856 (0.018)	0.000	0.000
		KOM	-0.001 ($< 10^{-3}$)	0.003 ($< 10^{-3}$)	0.053 ($< 10^{-3}$)	0.043 ($< 10^{-3}$)	0.911 (0.004)	0.856 (0.018)	0.000	0.000
ATT	WLS	IPTW	0.016 (0.001)	0.008 ($< 10^{-3}$)	0.090 ($< 10^{-3}$)	0.072 ($< 10^{-3}$)	0.836 (0.005)	0.734 (0.016)	0.000	0.000
		CBPS-JI	0.009 (0.001)	0.006 ($< 10^{-3}$)	0.079 ($< 10^{-3}$)	0.063 ($< 10^{-3}$)	0.869 (0.005)	1.035 (0.033)	0.000	0.009
		CBPS-OI	0.023 (0.001)	0.005 ($< 10^{-3}$)	0.076 ($< 10^{-3}$)	0.062 ($< 10^{-3}$)	0.888 (0.004)	1.120 (0.023)	0.000	0.000
		CBPS-TLF	0.049 ($< 10^{-3}$)	0.002 ($< 10^{-3}$)	0.067 ($< 10^{-3}$)	0.056 ($< 10^{-3}$)	0.678 (0.007)	0.628 (0.013)	0.000	0.000
		EB	0.019 ($< 10^{-3}$)	0.004 ($< 10^{-3}$)	0.063 ($< 10^{-3}$)	0.051 ($< 10^{-3}$)	0.947 (0.003)	1.393 (0.028)	0.000	0.000
		KOM	0.025 ($< 10^{-3}$)	0.003 ($< 10^{-3}$)	0.060 ($< 10^{-3}$)	0.049 ($< 10^{-3}$)	0.923 (0.004)	1.198 (0.024)	0.000	0.000
	DR	IPTW	-0.001 (0.001)	0.007 ($< 10^{-3}$)	0.081 (0.001)	0.062 ($< 10^{-3}$)	0.875 (0.005)	0.733 (0.015)	0.000	0.000
		CBPS-JI	$< 10^{-3}$ (0.001)	0.006 ($< 10^{-3}$)	0.077 ($< 10^{-3}$)	0.062 ($< 10^{-3}$)	0.836 (0.005)	0.774 (0.030)	0.000	0.010
		CBPS-OI	$< 10^{-3}$ ($< 10^{-3}$)	0.005 ($< 10^{-3}$)	0.069 ($< 10^{-3}$)	0.056 ($< 10^{-3}$)	0.889 (0.004)	0.832 (0.018)	0.000	0.000
		CBPS-TLF	$< 10^{-3}$ ($< 10^{-3}$)	0.004 ($< 10^{-3}$)	0.064 ($< 10^{-3}$)	0.051 ($< 10^{-3}$)	0.848 (0.005)	0.572 (0.012)	0.000	0.000
		EB	$< 10^{-3}$ ($< 10^{-3}$)	0.005 ($< 10^{-3}$)	0.067 ($< 10^{-3}$)	0.054 ($< 10^{-3}$)	0.901 (0.004)	0.838 (0.017)	0.000	0.000
		KOM	$< 10^{-3}$ ($< 10^{-3}$)	0.004 ($< 10^{-3}$)	0.064 ($< 10^{-3}$)	0.052 ($< 10^{-3}$)	0.901 (0.004)	0.844 (0.017)	0.000	0.000

Table 21: Scenario: $n = 500$, proportion treated: high; complexity: high; treatment-effect level: none. Values are reported as estimate (Monte Carlo standard error), except for the missingness columns, which are reported without Monte Carlo standard error.

Estimand	Estimator	Method	Bias	Var	RMSE	MAE	Coverage	Var. ratio	Missingness Point	Missingness SE
ATE	WLS	IPTW	0.032 (0.002)	0.014 ($< 10^{-3}$)	0.122 (0.002)	0.097 (0.001)	0.693 (0.007)	0.477 (0.013)	0.000	0.000
		CBPS-JI	0.012 (0.001)	0.006 ($< 10^{-3}$)	0.078 ($< 10^{-3}$)	0.063 ($< 10^{-3}$)	0.726 (0.006)	0.363 (0.007)	0.000	0.000
		CBPS-OI	0.040 (0.001)	0.008 ($< 10^{-3}$)	0.096 ($< 10^{-3}$)	0.080 ($< 10^{-3}$)	0.787 (0.006)	0.977 (0.020)	0.000	0.000
		CBPS-TLF	0.079 ($< 10^{-3}$)	0.002 ($< 10^{-3}$)	0.090 ($< 10^{-3}$)	0.081 ($< 10^{-3}$)	0.209 (0.006)	0.265 (0.005)	0.000	0.000
		EB	0.015 ($< 10^{-3}$)	0.003 ($< 10^{-3}$)	0.055 ($< 10^{-3}$)	0.044 ($< 10^{-3}$)	0.975 (0.002)	1.898 (0.038)	0.000	0.000
		KOM	0.031 ($< 10^{-3}$)	0.003 ($< 10^{-3}$)	0.062 ($< 10^{-3}$)	0.050 ($< 10^{-3}$)	0.917 (0.004)	1.441 (0.028)	0.000	0.000
	DR	IPTW	0.003 (0.001)	0.010 ($< 10^{-3}$)	0.099 (0.003)	0.068 (0.001)	0.794 (0.006)	0.667 (0.023)	0.001	0.001
		CBPS-JI	0.003 (0.001)	0.006 ($< 10^{-3}$)	0.079 ($< 10^{-3}$)	0.063 ($< 10^{-3}$)	0.714 (0.006)	0.366 (0.008)	0.000	0.002
		CBPS-OI	0.001 ($< 10^{-3}$)	0.004 ($< 10^{-3}$)	0.064 ($< 10^{-3}$)	0.052 ($< 10^{-3}$)	0.807 (0.006)	0.626 (0.014)	0.001	0.001
		CBPS-TLF	0.001 ($< 10^{-3}$)	0.004 ($< 10^{-3}$)	0.065 ($< 10^{-3}$)	0.052 ($< 10^{-3}$)	0.716 (0.006)	0.399 (0.009)	0.000	0.001
		EB	0.001 ($< 10^{-3}$)	0.004 ($< 10^{-3}$)	0.064 ($< 10^{-3}$)	0.052 ($< 10^{-3}$)	0.807 (0.006)	0.626 (0.014)	0.001	0.001
		KOM	0.001 ($< 10^{-3}$)	0.004 ($< 10^{-3}$)	0.064 ($< 10^{-3}$)	0.052 ($< 10^{-3}$)	0.807 (0.006)	0.626 (0.014)	0.001	0.001
ATT	WLS	IPTW	0.038 (0.002)	0.021 ($< 10^{-3}$)	0.151 (0.002)	0.123 (0.001)	0.671 (0.007)	0.459 (0.012)	0.000	0.000
		CBPS-JI	0.027 (0.002)	0.018 ($< 10^{-3}$)	0.135 (0.001)	0.110 (0.001)	0.874 (0.010)	4.826 (1.182)	0.000	0.774
		CBPS-OI	0.031 (0.002)	0.018 ($< 10^{-3}$)	0.136 (0.001)	0.112 (0.001)	0.835 (0.005)	1.335 (0.027)	0.000	0.000
		CBPS-TLF	0.082 ($< 10^{-3}$)	0.002 ($< 10^{-3}$)	0.095 ($< 10^{-3}$)	0.084 ($< 10^{-3}$)	0.342 (0.007)	0.478 (0.009)	0.000	0.000
		EB	0.042 ($< 10^{-3}$)	0.005 ($< 10^{-3}$)	0.080 ($< 10^{-3}$)	0.065 ($< 10^{-3}$)	0.923 (0.004)	1.623 (0.033)	0.000	0.000
		KOM	0.040 ($< 10^{-3}$)	0.004 ($< 10^{-3}$)	0.076 ($< 10^{-3}$)	0.062 ($< 10^{-3}$)	0.897 (0.004)	1.383 (0.028)	0.000	0.000
	DR	IPTW	0.005 (0.002)	0.015 ($< 10^{-3}$)	0.122 (0.003)	0.086 (0.001)	0.777 (0.006)	0.659 (0.027)	0.002	0.002
		CBPS-JI	0.005 (0.002)	0.014 ($< 10^{-3}$)	0.120 (0.001)	0.095 (0.001)	0.833 (0.012)	3.043 (0.372)	0.000	0.790
		CBPS-OI	0.005 (0.002)	0.012 ($< 10^{-3}$)	0.111 (0.001)	0.088 ($< 10^{-3}$)	0.794 (0.006)	0.645 (0.014)	0.001	0.001
		CBPS-TLF	0.002 (0.001)	0.007 ($< 10^{-3}$)	0.082 ($< 10^{-3}$)	0.066 ($< 10^{-3}$)	0.742 (0.006)	0.446 (0.010)	0.000	0.001
		EB	0.003 (0.001)	0.007 ($< 10^{-3}$)	0.086 ($< 10^{-3}$)	0.069 ($< 10^{-3}$)	0.792 (0.006)	0.614 (0.014)	0.001	0.001
		KOM	0.001 (0.001)	0.007 ($< 10^{-3}$)	0.084 ($< 10^{-3}$)	0.067 ($< 10^{-3}$)	0.789 (0.006)	0.610 (0.013)	0.001	0.001

Table 22: Scenario: $n = 1000$, proportion treated: low; complexity: low; treatment-effect level: none. Values are reported as estimate (Monte Carlo standard error), except for the missingness columns, which are reported without Monte Carlo standard error.

Estimand	Estimator	Method	Bias	Var	RMSE	MAE	Coverage	Var. ratio	Missingness Point	Missingness SE
ATE	WLS	IPTW	0.002 ($< 10^{-3}$)	0.001 ($< 10^{-3}$)	0.034 ($< 10^{-3}$)	0.027 ($< 10^{-3}$)	0.938 (0.003)	0.916 (0.018)	0.000	0.000
		CBPS-JI	0.005 ($< 10^{-3}$)	0.001 ($< 10^{-3}$)	0.034 ($< 10^{-3}$)	0.027 ($< 10^{-3}$)	0.934 (0.004)	0.872 (0.018)	0.000	0.000
		CBPS-OI	0.008 ($< 10^{-3}$)	0.001 ($< 10^{-3}$)	0.035 ($< 10^{-3}$)	0.028 ($< 10^{-3}$)	0.960 (0.003)	1.100 (0.022)	0.000	0.000
		CBPS-TLF	0.006 ($< 10^{-3}$)	0.001 ($< 10^{-3}$)	0.034 ($< 10^{-3}$)	0.027 ($< 10^{-3}$)	0.811 (0.006)	0.457 (0.009)	0.000	0.000
		EB	0.001 ($< 10^{-3}$)	0.001 ($< 10^{-3}$)	0.035 ($< 10^{-3}$)	0.028 ($< 10^{-3}$)	0.967 (0.003)	1.179 (0.023)	0.000	0.000
		KOM	0.006 ($< 10^{-3}$)	0.001 ($< 10^{-3}$)	0.032 ($< 10^{-3}$)	0.026 ($< 10^{-3}$)	0.960 (0.003)	1.139 (0.023)	0.000	0.000
	DR	IPTW	$< 10^{-3}$ ($< 10^{-3}$)	0.001 ($< 10^{-3}$)	0.034 ($< 10^{-3}$)	0.027 ($< 10^{-3}$)	0.936 (0.003)	0.907 (0.018)	0.000	0.000
		CBPS-JI	$< 10^{-3}$ ($< 10^{-3}$)	0.001 ($< 10^{-3}$)	0.034 ($< 10^{-3}$)	0.027 ($< 10^{-3}$)	0.930 (0.004)	0.873 (0.017)	0.000	0.000
		CBPS-OI	$< 10^{-3}$ ($< 10^{-3}$)	0.001 ($< 10^{-3}$)	0.032 ($< 10^{-3}$)	0.026 ($< 10^{-3}$)	0.941 (0.003)	0.931 (0.019)	0.000	0.000
		CBPS-TLF	$< 10^{-3}$ ($< 10^{-3}$)	0.001 ($< 10^{-3}$)	0.034 ($< 10^{-3}$)	0.027 ($< 10^{-3}$)	0.811 (0.006)	0.460 (0.009)	0.000	0.000
		EB	$< 10^{-3}$ ($< 10^{-3}$)	0.001 ($< 10^{-3}$)	0.032 ($< 10^{-3}$)	0.026 ($< 10^{-3}$)	0.941 (0.003)	0.931 (0.019)	0.000	0.000
		KOM	$< 10^{-3}$ ($< 10^{-3}$)	0.001 ($< 10^{-3}$)	0.032 ($< 10^{-3}$)	0.026 ($< 10^{-3}$)	0.941 (0.003)	0.931 (0.019)	0.000	0.000
ATT	WLS	IPTW	0.010 ($< 10^{-3}$)	0.001 ($< 10^{-3}$)	0.036 ($< 10^{-3}$)	0.029 ($< 10^{-3}$)	0.934 (0.004)	0.956 (0.019)	0.000	0.000
		CBPS-JI	0.005 ($< 10^{-3}$)	0.001 ($< 10^{-3}$)	0.035 ($< 10^{-3}$)	0.028 ($< 10^{-3}$)	0.955 (0.003)	1.095 (0.022)	0.000	0.000
		CBPS-OI	0.015 ($< 10^{-3}$)	0.001 ($< 10^{-3}$)	0.038 ($< 10^{-3}$)	0.030 ($< 10^{-3}$)	0.941 (0.003)	1.103 (0.022)	0.000	0.000
		CBPS-TLF	0.006 ($< 10^{-3}$)	0.001 ($< 10^{-3}$)	0.036 ($< 10^{-3}$)	0.029 ($< 10^{-3}$)	0.935 (0.003)	0.929 (0.018)	0.000	0.000
		EB	0.003 ($< 10^{-3}$)	0.001 ($< 10^{-3}$)	0.037 ($< 10^{-3}$)	0.029 ($< 10^{-3}$)	0.971 (0.002)	1.244 (0.025)	0.000	0.000
		KOM	0.006 ($< 10^{-3}$)	0.001 ($< 10^{-3}$)	0.034 ($< 10^{-3}$)	0.027 ($< 10^{-3}$)	0.964 (0.003)	1.170 (0.024)	0.000	0.000
	DR	IPTW	$< 10^{-3}$ ($< 10^{-3}$)	0.001 ($< 10^{-3}$)	0.034 ($< 10^{-3}$)	0.027 ($< 10^{-3}$)	0.941 (0.003)	0.953 (0.019)	0.000	0.000
		CBPS-JI	$< 10^{-3}$ ($< 10^{-3}$)	0.001 ($< 10^{-3}$)	0.034 ($< 10^{-3}$)	0.027 ($< 10^{-3}$)	0.942 (0.003)	0.964 (0.020)	0.000	0.000
		CBPS-OI	$< 10^{-3}$ ($< 10^{-3}$)	0.001 ($< 10^{-3}$)	0.034 ($< 10^{-3}$)	0.027 ($< 10^{-3}$)	0.943 (0.003)	0.959 (0.020)	0.000	0.000
		CBPS-TLF	$< 10^{-3}$ ($< 10^{-3}$)	0.001 ($< 10^{-3}$)	0.036 ($< 10^{-3}$)	0.029 ($< 10^{-3}$)	0.938 (0.003)	0.925 (0.018)	0.000	0.000
		EB	$< 10^{-3}$ ($< 10^{-3}$)	0.001 ($< 10^{-3}$)	0.037 ($< 10^{-3}$)	0.029 ($< 10^{-3}$)	0.947 (0.003)	0.997 (0.020)	0.000	0.000
		KOM	$< 10^{-3}$ ($< 10^{-3}$)	0.001 ($< 10^{-3}$)	0.034 ($< 10^{-3}$)	0.027 ($< 10^{-3}$)	0.947 (0.003)	0.966 (0.020)	0.000	0.000

Table 23: Scenario: $n = 1000$, proportion treated: low; complexity: moderate; treatment-effect level: none. Values are reported as estimate (Monte Carlo standard error), except for the missingness columns, which are reported without Monte Carlo standard error.

Estimand	Estimator	Method	Bias	Var	RMSE	MAE	Coverage	Var. ratio	Missingness Point	Missingness SE
ATE	WLS	IPTW	0.006 ($< 10^{-3}$)	0.002 ($< 10^{-3}$)	0.040 ($< 10^{-3}$)	0.031 ($< 10^{-3}$)	0.935 (0.003)	0.894 (0.019)	0.000	0.000
		CBPS-JI	0.013 ($< 10^{-3}$)	0.001 ($< 10^{-3}$)	0.040 ($< 10^{-3}$)	0.031 ($< 10^{-3}$)	0.902 (0.004)	0.772 (0.016)	0.000	0.000
		CBPS-OI	0.019 ($< 10^{-3}$)	0.001 ($< 10^{-3}$)	0.041 ($< 10^{-3}$)	0.033 ($< 10^{-3}$)	0.965 (0.003)	1.289 (0.026)	0.000	0.000
		CBPS-TLF	0.051 ($< 10^{-3}$)	$< 10^{-3}$ ($< 10^{-3}$)	0.059 ($< 10^{-3}$)	0.052 ($< 10^{-3}$)	0.360 (0.007)	0.509 (0.010)	0.000	0.000
		EB	0.004 ($< 10^{-3}$)	0.001 ($< 10^{-3}$)	0.036 ($< 10^{-3}$)	0.028 ($< 10^{-3}$)	0.983 (0.002)	1.529 (0.031)	0.000	0.000
		KOM	0.009 ($< 10^{-3}$)	0.001 ($< 10^{-3}$)	0.033 ($< 10^{-3}$)	0.027 ($< 10^{-3}$)	0.984 (0.002)	1.490 (0.029)	0.000	0.000
	DR	IPTW	$< 10^{-3}$ ($< 10^{-3}$)	0.001 ($< 10^{-3}$)	0.038 ($< 10^{-3}$)	0.029 ($< 10^{-3}$)	0.924 (0.004)	0.882 (0.018)	0.000	0.000
		CBPS-JI	$< 10^{-3}$ ($< 10^{-3}$)	0.001 ($< 10^{-3}$)	0.036 ($< 10^{-3}$)	0.029 ($< 10^{-3}$)	0.905 (0.004)	0.777 (0.016)	0.000	0.000
		CBPS-OI	0.001 ($< 10^{-3}$)	$< 10^{-3}$ ($< 10^{-3}$)	0.032 ($< 10^{-3}$)	0.025 ($< 10^{-3}$)	0.942 (0.003)	0.966 (0.019)	0.000	0.000
		CBPS-TLF	0.002 ($< 10^{-3}$)	0.001 ($< 10^{-3}$)	0.032 ($< 10^{-3}$)	0.025 ($< 10^{-3}$)	0.853 (0.005)	0.573 (0.011)	0.000	0.000
		EB	0.001 ($< 10^{-3}$)	$< 10^{-3}$ ($< 10^{-3}$)	0.032 ($< 10^{-3}$)	0.025 ($< 10^{-3}$)	0.942 (0.003)	0.966 (0.019)	0.000	0.000
		KOM	0.001 ($< 10^{-3}$)	$< 10^{-3}$ ($< 10^{-3}$)	0.032 ($< 10^{-3}$)	0.025 ($< 10^{-3}$)	0.942 (0.003)	0.966 (0.019)	0.000	0.000
ATT	WLS	IPTW	0.028 ($< 10^{-3}$)	0.002 ($< 10^{-3}$)	0.049 ($< 10^{-3}$)	0.040 ($< 10^{-3}$)	0.872 (0.005)	0.995 (0.021)	0.000	0.000
		CBPS-JI	0.015 ($< 10^{-3}$)	0.001 ($< 10^{-3}$)	0.040 ($< 10^{-3}$)	0.032 ($< 10^{-3}$)	0.973 (0.002)	1.560 (0.032)	0.000	0.000
		CBPS-OI	0.036 ($< 10^{-3}$)	0.002 ($< 10^{-3}$)	0.053 ($< 10^{-3}$)	0.043 ($< 10^{-3}$)	0.892 (0.004)	1.305 (0.027)	0.000	0.000
		CBPS-TLF	0.038 ($< 10^{-3}$)	0.001 ($< 10^{-3}$)	0.049 ($< 10^{-3}$)	0.041 ($< 10^{-3}$)	0.775 (0.006)	0.966 (0.020)	0.000	0.000
		EB	0.008 ($< 10^{-3}$)	0.001 ($< 10^{-3}$)	0.038 ($< 10^{-3}$)	0.030 ($< 10^{-3}$)	0.987 (0.002)	1.667 (0.035)	0.000	0.000
		KOM	0.008 ($< 10^{-3}$)	0.001 ($< 10^{-3}$)	0.035 ($< 10^{-3}$)	0.028 ($< 10^{-3}$)	0.986 (0.002)	1.610 (0.033)	0.000	0.000
	DR	IPTW	$< 10^{-3}$ ($< 10^{-3}$)	0.001 ($< 10^{-3}$)	0.037 ($< 10^{-3}$)	0.029 ($< 10^{-3}$)	0.946 (0.003)	0.978 (0.020)	0.000	0.000
		CBPS-JI	0.001 ($< 10^{-3}$)	0.001 ($< 10^{-3}$)	0.036 ($< 10^{-3}$)	0.029 ($< 10^{-3}$)	0.951 (0.003)	1.016 (0.021)	0.000	0.000
		CBPS-OI	$< 10^{-3}$ ($< 10^{-3}$)	0.001 ($< 10^{-3}$)	0.035 ($< 10^{-3}$)	0.028 ($< 10^{-3}$)	0.951 (0.003)	1.009 (0.021)	0.000	0.000
		CBPS-TLF	0.002 ($< 10^{-3}$)	0.001 ($< 10^{-3}$)	0.033 ($< 10^{-3}$)	0.027 ($< 10^{-3}$)	0.944 (0.003)	0.970 (0.020)	0.000	0.000
		EB	0.001 ($< 10^{-3}$)	0.001 ($< 10^{-3}$)	0.037 ($< 10^{-3}$)	0.029 ($< 10^{-3}$)	0.956 (0.003)	1.055 (0.022)	0.000	0.000
		KOM	$< 10^{-3}$ ($< 10^{-3}$)	0.001 ($< 10^{-3}$)	0.034 ($< 10^{-3}$)	0.027 ($< 10^{-3}$)	0.953 (0.003)	1.043 (0.021)	0.000	0.000

Table 24: Scenario: $n = 1000$, proportion treated: low; complexity: high; treatment-effect level: none. Values are reported as estimate (Monte Carlo standard error), except for the missingness columns, which are reported without Monte Carlo standard error.

Estimand	Estimator	Method	Bias	Var	RMSE	MAE	Coverage	Var. ratio	Missingness Point	Missingness SE
ATE	WLS	IPTW	0.009 ($< 10^{-3}$)	0.003 ($< 10^{-3}$)	0.059 ($< 10^{-3}$)	0.046 ($< 10^{-3}$)	0.913 (0.004)	0.731 (0.015)	0.000	0.000
		CBPS-JI	0.031 ($< 10^{-3}$)	0.003 ($< 10^{-3}$)	0.059 ($< 10^{-3}$)	0.047 ($< 10^{-3}$)	0.776 (0.006)	0.536 (0.011)	0.000	0.000
		CBPS-OI	0.041 ($< 10^{-3}$)	0.002 ($< 10^{-3}$)	0.062 ($< 10^{-3}$)	0.050 ($< 10^{-3}$)	0.947 (0.003)	1.388 (0.028)	0.000	0.000
		CBPS-TLF	0.089 ($< 10^{-3}$)	$< 10^{-3}$ ($< 10^{-3}$)	0.093 ($< 10^{-3}$)	0.089 ($< 10^{-3}$)	0.023 (0.002)	0.402 (0.008)	0.000	0.000
		EB	0.012 ($< 10^{-3}$)	0.001 ($< 10^{-3}$)	0.039 ($< 10^{-3}$)	0.031 ($< 10^{-3}$)	0.996 ($< 10^{-3}$)	2.234 (0.046)	0.000	0.000
		KOM	0.017 ($< 10^{-3}$)	0.001 ($< 10^{-3}$)	0.039 ($< 10^{-3}$)	0.031 ($< 10^{-3}$)	0.992 (0.001)	1.989 (0.041)	0.000	0.000
	DR	IPTW	0.001 ($< 10^{-3}$)	0.002 ($< 10^{-3}$)	0.045 (0.002)	0.032 ($< 10^{-3}$)	0.920 (0.004)	0.913 (0.074)	0.000	0.000
		CBPS-JI	0.002 ($< 10^{-3}$)	0.002 ($< 10^{-3}$)	0.045 ($< 10^{-3}$)	0.035 ($< 10^{-3}$)	0.851 (0.005)	0.569 (0.012)	0.000	0.000
		CBPS-OI	0.004 ($< 10^{-3}$)	$< 10^{-3}$ ($< 10^{-3}$)	0.032 ($< 10^{-3}$)	0.025 ($< 10^{-3}$)	0.941 (0.003)	0.959 (0.020)	0.000	0.000
		CBPS-TLF	0.004 ($< 10^{-3}$)	$< 10^{-3}$ ($< 10^{-3}$)	0.032 ($< 10^{-3}$)	0.025 ($< 10^{-3}$)	0.862 (0.005)	0.593 (0.012)	0.000	0.000
		EB	0.004 ($< 10^{-3}$)	$< 10^{-3}$ ($< 10^{-3}$)	0.032 ($< 10^{-3}$)	0.025 ($< 10^{-3}$)	0.941 (0.003)	0.959 (0.020)	0.000	0.000
		KOM	0.004 ($< 10^{-3}$)	$< 10^{-3}$ ($< 10^{-3}$)	0.032 ($< 10^{-3}$)	0.025 ($< 10^{-3}$)	0.941 (0.003)	0.959 (0.020)	0.000	0.000
ATT	WLS	IPTW	0.053 ($< 10^{-3}$)	0.004 ($< 10^{-3}$)	0.082 ($< 10^{-3}$)	0.068 ($< 10^{-3}$)	0.745 (0.006)	0.812 (0.019)	0.000	0.000
		CBPS-JI	0.024 ($< 10^{-3}$)	0.002 ($< 10^{-3}$)	0.055 ($< 10^{-3}$)	0.044 ($< 10^{-3}$)	0.985 (0.002)	2.875 (0.062)	0.000	0.000
		CBPS-OI	0.060 ($< 10^{-3}$)	0.003 ($< 10^{-3}$)	0.079 ($< 10^{-3}$)	0.067 ($< 10^{-3}$)	0.806 (0.006)	1.277 (0.026)	0.000	0.000
		CBPS-TLF	0.058 ($< 10^{-3}$)	0.001 ($< 10^{-3}$)	0.066 ($< 10^{-3}$)	0.059 ($< 10^{-3}$)	0.498 (0.007)	0.807 (0.016)	0.000	0.000
		EB	0.020 ($< 10^{-3}$)	0.002 ($< 10^{-3}$)	0.045 ($< 10^{-3}$)	0.036 ($< 10^{-3}$)	0.988 (0.002)	2.202 (0.045)	0.000	0.000
		KOM	0.012 ($< 10^{-3}$)	0.001 ($< 10^{-3}$)	0.040 ($< 10^{-3}$)	0.032 ($< 10^{-3}$)	0.992 (0.001)	2.129 (0.043)	0.000	0.000
	DR	IPTW	-0.003 ($< 10^{-3}$)	0.002 ($< 10^{-3}$)	0.047 ($< 10^{-3}$)	0.035 ($< 10^{-3}$)	0.936 (0.003)	0.890 (0.018)	0.000	0.000
		CBPS-JI	$< 10^{-3}$ ($< 10^{-3}$)	0.002 ($< 10^{-3}$)	0.045 ($< 10^{-3}$)	0.035 ($< 10^{-3}$)	0.956 (0.003)	1.116 (0.023)	0.000	0.000
		CBPS-OI	-0.003 ($< 10^{-3}$)	0.002 ($< 10^{-3}$)	0.040 ($< 10^{-3}$)	0.032 ($< 10^{-3}$)	0.945 (0.003)	0.985 (0.019)	0.000	0.000
		CBPS-TLF	$< 10^{-3}$ ($< 10^{-3}$)	0.001 ($< 10^{-3}$)	0.033 ($< 10^{-3}$)	0.027 ($< 10^{-3}$)	0.929 (0.004)	0.818 (0.017)	0.000	0.000
		EB	$< 10^{-3}$ ($< 10^{-3}$)	0.002 ($< 10^{-3}$)	0.039 ($< 10^{-3}$)	0.031 ($< 10^{-3}$)	0.957 (0.003)	1.076 (0.022)	0.000	0.000
		KOM	-0.001 ($< 10^{-3}$)	0.001 ($< 10^{-3}$)	0.037 ($< 10^{-3}$)	0.029 ($< 10^{-3}$)	0.954 (0.003)	1.058 (0.022)	0.000	0.000

Table 25: Scenario: $n = 1000$, proportion treated: moderate; complexity: low; treatment-effect level: none. Values are reported as estimate (Monte Carlo standard error), except for the missingness columns, which are reported without Monte Carlo standard error.

Estimand	Estimator	Method	Bias	Var	RMSE	MAE	Coverage	Var. ratio	Missingness Point	Missingness SE
ATE	WLS	IPTW	0.005 ($< 10^{-3}$)	$< 10^{-3}$ ($< 10^{-3}$)	0.028 ($< 10^{-3}$)	0.023 ($< 10^{-3}$)	0.944 (0.003)	0.984 (0.019)	0.000	0.000
		CBPS-JI	0.004 ($< 10^{-3}$)	$< 10^{-3}$ ($< 10^{-3}$)	0.028 ($< 10^{-3}$)	0.022 ($< 10^{-3}$)	0.945 (0.003)	0.978 (0.019)	0.000	0.000
		CBPS-OI	0.008 ($< 10^{-3}$)	$< 10^{-3}$ ($< 10^{-3}$)	0.029 ($< 10^{-3}$)	0.023 ($< 10^{-3}$)	0.958 (0.003)	1.167 (0.022)	0.000	0.000
		CBPS-TLF	0.009 ($< 10^{-3}$)	$< 10^{-3}$ ($< 10^{-3}$)	0.029 ($< 10^{-3}$)	0.023 ($< 10^{-3}$)	0.883 (0.005)	0.703 (0.014)	0.000	0.000
		EB	0.001 ($< 10^{-3}$)	$< 10^{-3}$ ($< 10^{-3}$)	0.029 ($< 10^{-3}$)	0.024 ($< 10^{-3}$)	0.972 (0.002)	1.206 (0.024)	0.000	0.000
		KOM	0.005 ($< 10^{-3}$)	$< 10^{-3}$ ($< 10^{-3}$)	0.027 ($< 10^{-3}$)	0.022 ($< 10^{-3}$)	0.965 (0.003)	1.191 (0.023)	0.000	0.000
	DR	IPTW	$< 10^{-3}$ ($< 10^{-3}$)	$< 10^{-3}$ ($< 10^{-3}$)	0.027 ($< 10^{-3}$)	0.022 ($< 10^{-3}$)	0.951 (0.003)	0.980 (0.019)	0.000	0.000
		CBPS-JI	$< 10^{-3}$ ($< 10^{-3}$)	$< 10^{-3}$ ($< 10^{-3}$)	0.027 ($< 10^{-3}$)	0.022 ($< 10^{-3}$)	0.951 (0.003)	0.974 (0.019)	0.000	0.000
		CBPS-OI	$< 10^{-3}$ ($< 10^{-3}$)	$< 10^{-3}$ ($< 10^{-3}$)	0.027 ($< 10^{-3}$)	0.022 ($< 10^{-3}$)	0.951 (0.003)	0.989 (0.019)	0.000	0.000
		CBPS-TLF	$< 10^{-3}$ ($< 10^{-3}$)	$< 10^{-3}$ ($< 10^{-3}$)	0.027 ($< 10^{-3}$)	0.022 ($< 10^{-3}$)	0.911 (0.004)	0.733 (0.014)	0.000	0.000
		EB	$< 10^{-3}$ ($< 10^{-3}$)	$< 10^{-3}$ ($< 10^{-3}$)	0.027 ($< 10^{-3}$)	0.022 ($< 10^{-3}$)	0.951 (0.003)	0.989 (0.019)	0.000	0.000
		KOM	$< 10^{-3}$ ($< 10^{-3}$)	$< 10^{-3}$ ($< 10^{-3}$)	0.027 ($< 10^{-3}$)	0.022 ($< 10^{-3}$)	0.951 (0.003)	0.989 (0.019)	0.000	0.000
ATT	WLS	IPTW	0.008 ($< 10^{-3}$)	0.001 ($< 10^{-3}$)	0.034 ($< 10^{-3}$)	0.027 ($< 10^{-3}$)	0.933 (0.004)	0.986 (0.019)	0.000	0.000
		CBPS-JI	0.005 ($< 10^{-3}$)	$< 10^{-3}$ ($< 10^{-3}$)	0.032 ($< 10^{-3}$)	0.026 ($< 10^{-3}$)	0.950 (0.003)	1.034 (0.020)	0.000	0.000
		CBPS-OI	0.011 ($< 10^{-3}$)	0.001 ($< 10^{-3}$)	0.034 ($< 10^{-3}$)	0.027 ($< 10^{-3}$)	0.944 (0.003)	1.133 (0.021)	0.000	0.000
		CBPS-TLF	0.010 ($< 10^{-3}$)	$< 10^{-3}$ ($< 10^{-3}$)	0.032 ($< 10^{-3}$)	0.026 ($< 10^{-3}$)	0.912 (0.004)	0.833 (0.016)	0.000	0.000
		EB	0.004 ($< 10^{-3}$)	0.001 ($< 10^{-3}$)	0.034 ($< 10^{-3}$)	0.027 ($< 10^{-3}$)	0.966 (0.003)	1.211 (0.024)	0.000	0.000
		KOM	0.007 ($< 10^{-3}$)	$< 10^{-3}$ ($< 10^{-3}$)	0.031 ($< 10^{-3}$)	0.025 ($< 10^{-3}$)	0.963 (0.003)	1.173 (0.023)	0.000	0.000
	DR	IPTW	$< 10^{-3}$ ($< 10^{-3}$)	$< 10^{-3}$ ($< 10^{-3}$)	0.031 ($< 10^{-3}$)	0.025 ($< 10^{-3}$)	0.949 (0.003)	0.984 (0.019)	0.000	0.000
		CBPS-JI	$< 10^{-3}$ ($< 10^{-3}$)	$< 10^{-3}$ ($< 10^{-3}$)	0.031 ($< 10^{-3}$)	0.025 ($< 10^{-3}$)	0.947 (0.003)	0.973 (0.019)	0.000	0.000
		CBPS-OI	$< 10^{-3}$ ($< 10^{-3}$)	$< 10^{-3}$ ($< 10^{-3}$)	0.031 ($< 10^{-3}$)	0.025 ($< 10^{-3}$)	0.951 (0.003)	1.000 (0.020)	0.000	0.000
		CBPS-TLF	0.001 ($< 10^{-3}$)	$< 10^{-3}$ ($< 10^{-3}$)	0.031 ($< 10^{-3}$)	0.025 ($< 10^{-3}$)	0.924 (0.004)	0.818 (0.016)	0.000	0.000
		EB	$< 10^{-3}$ ($< 10^{-3}$)	0.001 ($< 10^{-3}$)	0.034 ($< 10^{-3}$)	0.027 ($< 10^{-3}$)	0.947 (0.003)	0.982 (0.020)	0.000	0.000
		KOM	$< 10^{-3}$ ($< 10^{-3}$)	$< 10^{-3}$ ($< 10^{-3}$)	0.030 ($< 10^{-3}$)	0.024 ($< 10^{-3}$)	0.952 (0.003)	0.997 (0.020)	0.000	0.000

Table 26: Scenario: $n = 1000$, proportion treated: moderate; complexity: moderate; treatment-effect level: none. Values are reported as estimate (Monte Carlo standard error), except for the missingness columns, which are reported without Monte Carlo standard error.

Estimand	Estimator	Method	Bias	Var	RMSE	MAE	Coverage	Var. ratio	Missingness Point	Missingness SE
ATE	WLS	IPTW	0.012 ($< 10^{-3}$)	0.001 ($< 10^{-3}$)	0.034 ($< 10^{-3}$)	0.027 ($< 10^{-3}$)	0.915 (0.004)	0.939 (0.019)	0.000	0.000
		CBPS-JI	0.009 ($< 10^{-3}$)	$< 10^{-3}$ ($< 10^{-3}$)	0.031 ($< 10^{-3}$)	0.025 ($< 10^{-3}$)	0.921 (0.004)	0.897 (0.019)	0.000	0.000
		CBPS-OI	0.017 ($< 10^{-3}$)	$< 10^{-3}$ ($< 10^{-3}$)	0.034 ($< 10^{-3}$)	0.028 ($< 10^{-3}$)	0.942 (0.003)	1.313 (0.027)	0.000	0.000
		CBPS-TLF	0.048 ($< 10^{-3}$)	$< 10^{-3}$ ($< 10^{-3}$)	0.055 ($< 10^{-3}$)	0.049 ($< 10^{-3}$)	0.375 (0.007)	0.661 (0.013)	0.000	0.000
		EB	0.003 ($< 10^{-3}$)	$< 10^{-3}$ ($< 10^{-3}$)	0.029 ($< 10^{-3}$)	0.023 ($< 10^{-3}$)	0.984 (0.002)	1.520 (0.031)	0.000	0.000
		KOM	0.006 ($< 10^{-3}$)	$< 10^{-3}$ ($< 10^{-3}$)	0.027 ($< 10^{-3}$)	0.022 ($< 10^{-3}$)	0.976 (0.002)	1.456 (0.029)	0.000	0.000
	DR	IPTW	$< 10^{-3}$ ($< 10^{-3}$)	$< 10^{-3}$ ($< 10^{-3}$)	0.029 ($< 10^{-3}$)	0.023 ($< 10^{-3}$)	0.938 (0.003)	0.939 (0.019)	0.000	0.000
		CBPS-JI	$< 10^{-3}$ ($< 10^{-3}$)	$< 10^{-3}$ ($< 10^{-3}$)	0.029 ($< 10^{-3}$)	0.023 ($< 10^{-3}$)	0.929 (0.004)	0.890 (0.018)	0.000	0.000
		CBPS-OI	$< 10^{-3}$ ($< 10^{-3}$)	$< 10^{-3}$ ($< 10^{-3}$)	0.026 ($< 10^{-3}$)	0.021 ($< 10^{-3}$)	0.945 (0.003)	0.976 (0.019)	0.000	0.000
		CBPS-TLF	$< 10^{-3}$ ($< 10^{-3}$)	$< 10^{-3}$ ($< 10^{-3}$)	0.026 ($< 10^{-3}$)	0.021 ($< 10^{-3}$)	0.916 (0.004)	0.780 (0.016)	0.000	0.000
		EB	$< 10^{-3}$ ($< 10^{-3}$)	$< 10^{-3}$ ($< 10^{-3}$)	0.026 ($< 10^{-3}$)	0.021 ($< 10^{-3}$)	0.945 (0.003)	0.976 (0.019)	0.000	0.000
		KOM	$< 10^{-3}$ ($< 10^{-3}$)	$< 10^{-3}$ ($< 10^{-3}$)	0.026 ($< 10^{-3}$)	0.021 ($< 10^{-3}$)	0.945 (0.003)	0.976 (0.019)	0.000	0.000
ATT	WLS	IPTW	0.020 ($< 10^{-3}$)	0.002 ($< 10^{-3}$)	0.049 ($< 10^{-3}$)	0.039 ($< 10^{-3}$)	0.877 (0.005)	0.926 (0.019)	0.000	0.000
		CBPS-JI	0.010 ($< 10^{-3}$)	0.001 ($< 10^{-3}$)	0.040 ($< 10^{-3}$)	0.032 ($< 10^{-3}$)	0.940 (0.003)	1.107 (0.023)	0.000	0.000
		CBPS-OI	0.024 ($< 10^{-3}$)	0.002 ($< 10^{-3}$)	0.047 ($< 10^{-3}$)	0.038 ($< 10^{-3}$)	0.906 (0.004)	1.193 (0.024)	0.000	0.000
		CBPS-TLF	0.058 ($< 10^{-3}$)	$< 10^{-3}$ ($< 10^{-3}$)	0.064 ($< 10^{-3}$)	0.059 ($< 10^{-3}$)	0.349 (0.007)	0.827 (0.017)	0.000	0.000
		EB	0.008 ($< 10^{-3}$)	0.001 ($< 10^{-3}$)	0.037 ($< 10^{-3}$)	0.030 ($< 10^{-3}$)	0.976 (0.002)	1.523 (0.031)	0.000	0.000
		KOM	0.008 ($< 10^{-3}$)	0.001 ($< 10^{-3}$)	0.034 ($< 10^{-3}$)	0.027 ($< 10^{-3}$)	0.972 (0.002)	1.455 (0.029)	0.000	0.000
	DR	IPTW	$< 10^{-3}$ ($< 10^{-3}$)	0.002 ($< 10^{-3}$)	0.039 ($< 10^{-3}$)	0.030 ($< 10^{-3}$)	0.934 (0.004)	0.936 (0.020)	0.000	0.000
		CBPS-JI	$< 10^{-3}$ ($< 10^{-3}$)	0.001 ($< 10^{-3}$)	0.037 ($< 10^{-3}$)	0.030 ($< 10^{-3}$)	0.926 (0.004)	0.890 (0.018)	0.000	0.000
		CBPS-OI	$< 10^{-3}$ ($< 10^{-3}$)	0.001 ($< 10^{-3}$)	0.036 ($< 10^{-3}$)	0.029 ($< 10^{-3}$)	0.939 (0.003)	0.985 (0.021)	0.000	0.000
		CBPS-TLF	$< 10^{-3}$ ($< 10^{-3}$)	0.001 ($< 10^{-3}$)	0.032 ($< 10^{-3}$)	0.026 ($< 10^{-3}$)	0.931 (0.004)	0.874 (0.018)	0.000	0.000
		EB	$< 10^{-3}$ ($< 10^{-3}$)	0.001 ($< 10^{-3}$)	0.037 ($< 10^{-3}$)	0.029 ($< 10^{-3}$)	0.945 (0.003)	0.987 (0.020)	0.000	0.000
		KOM	$< 10^{-3}$ ($< 10^{-3}$)	0.001 ($< 10^{-3}$)	0.034 ($< 10^{-3}$)	0.027 ($< 10^{-3}$)	0.944 (0.003)	0.984 (0.020)	0.000	0.000

Table 27: Scenario: $n = 1000$, proportion treated: moderate; complexity: high; treatment-effect level: none. Values are reported as estimate (Monte Carlo standard error), except for the missingness columns, which are reported without Monte Carlo standard error.

Estimand	Estimator	Method	Bias	Var	RMSE	MAE	Coverage	Var. ratio	Missingness Point	Missingness SE
ATE	WLS	IPTW	0.024 ($< 10^{-3}$)	0.003 ($< 10^{-3}$)	0.058 ($< 10^{-3}$)	0.046 ($< 10^{-3}$)	0.829 (0.005)	0.758 (0.019)	0.000	0.000
		CBPS-JI	0.018 ($< 10^{-3}$)	0.002 ($< 10^{-3}$)	0.045 ($< 10^{-3}$)	0.036 ($< 10^{-3}$)	0.828 (0.005)	0.632 (0.013)	0.000	0.000
		CBPS-OI	0.034 ($< 10^{-3}$)	0.002 ($< 10^{-3}$)	0.053 ($< 10^{-3}$)	0.044 ($< 10^{-3}$)	0.875 (0.005)	1.311 (0.027)	0.000	0.000
		CBPS-TLF	0.080 ($< 10^{-3}$)	$< 10^{-3}$ ($< 10^{-3}$)	0.084 ($< 10^{-3}$)	0.080 ($< 10^{-3}$)	0.031 (0.002)	0.524 (0.011)	0.000	0.000
		EB	0.005 ($< 10^{-3}$)	$< 10^{-3}$ ($< 10^{-3}$)	0.029 ($< 10^{-3}$)	0.023 ($< 10^{-3}$)	0.996 ($< 10^{-3}$)	2.144 (0.043)	0.000	0.000
		KOM	0.010 ($< 10^{-3}$)	$< 10^{-3}$ ($< 10^{-3}$)	0.030 ($< 10^{-3}$)	0.024 ($< 10^{-3}$)	0.989 (0.001)	1.952 (0.040)	0.000	0.000
	DR	IPTW	-0.002 ($< 10^{-3}$)	0.002 ($< 10^{-3}$)	0.043 (0.002)	0.029 ($< 10^{-3}$)	0.927 (0.004)	0.835 (0.027)	0.000	0.000
		CBPS-JI	$< 10^{-3}$ ($< 10^{-3}$)	0.001 ($< 10^{-3}$)	0.036 ($< 10^{-3}$)	0.028 ($< 10^{-3}$)	0.883 (0.005)	0.642 (0.014)	0.000	0.000
		CBPS-OI	$< 10^{-3}$ ($< 10^{-3}$)	$< 10^{-3}$ ($< 10^{-3}$)	0.025 ($< 10^{-3}$)	0.020 ($< 10^{-3}$)	0.945 (0.003)	0.977 (0.020)	0.000	0.000
		CBPS-TLF	$< 10^{-3}$ ($< 10^{-3}$)	$< 10^{-3}$ ($< 10^{-3}$)	0.025 ($< 10^{-3}$)	0.020 ($< 10^{-3}$)	0.911 (0.004)	0.771 (0.016)	0.000	0.000
		EB	$< 10^{-3}$ ($< 10^{-3}$)	$< 10^{-3}$ ($< 10^{-3}$)	0.025 ($< 10^{-3}$)	0.020 ($< 10^{-3}$)	0.945 (0.003)	0.977 (0.020)	0.000	0.000
		KOM	$< 10^{-3}$ ($< 10^{-3}$)	$< 10^{-3}$ ($< 10^{-3}$)	0.025 ($< 10^{-3}$)	0.020 ($< 10^{-3}$)	0.945 (0.003)	0.977 (0.020)	0.000	0.000
ATT	WLS	IPTW	0.037 (0.001)	0.007 ($< 10^{-3}$)	0.089 ($< 10^{-3}$)	0.072 ($< 10^{-3}$)	0.771 (0.006)	0.705 (0.018)	0.000	0.000
		CBPS-JI	0.017 ($< 10^{-3}$)	0.004 ($< 10^{-3}$)	0.062 ($< 10^{-3}$)	0.049 ($< 10^{-3}$)	0.944 (0.003)	1.847 (0.055)	0.000	0.000
		CBPS-OI	0.042 ($< 10^{-3}$)	0.004 ($< 10^{-3}$)	0.077 ($< 10^{-3}$)	0.063 ($< 10^{-3}$)	0.819 (0.005)	1.104 (0.022)	0.000	0.000
		CBPS-TLF	0.090 ($< 10^{-3}$)	$< 10^{-3}$ ($< 10^{-3}$)	0.094 ($< 10^{-3}$)	0.090 ($< 10^{-3}$)	0.041 (0.003)	0.717 (0.015)	0.000	0.000
		EB	0.021 ($< 10^{-3}$)	0.002 ($< 10^{-3}$)	0.047 ($< 10^{-3}$)	0.038 ($< 10^{-3}$)	0.976 (0.002)	1.987 (0.041)	0.000	0.000
		KOM	0.012 ($< 10^{-3}$)	0.002 ($< 10^{-3}$)	0.042 ($< 10^{-3}$)	0.033 ($< 10^{-3}$)	0.985 (0.002)	1.930 (0.040)	0.000	0.000
	DR	IPTW	-0.005 ($< 10^{-3}$)	0.004 ($< 10^{-3}$)	0.066 (0.002)	0.044 ($< 10^{-3}$)	0.924 (0.004)	0.841 (0.019)	0.000	0.000
		CBPS-JI	-0.002 ($< 10^{-3}$)	0.003 ($< 10^{-3}$)	0.054 ($< 10^{-3}$)	0.043 ($< 10^{-3}$)	0.904 (0.004)	0.911 (0.023)	0.000	0.001
		CBPS-OI	-0.003 ($< 10^{-3}$)	0.002 ($< 10^{-3}$)	0.048 ($< 10^{-3}$)	0.037 ($< 10^{-3}$)	0.932 (0.004)	0.952 (0.020)	0.000	0.000
		CBPS-TLF	-0.002 ($< 10^{-3}$)	0.001 ($< 10^{-3}$)	0.035 ($< 10^{-3}$)	0.028 ($< 10^{-3}$)	0.919 (0.004)	0.844 (0.017)	0.000	0.000
		EB	-0.001 ($< 10^{-3}$)	0.002 ($< 10^{-3}$)	0.042 ($< 10^{-3}$)	0.033 ($< 10^{-3}$)	0.940 (0.003)	0.994 (0.021)	0.000	0.000
		KOM	-0.002 ($< 10^{-3}$)	0.002 ($< 10^{-3}$)	0.040 ($< 10^{-3}$)	0.032 ($< 10^{-3}$)	0.941 (0.003)	0.996 (0.021)	0.000	0.000

Table 28: Scenario: $n = 1000$, proportion treated: high; complexity: low; treatment-effect level: none. Values are reported as estimate (Monte Carlo standard error), except for the missingness columns, which are reported without Monte Carlo standard error.

Estimand	Estimator	Method	Bias	Var	RMSE	MAE	Coverage	Var. ratio	Missingness Point	Missingness SE
ATE	WLS	IPTW	0.005 ($< 10^{-3}$)	0.001 ($< 10^{-3}$)	0.038 ($< 10^{-3}$)	0.030 ($< 10^{-3}$)	0.929 (0.004)	0.919 (0.019)	0.000	0.000
		CBPS-JI	0.003 ($< 10^{-3}$)	0.001 ($< 10^{-3}$)	0.036 ($< 10^{-3}$)	0.029 ($< 10^{-3}$)	0.928 (0.004)	0.866 (0.018)	0.000	0.000
		CBPS-OI	0.008 ($< 10^{-3}$)	0.001 ($< 10^{-3}$)	0.037 ($< 10^{-3}$)	0.029 ($< 10^{-3}$)	0.946 (0.003)	1.087 (0.022)	0.000	0.000
		CBPS-TLF	0.021 ($< 10^{-3}$)	$< 10^{-3}$ ($< 10^{-3}$)	0.038 ($< 10^{-3}$)	0.030 ($< 10^{-3}$)	0.758 (0.006)	0.543 (0.011)	0.000	0.000
		EB	0.002 ($< 10^{-3}$)	0.001 ($< 10^{-3}$)	0.037 ($< 10^{-3}$)	0.029 ($< 10^{-3}$)	0.960 (0.003)	1.171 (0.024)	0.000	0.000
		KOM	0.008 ($< 10^{-3}$)	0.001 ($< 10^{-3}$)	0.034 ($< 10^{-3}$)	0.027 ($< 10^{-3}$)	0.946 (0.003)	1.093 (0.022)	0.000	0.000
	DR	IPTW	$< 10^{-3}$ ($< 10^{-3}$)	0.001 ($< 10^{-3}$)	0.036 ($< 10^{-3}$)	0.029 ($< 10^{-3}$)	0.935 (0.003)	0.904 (0.018)	0.000	0.000
		CBPS-JI	$< 10^{-3}$ ($< 10^{-3}$)	0.001 ($< 10^{-3}$)	0.036 ($< 10^{-3}$)	0.029 ($< 10^{-3}$)	0.927 (0.004)	0.860 (0.018)	0.000	0.000
		CBPS-OI	$< 10^{-3}$ ($< 10^{-3}$)	0.001 ($< 10^{-3}$)	0.035 ($< 10^{-3}$)	0.028 ($< 10^{-3}$)	0.938 (0.003)	0.922 (0.019)	0.000	0.000
		CBPS-TLF	$< 10^{-3}$ ($< 10^{-3}$)	0.001 ($< 10^{-3}$)	0.035 ($< 10^{-3}$)	0.028 ($< 10^{-3}$)	0.856 (0.005)	0.552 (0.011)	0.000	0.000
		EB	$< 10^{-3}$ ($< 10^{-3}$)	0.001 ($< 10^{-3}$)	0.035 ($< 10^{-3}$)	0.028 ($< 10^{-3}$)	0.938 (0.003)	0.922 (0.019)	0.000	0.000
		KOM	$< 10^{-3}$ ($< 10^{-3}$)	0.001 ($< 10^{-3}$)	0.035 ($< 10^{-3}$)	0.028 ($< 10^{-3}$)	0.938 (0.003)	0.922 (0.019)	0.000	0.000
ATT	WLS	IPTW	0.007 ($< 10^{-3}$)	0.002 ($< 10^{-3}$)	0.043 ($< 10^{-3}$)	0.034 ($< 10^{-3}$)	0.924 (0.004)	0.915 (0.019)	0.000	0.000
		CBPS-JI	0.004 ($< 10^{-3}$)	0.002 ($< 10^{-3}$)	0.041 ($< 10^{-3}$)	0.032 ($< 10^{-3}$)	0.922 (0.004)	0.859 (0.018)	0.000	0.000
		CBPS-OI	0.009 ($< 10^{-3}$)	0.002 ($< 10^{-3}$)	0.041 ($< 10^{-3}$)	0.033 ($< 10^{-3}$)	0.936 (0.003)	1.062 (0.022)	0.000	0.000
		CBPS-TLF	0.024 ($< 10^{-3}$)	0.001 ($< 10^{-3}$)	0.040 ($< 10^{-3}$)	0.033 ($< 10^{-3}$)	0.803 (0.006)	0.713 (0.015)	0.000	0.000
		EB	0.004 ($< 10^{-3}$)	0.002 ($< 10^{-3}$)	0.041 ($< 10^{-3}$)	0.032 ($< 10^{-3}$)	0.954 (0.003)	1.164 (0.024)	0.000	0.000
		KOM	0.010 ($< 10^{-3}$)	0.001 ($< 10^{-3}$)	0.037 ($< 10^{-3}$)	0.030 ($< 10^{-3}$)	0.938 (0.003)	1.070 (0.022)	0.000	0.000
	DR	IPTW	$< 10^{-3}$ ($< 10^{-3}$)	0.002 ($< 10^{-3}$)	0.040 ($< 10^{-3}$)	0.032 ($< 10^{-3}$)	0.928 (0.004)	0.897 (0.018)	0.000	0.000
		CBPS-JI	$< 10^{-3}$ ($< 10^{-3}$)	0.002 ($< 10^{-3}$)	0.040 ($< 10^{-3}$)	0.032 ($< 10^{-3}$)	0.920 (0.004)	0.836 (0.017)	0.000	0.000
		CBPS-OI	$< 10^{-3}$ ($< 10^{-3}$)	0.002 ($< 10^{-3}$)	0.040 ($< 10^{-3}$)	0.032 ($< 10^{-3}$)	0.933 (0.004)	0.922 (0.019)	0.000	0.000
		CBPS-TLF	$< 10^{-3}$ ($< 10^{-3}$)	0.001 ($< 10^{-3}$)	0.038 ($< 10^{-3}$)	0.031 ($< 10^{-3}$)	0.885 (0.005)	0.650 (0.013)	0.000	0.000
		EB	$< 10^{-3}$ ($< 10^{-3}$)	0.002 ($< 10^{-3}$)	0.041 ($< 10^{-3}$)	0.033 ($< 10^{-3}$)	0.932 (0.004)	0.927 (0.019)	0.000	0.000
		KOM	$< 10^{-3}$ ($< 10^{-3}$)	0.001 ($< 10^{-3}$)	0.038 ($< 10^{-3}$)	0.031 ($< 10^{-3}$)	0.936 (0.003)	0.918 (0.019)	0.000	0.000

Table 29: Scenario: $n = 1000$, proportion treated: high; complexity: moderate; treatment-effect level: none. Values are reported as estimate (Monte Carlo standard error), except for the missingness columns, which are reported without Monte Carlo standard error.

Estimand	Estimator	Method	Bias	Var	RMSE	MAE	Coverage	Var. ratio	Missingness Point	Missingness SE
ATE	WLS	IPTW	0.012 ($< 10^{-3}$)	0.003 ($< 10^{-3}$)	0.053 ($< 10^{-3}$)	0.042 ($< 10^{-3}$)	0.879 (0.005)	0.862 (0.019)	0.000	0.000
		CBPS-JI	0.008 ($< 10^{-3}$)	0.002 ($< 10^{-3}$)	0.043 ($< 10^{-3}$)	0.035 ($< 10^{-3}$)	0.889 (0.004)	0.716 (0.015)	0.000	0.000
		CBPS-OI	0.018 ($< 10^{-3}$)	0.002 ($< 10^{-3}$)	0.047 ($< 10^{-3}$)	0.038 ($< 10^{-3}$)	0.912 (0.004)	1.181 (0.025)	0.000	0.000
		CBPS-TLF	0.043 ($< 10^{-3}$)	$< 10^{-3}$ ($< 10^{-3}$)	0.053 ($< 10^{-3}$)	0.046 ($< 10^{-3}$)	0.448 (0.007)	0.425 (0.009)	0.000	0.000
		EB	0.004 ($< 10^{-3}$)	0.001 ($< 10^{-3}$)	0.039 ($< 10^{-3}$)	0.031 ($< 10^{-3}$)	0.976 (0.002)	1.461 (0.030)	0.000	0.000
		KOM	0.011 ($< 10^{-3}$)	0.001 ($< 10^{-3}$)	0.037 ($< 10^{-3}$)	0.030 ($< 10^{-3}$)	0.956 (0.003)	1.301 (0.027)	0.000	0.000
	DR	IPTW	$< 10^{-3}$ ($< 10^{-3}$)	0.002 ($< 10^{-3}$)	0.046 (0.001)	0.036 ($< 10^{-3}$)	0.915 (0.004)	0.853 (0.017)	0.000	0.000
		CBPS-JI	$< 10^{-3}$ ($< 10^{-3}$)	0.002 ($< 10^{-3}$)	0.042 ($< 10^{-3}$)	0.034 ($< 10^{-3}$)	0.887 (0.004)	0.698 (0.014)	0.000	0.000
		CBPS-OI	$< 10^{-3}$ ($< 10^{-3}$)	0.001 ($< 10^{-3}$)	0.037 ($< 10^{-3}$)	0.030 ($< 10^{-3}$)	0.928 (0.004)	0.894 (0.018)	0.000	0.000
		CBPS-TLF	$< 10^{-3}$ ($< 10^{-3}$)	0.001 ($< 10^{-3}$)	0.037 ($< 10^{-3}$)	0.030 ($< 10^{-3}$)	0.822 (0.005)	0.495 (0.010)	0.000	0.000
		EB	$< 10^{-3}$ ($< 10^{-3}$)	0.001 ($< 10^{-3}$)	0.037 ($< 10^{-3}$)	0.030 ($< 10^{-3}$)	0.928 (0.004)	0.894 (0.018)	0.000	0.000
		KOM	$< 10^{-3}$ ($< 10^{-3}$)	0.001 ($< 10^{-3}$)	0.037 ($< 10^{-3}$)	0.030 ($< 10^{-3}$)	0.928 (0.004)	0.894 (0.018)	0.000	0.000
ATT	WLS	IPTW	0.015 ($< 10^{-3}$)	0.004 ($< 10^{-3}$)	0.065 ($< 10^{-3}$)	0.052 ($< 10^{-3}$)	0.867 (0.005)	0.840 (0.018)	0.000	0.000
		CBPS-JI	0.009 ($< 10^{-3}$)	0.003 ($< 10^{-3}$)	0.055 ($< 10^{-3}$)	0.044 ($< 10^{-3}$)	0.885 (0.005)	0.745 (0.015)	0.000	0.000
		CBPS-OI	0.020 ($< 10^{-3}$)	0.003 ($< 10^{-3}$)	0.058 ($< 10^{-3}$)	0.047 ($< 10^{-3}$)	0.897 (0.004)	1.108 (0.023)	0.000	0.000
		CBPS-TLF	0.048 ($< 10^{-3}$)	0.001 ($< 10^{-3}$)	0.058 ($< 10^{-3}$)	0.050 ($< 10^{-3}$)	0.509 (0.007)	0.587 (0.012)	0.000	0.000
		EB	0.012 ($< 10^{-3}$)	0.002 ($< 10^{-3}$)	0.047 ($< 10^{-3}$)	0.038 ($< 10^{-3}$)	0.962 (0.003)	1.403 (0.029)	0.000	0.000
		KOM	0.014 ($< 10^{-3}$)	0.002 ($< 10^{-3}$)	0.044 ($< 10^{-3}$)	0.035 ($< 10^{-3}$)	0.949 (0.003)	1.275 (0.026)	0.000	0.000
	DR	IPTW	-0.001 ($< 10^{-3}$)	0.003 ($< 10^{-3}$)	0.057 (0.001)	0.044 ($< 10^{-3}$)	0.910 (0.004)	0.844 (0.017)	0.000	0.000
		CBPS-JI	$< 10^{-3}$ ($< 10^{-3}$)	0.003 ($< 10^{-3}$)	0.052 ($< 10^{-3}$)	0.042 ($< 10^{-3}$)	0.871 (0.005)	0.656 (0.014)	0.000	0.000
		CBPS-OI	$< 10^{-3}$ ($< 10^{-3}$)	0.002 ($< 10^{-3}$)	0.049 ($< 10^{-3}$)	0.040 ($< 10^{-3}$)	0.920 (0.004)	0.902 (0.019)	0.000	0.000
		CBPS-TLF	$< 10^{-3}$ ($< 10^{-3}$)	0.002 ($< 10^{-3}$)	0.044 ($< 10^{-3}$)	0.035 ($< 10^{-3}$)	0.854 (0.005)	0.566 (0.011)	0.000	0.000
		EB	$< 10^{-3}$ ($< 10^{-3}$)	0.002 ($< 10^{-3}$)	0.048 ($< 10^{-3}$)	0.038 ($< 10^{-3}$)	0.926 (0.004)	0.907 (0.018)	0.000	0.000
		KOM	$< 10^{-3}$ ($< 10^{-3}$)	0.002 ($< 10^{-3}$)	0.045 ($< 10^{-3}$)	0.036 ($< 10^{-3}$)	0.930 (0.004)	0.901 (0.018)	0.000	0.000

Table 30: Scenario: $n = 1000$, proportion treated: high; complexity: high; treatment-effect level: none. Values are reported as estimate (Monte Carlo standard error), except for the missingness columns, which are reported without Monte Carlo standard error.

Estimand	Estimator	Method	Bias	Var	RMSE	MAE	Coverage	Var. ratio	Missingness Point	Missingness SE
ATE	WLS	IPTW	0.026 (0.001)	0.009 ($< 10^{-3}$)	0.100 (0.001)	0.078 ($< 10^{-3}$)	0.731 (0.006)	0.560 (0.017)	0.000	0.000
		CBPS-JI	0.013 ($< 10^{-3}$)	0.004 ($< 10^{-3}$)	0.062 ($< 10^{-3}$)	0.050 ($< 10^{-3}$)	0.761 (0.006)	0.433 (0.009)	0.000	0.000
		CBPS-OI	0.033 (0.001)	0.005 ($< 10^{-3}$)	0.080 ($< 10^{-3}$)	0.066 ($< 10^{-3}$)	0.792 (0.006)	0.982 (0.019)	0.000	0.000
		CBPS-TLF	0.077 ($< 10^{-3}$)	$< 10^{-3}$ ($< 10^{-3}$)	0.083 ($< 10^{-3}$)	0.077 ($< 10^{-3}$)	0.089 (0.004)	0.307 (0.006)	0.000	0.000
		EB	0.010 ($< 10^{-3}$)	0.002 ($< 10^{-3}$)	0.042 ($< 10^{-3}$)	0.034 ($< 10^{-3}$)	0.985 (0.002)	1.958 (0.040)	0.000	0.000
		KOM	0.017 ($< 10^{-3}$)	0.002 ($< 10^{-3}$)	0.046 ($< 10^{-3}$)	0.036 ($< 10^{-3}$)	0.955 (0.003)	1.591 (0.032)	0.000	0.000
	DR	IPTW	-0.002 (0.001)	0.006 ($< 10^{-3}$)	0.080 (0.003)	0.053 ($< 10^{-3}$)	0.878 (0.005)	0.740 (0.018)	0.001	0.001
		CBPS-JI	-0.001 ($< 10^{-3}$)	0.003 ($< 10^{-3}$)	0.059 ($< 10^{-3}$)	0.047 ($< 10^{-3}$)	0.778 (0.006)	0.440 (0.010)	0.000	0.000
		CBPS-OI	-0.002 ($< 10^{-3}$)	0.002 ($< 10^{-3}$)	0.044 ($< 10^{-3}$)	0.035 ($< 10^{-3}$)	0.891 (0.004)	0.816 (0.017)	0.000	0.000
		CBPS-TLF	-0.002 ($< 10^{-3}$)	0.002 ($< 10^{-3}$)	0.044 ($< 10^{-3}$)	0.035 ($< 10^{-3}$)	0.805 (0.006)	0.502 (0.010)	0.000	0.000
		EB	-0.002 ($< 10^{-3}$)	0.002 ($< 10^{-3}$)	0.044 ($< 10^{-3}$)	0.035 ($< 10^{-3}$)	0.891 (0.004)	0.817 (0.017)	0.000	0.000
		KOM	-0.002 ($< 10^{-3}$)	0.002 ($< 10^{-3}$)	0.044 ($< 10^{-3}$)	0.035 ($< 10^{-3}$)	0.891 (0.004)	0.816 (0.017)	0.000	0.000
ATT	WLS	IPTW	0.032 (0.002)	0.014 ($< 10^{-3}$)	0.124 (0.001)	0.099 (0.001)	0.718 (0.006)	0.544 (0.014)	0.000	0.000
		CBPS-JI	0.017 (0.001)	0.011 ($< 10^{-3}$)	0.107 (0.001)	0.085 ($< 10^{-3}$)	0.893 (0.005)	3.086 (0.128)	0.000	0.243
		CBPS-OI	0.029 (0.002)	0.012 ($< 10^{-3}$)	0.113 (0.001)	0.093 ($< 10^{-3}$)	0.809 (0.006)	1.106 (0.024)	0.000	0.000
		CBPS-TLF	0.080 ($< 10^{-3}$)	0.001 ($< 10^{-3}$)	0.087 ($< 10^{-3}$)	0.080 ($< 10^{-3}$)	0.158 (0.005)	0.479 (0.010)	0.000	0.000
		EB	0.031 ($< 10^{-3}$)	0.003 ($< 10^{-3}$)	0.064 ($< 10^{-3}$)	0.051 ($< 10^{-3}$)	0.944 (0.003)	1.688 (0.034)	0.000	0.000
		KOM	0.023 ($< 10^{-3}$)	0.003 ($< 10^{-3}$)	0.057 ($< 10^{-3}$)	0.046 ($< 10^{-3}$)	0.943 (0.003)	1.555 (0.031)	0.000	0.000
	DR	IPTW	-0.003 (0.001)	0.010 ($< 10^{-3}$)	0.100 (0.003)	0.067 (0.001)	0.868 (0.005)	0.730 (0.016)	0.002	0.002
		CBPS-JI	$< 10^{-3}$ (0.001)	0.009 ($< 10^{-3}$)	0.094 (0.001)	0.073 ($< 10^{-3}$)	0.865 (0.006)	3.046 (0.223)	0.000	0.260
		CBPS-OI	-0.001 (0.001)	0.007 ($< 10^{-3}$)	0.086 (0.001)	0.066 ($< 10^{-3}$)	0.882 (0.005)	0.830 (0.018)	0.000	0.000
		CBPS-TLF	-0.003 ($< 10^{-3}$)	0.003 ($< 10^{-3}$)	0.056 ($< 10^{-3}$)	0.044 ($< 10^{-3}$)	0.822 (0.005)	0.543 (0.011)	0.000	0.000
		EB	-0.001 ($< 10^{-3}$)	0.004 ($< 10^{-3}$)	0.062 ($< 10^{-3}$)	0.049 ($< 10^{-3}$)	0.888 (0.004)	0.829 (0.017)	0.000	0.000
		KOM	-0.003 ($< 10^{-3}$)	0.003 ($< 10^{-3}$)	0.059 ($< 10^{-3}$)	0.047 ($< 10^{-3}$)	0.891 (0.004)	0.828 (0.017)	0.000	0.000

Table 31: Scenario: $n = 2000$, proportion treated: low; complexity: low; treatment-effect level: none. Values are reported as estimate (Monte Carlo standard error), except for the missingness columns, which are reported without Monte Carlo standard error.

Estimand	Estimator	Method	Bias	Var	RMSE	MAE	Coverage	Var. ratio	Missingness Point	Missingness SE
ATE	WLS	IPTW	0.002 ($< 10^{-3}$)	$< 10^{-3}$ ($< 10^{-3}$)	0.023 ($< 10^{-3}$)	0.019 ($< 10^{-3}$)	0.946 (0.003)	0.987 (0.019)	0.000	0.000
		CBPS-JI	0.005 ($< 10^{-3}$)	$< 10^{-3}$ ($< 10^{-3}$)	0.024 ($< 10^{-3}$)	0.019 ($< 10^{-3}$)	0.937 (0.003)	0.955 (0.019)	0.000	0.000
		CBPS-OI	0.007 ($< 10^{-3}$)	$< 10^{-3}$ ($< 10^{-3}$)	0.024 ($< 10^{-3}$)	0.019 ($< 10^{-3}$)	0.961 (0.003)	1.164 (0.023)	0.000	0.000
		CBPS-TLF	0.006 ($< 10^{-3}$)	$< 10^{-3}$ ($< 10^{-3}$)	0.024 ($< 10^{-3}$)	0.019 ($< 10^{-3}$)	0.860 (0.005)	0.603 (0.012)	0.000	0.000
		EB	$< 10^{-3}$ ($< 10^{-3}$)	$< 10^{-3}$ ($< 10^{-3}$)	0.025 ($< 10^{-3}$)	0.020 ($< 10^{-3}$)	0.970 (0.002)	1.222 (0.024)	0.000	0.000
		KOM	0.004 ($< 10^{-3}$)	$< 10^{-3}$ ($< 10^{-3}$)	0.022 ($< 10^{-3}$)	0.018 ($< 10^{-3}$)	0.966 (0.003)	1.212 (0.024)	0.000	0.000
	DR	IPTW	$< 10^{-3}$ ($< 10^{-3}$)	$< 10^{-3}$ ($< 10^{-3}$)	0.023 ($< 10^{-3}$)	0.019 ($< 10^{-3}$)	0.942 (0.003)	0.974 (0.019)	0.000	0.000
		CBPS-JI	$< 10^{-3}$ ($< 10^{-3}$)	$< 10^{-3}$ ($< 10^{-3}$)	0.023 ($< 10^{-3}$)	0.018 ($< 10^{-3}$)	0.939 (0.003)	0.955 (0.019)	0.000	0.000
		CBPS-OI	$< 10^{-3}$ ($< 10^{-3}$)	$< 10^{-3}$ ($< 10^{-3}$)	0.022 ($< 10^{-3}$)	0.018 ($< 10^{-3}$)	0.948 (0.003)	0.988 (0.019)	0.000	0.000
		CBPS-TLF	$< 10^{-3}$ ($< 10^{-3}$)	$< 10^{-3}$ ($< 10^{-3}$)	0.023 ($< 10^{-3}$)	0.019 ($< 10^{-3}$)	0.873 (0.005)	0.604 (0.012)	0.000	0.000
		EB	$< 10^{-3}$ ($< 10^{-3}$)	$< 10^{-3}$ ($< 10^{-3}$)	0.022 ($< 10^{-3}$)	0.018 ($< 10^{-3}$)	0.948 (0.003)	0.988 (0.019)	0.000	0.000
		KOM	$< 10^{-3}$ ($< 10^{-3}$)	$< 10^{-3}$ ($< 10^{-3}$)	0.022 ($< 10^{-3}$)	0.018 ($< 10^{-3}$)	0.948 (0.003)	0.988 (0.019)	0.000	0.000
ATT	WLS	IPTW	0.010 ($< 10^{-3}$)	$< 10^{-3}$ ($< 10^{-3}$)	0.027 ($< 10^{-3}$)	0.021 ($< 10^{-3}$)	0.929 (0.004)	0.983 (0.020)	0.000	0.000
		CBPS-JI	0.006 ($< 10^{-3}$)	$< 10^{-3}$ ($< 10^{-3}$)	0.025 ($< 10^{-3}$)	0.020 ($< 10^{-3}$)	0.956 (0.003)	1.115 (0.022)	0.000	0.000
		CBPS-OI	0.015 ($< 10^{-3}$)	$< 10^{-3}$ ($< 10^{-3}$)	0.029 ($< 10^{-3}$)	0.023 ($< 10^{-3}$)	0.926 (0.004)	1.138 (0.023)	0.000	0.000
		CBPS-TLF	0.007 ($< 10^{-3}$)	$< 10^{-3}$ ($< 10^{-3}$)	0.026 ($< 10^{-3}$)	0.020 ($< 10^{-3}$)	0.930 (0.004)	0.908 (0.018)	0.000	0.000
		EB	0.002 ($< 10^{-3}$)	$< 10^{-3}$ ($< 10^{-3}$)	0.026 ($< 10^{-3}$)	0.021 ($< 10^{-3}$)	0.970 (0.002)	1.242 (0.025)	0.000	0.000
		KOM	0.004 ($< 10^{-3}$)	$< 10^{-3}$ ($< 10^{-3}$)	0.024 ($< 10^{-3}$)	0.019 ($< 10^{-3}$)	0.971 (0.002)	1.231 (0.025)	0.000	0.000
	DR	IPTW	$< 10^{-3}$ ($< 10^{-3}$)	$< 10^{-3}$ ($< 10^{-3}$)	0.024 ($< 10^{-3}$)	0.019 ($< 10^{-3}$)	0.945 (0.003)	0.977 (0.020)	0.000	0.000
		CBPS-JI	$< 10^{-3}$ ($< 10^{-3}$)	$< 10^{-3}$ ($< 10^{-3}$)	0.024 ($< 10^{-3}$)	0.019 ($< 10^{-3}$)	0.947 (0.003)	0.983 (0.020)	0.000	0.000
		CBPS-OI	$< 10^{-3}$ ($< 10^{-3}$)	$< 10^{-3}$ ($< 10^{-3}$)	0.024 ($< 10^{-3}$)	0.019 ($< 10^{-3}$)	0.946 (0.003)	0.983 (0.020)	0.000	0.000
		CBPS-TLF	$< 10^{-3}$ ($< 10^{-3}$)	$< 10^{-3}$ ($< 10^{-3}$)	0.025 ($< 10^{-3}$)	0.020 ($< 10^{-3}$)	0.932 (0.004)	0.906 (0.018)	0.000	0.000
		EB	$< 10^{-3}$ ($< 10^{-3}$)	$< 10^{-3}$ ($< 10^{-3}$)	0.026 ($< 10^{-3}$)	0.021 ($< 10^{-3}$)	0.947 (0.003)	0.987 (0.020)	0.000	0.000
		KOM	$< 10^{-3}$ ($< 10^{-3}$)	$< 10^{-3}$ ($< 10^{-3}$)	0.023 ($< 10^{-3}$)	0.019 ($< 10^{-3}$)	0.950 (0.003)	0.998 (0.020)	0.000	0.000

Table 32: Scenario: $n = 2000$, proportion treated: low; complexity: moderate; treatment-effect level: none. Values are reported as estimate (Monte Carlo standard error), except for the missingness columns, which are reported without Monte Carlo standard error.

Estimand	Estimator	Method	Bias	Var	RMSE	MAE	Coverage	Var. ratio	Missingness	
									Point	SE
ATE	WLS	IPTW	0.005 ($< 10^{-3}$)	$< 10^{-3}$ ($< 10^{-3}$)	0.028 ($< 10^{-3}$)	0.022 ($< 10^{-3}$)	0.945 (0.003)	0.938 (0.019)	0.000	0.000
		CBPS-JI	0.013 ($< 10^{-3}$)	$< 10^{-3}$ ($< 10^{-3}$)	0.029 ($< 10^{-3}$)	0.023 ($< 10^{-3}$)	0.913 (0.004)	0.868 (0.018)	0.000	0.000
		CBPS-OI	0.016 ($< 10^{-3}$)	$< 10^{-3}$ ($< 10^{-3}$)	0.031 ($< 10^{-3}$)	0.025 ($< 10^{-3}$)	0.954 (0.003)	1.289 (0.026)	0.000	0.000
		CBPS-TLF	0.050 ($< 10^{-3}$)	$< 10^{-3}$ ($< 10^{-3}$)	0.054 ($< 10^{-3}$)	0.050 ($< 10^{-3}$)	0.181 (0.005)	0.593 (0.012)	0.000	0.000
		EB	0.002 ($< 10^{-3}$)	$< 10^{-3}$ ($< 10^{-3}$)	0.026 ($< 10^{-3}$)	0.021 ($< 10^{-3}$)	0.983 (0.002)	1.510 (0.030)	0.000	0.000
		KOM	0.006 ($< 10^{-3}$)	$< 10^{-3}$ ($< 10^{-3}$)	0.024 ($< 10^{-3}$)	0.019 ($< 10^{-3}$)	0.982 (0.002)	1.463 (0.029)	0.000	0.000
	DR	IPTW	$< 10^{-3}$ ($< 10^{-3}$)	$< 10^{-3}$ ($< 10^{-3}$)	0.026 ($< 10^{-3}$)	0.020 ($< 10^{-3}$)	0.933 (0.004)	0.955 (0.020)	0.000	0.000
		CBPS-JI	$< 10^{-3}$ ($< 10^{-3}$)	$< 10^{-3}$ ($< 10^{-3}$)	0.026 ($< 10^{-3}$)	0.021 ($< 10^{-3}$)	0.924 (0.004)	0.855 (0.017)	0.000	0.000
		CBPS-OI	$< 10^{-3}$ ($< 10^{-3}$)	$< 10^{-3}$ ($< 10^{-3}$)	0.022 ($< 10^{-3}$)	0.018 ($< 10^{-3}$)	0.942 (0.003)	0.957 (0.019)	0.000	0.000
		CBPS-TLF	$< 10^{-3}$ ($< 10^{-3}$)	$< 10^{-3}$ ($< 10^{-3}$)	0.023 ($< 10^{-3}$)	0.018 ($< 10^{-3}$)	0.890 (0.004)	0.660 (0.013)	0.000	0.000
		EB	$< 10^{-3}$ ($< 10^{-3}$)	$< 10^{-3}$ ($< 10^{-3}$)	0.022 ($< 10^{-3}$)	0.018 ($< 10^{-3}$)	0.942 (0.003)	0.957 (0.019)	0.000	0.000
		KOM	$< 10^{-3}$ ($< 10^{-3}$)	$< 10^{-3}$ ($< 10^{-3}$)	0.022 ($< 10^{-3}$)	0.018 ($< 10^{-3}$)	0.942 (0.003)	0.957 (0.019)	0.000	0.000
ATT	WLS	IPTW	0.026 ($< 10^{-3}$)	$< 10^{-3}$ ($< 10^{-3}$)	0.039 ($< 10^{-3}$)	0.032 ($< 10^{-3}$)	0.834 (0.005)	0.990 (0.021)	0.000	0.000
		CBPS-JI	0.013 ($< 10^{-3}$)	$< 10^{-3}$ ($< 10^{-3}$)	0.030 ($< 10^{-3}$)	0.024 ($< 10^{-3}$)	0.967 (0.003)	1.557 (0.032)	0.000	0.000
		CBPS-OI	0.033 ($< 10^{-3}$)	$< 10^{-3}$ ($< 10^{-3}$)	0.043 ($< 10^{-3}$)	0.036 ($< 10^{-3}$)	0.842 (0.005)	1.293 (0.026)	0.000	0.000
		CBPS-TLF	0.035 ($< 10^{-3}$)	$< 10^{-3}$ ($< 10^{-3}$)	0.042 ($< 10^{-3}$)	0.037 ($< 10^{-3}$)	0.619 (0.007)	0.893 (0.018)	0.000	0.000
		EB	0.002 ($< 10^{-3}$)	$< 10^{-3}$ ($< 10^{-3}$)	0.027 ($< 10^{-3}$)	0.022 ($< 10^{-3}$)	0.988 (0.002)	1.690 (0.034)	0.000	0.000
		KOM	0.003 ($< 10^{-3}$)	$< 10^{-3}$ ($< 10^{-3}$)	0.025 ($< 10^{-3}$)	0.020 ($< 10^{-3}$)	0.987 (0.002)	1.631 (0.033)	0.000	0.000
	DR	IPTW	-0.002 ($< 10^{-3}$)	$< 10^{-3}$ ($< 10^{-3}$)	0.026 ($< 10^{-3}$)	0.021 ($< 10^{-3}$)	0.947 (0.003)	0.983 (0.020)	0.000	0.000
		CBPS-JI	-0.001 ($< 10^{-3}$)	$< 10^{-3}$ ($< 10^{-3}$)	0.026 ($< 10^{-3}$)	0.020 ($< 10^{-3}$)	0.952 (0.003)	1.008 (0.021)	0.000	0.000
		CBPS-OI	-0.002 ($< 10^{-3}$)	$< 10^{-3}$ ($< 10^{-3}$)	0.025 ($< 10^{-3}$)	0.020 ($< 10^{-3}$)	0.949 (0.003)	1.001 (0.021)	0.000	0.000
		CBPS-TLF	$< 10^{-3}$ ($< 10^{-3}$)	$< 10^{-3}$ ($< 10^{-3}$)	0.024 ($< 10^{-3}$)	0.019 ($< 10^{-3}$)	0.940 (0.003)	0.918 (0.019)	0.000	0.000
		EB	-0.001 ($< 10^{-3}$)	$< 10^{-3}$ ($< 10^{-3}$)	0.027 ($< 10^{-3}$)	0.022 ($< 10^{-3}$)	0.956 (0.003)	1.063 (0.021)	0.000	0.000
		KOM	$< 10^{-3}$ ($< 10^{-3}$)	$< 10^{-3}$ ($< 10^{-3}$)	0.024 ($< 10^{-3}$)	0.019 ($< 10^{-3}$)	0.954 (0.003)	1.039 (0.021)	0.000	0.000

Table 33: Scenario: $n = 2000$, proportion treated: low; complexity: high; treatment-effect level: none. Values are reported as estimate (Monte Carlo standard error), except for the missingness columns, which are reported without Monte Carlo standard error.

Estimand	Estimator	Method	Bias	Var	RMSE	MAE	Coverage	Var. ratio	Missingness Point	Missingness SE
ATE	WLS	IPTW	0.007 ($< 10^{-3}$)	0.002 ($< 10^{-3}$)	0.045 ($< 10^{-3}$)	0.035 ($< 10^{-3}$)	0.921 (0.004)	0.777 (0.016)	0.000	0.000
		CBPS-JI	0.032 ($< 10^{-3}$)	0.001 ($< 10^{-3}$)	0.049 ($< 10^{-3}$)	0.039 ($< 10^{-3}$)	0.741 (0.006)	0.619 (0.013)	0.000	0.000
		CBPS-OI	0.039 ($< 10^{-3}$)	0.001 ($< 10^{-3}$)	0.053 ($< 10^{-3}$)	0.043 ($< 10^{-3}$)	0.909 (0.004)	1.377 (0.027)	0.000	0.000
		CBPS-TLF	0.088 ($< 10^{-3}$)	$< 10^{-3}$ ($< 10^{-3}$)	0.090 ($< 10^{-3}$)	0.088 ($< 10^{-3}$)	$< 10^{-3}$ ($< 10^{-3}$)	0.457 (0.009)	0.000	0.000
		EB	0.007 ($< 10^{-3}$)	$< 10^{-3}$ ($< 10^{-3}$)	0.029 ($< 10^{-3}$)	0.023 ($< 10^{-3}$)	0.996 ($< 10^{-3}$)	2.135 (0.044)	0.000	0.000
		KOM	0.014 ($< 10^{-3}$)	$< 10^{-3}$ ($< 10^{-3}$)	0.030 ($< 10^{-3}$)	0.024 ($< 10^{-3}$)	0.991 (0.001)	1.953 (0.040)	0.000	0.000
	DR	IPTW	0.001 ($< 10^{-3}$)	0.001 ($< 10^{-3}$)	0.032 ($< 10^{-3}$)	0.023 ($< 10^{-3}$)	0.929 (0.004)	0.897 (0.019)	0.000	0.000
		CBPS-JI	0.002 ($< 10^{-3}$)	0.001 ($< 10^{-3}$)	0.033 ($< 10^{-3}$)	0.025 ($< 10^{-3}$)	0.884 (0.005)	0.647 (0.015)	0.000	0.000
		CBPS-OI	0.003 ($< 10^{-3}$)	$< 10^{-3}$ ($< 10^{-3}$)	0.022 ($< 10^{-3}$)	0.018 ($< 10^{-3}$)	0.944 (0.003)	0.962 (0.019)	0.000	0.000
		CBPS-TLF	0.003 ($< 10^{-3}$)	$< 10^{-3}$ ($< 10^{-3}$)	0.023 ($< 10^{-3}$)	0.018 ($< 10^{-3}$)	0.884 (0.005)	0.648 (0.013)	0.000	0.000
		EB	0.003 ($< 10^{-3}$)	$< 10^{-3}$ ($< 10^{-3}$)	0.022 ($< 10^{-3}$)	0.018 ($< 10^{-3}$)	0.944 (0.003)	0.962 (0.019)	0.000	0.000
		KOM	0.003 ($< 10^{-3}$)	$< 10^{-3}$ ($< 10^{-3}$)	0.022 ($< 10^{-3}$)	0.018 ($< 10^{-3}$)	0.944 (0.003)	0.962 (0.019)	0.000	0.000
ATT	WLS	IPTW	0.053 ($< 10^{-3}$)	0.002 ($< 10^{-3}$)	0.069 ($< 10^{-3}$)	0.059 ($< 10^{-3}$)	0.650 (0.007)	0.879 (0.020)	0.000	0.000
		CBPS-JI	0.023 ($< 10^{-3}$)	0.001 ($< 10^{-3}$)	0.043 ($< 10^{-3}$)	0.035 ($< 10^{-3}$)	0.988 (0.002)	3.024 (0.063)	0.000	0.000
		CBPS-OI	0.059 ($< 10^{-3}$)	0.002 ($< 10^{-3}$)	0.070 ($< 10^{-3}$)	0.061 ($< 10^{-3}$)	0.696 (0.007)	1.267 (0.026)	0.000	0.000
		CBPS-TLF	0.056 ($< 10^{-3}$)	$< 10^{-3}$ ($< 10^{-3}$)	0.061 ($< 10^{-3}$)	0.056 ($< 10^{-3}$)	0.226 (0.006)	0.699 (0.014)	0.000	0.000
		EB	0.010 ($< 10^{-3}$)	$< 10^{-3}$ ($< 10^{-3}$)	0.032 ($< 10^{-3}$)	0.026 ($< 10^{-3}$)	0.993 (0.001)	2.270 (0.046)	0.000	0.000
		KOM	0.007 ($< 10^{-3}$)	$< 10^{-3}$ ($< 10^{-3}$)	0.029 ($< 10^{-3}$)	0.023 ($< 10^{-3}$)	0.995 ($< 10^{-3}$)	2.196 (0.044)	0.000	0.000
	DR	IPTW	-0.004 ($< 10^{-3}$)	0.001 ($< 10^{-3}$)	0.034 ($< 10^{-3}$)	0.025 ($< 10^{-3}$)	0.945 (0.003)	0.955 (0.020)	0.000	0.000
		CBPS-JI	-0.002 ($< 10^{-3}$)	0.001 ($< 10^{-3}$)	0.032 ($< 10^{-3}$)	0.026 ($< 10^{-3}$)	0.957 (0.003)	1.089 (0.022)	0.000	0.000
		CBPS-OI	-0.004 ($< 10^{-3}$)	$< 10^{-3}$ ($< 10^{-3}$)	0.030 ($< 10^{-3}$)	0.023 ($< 10^{-3}$)	0.949 (0.003)	1.014 (0.020)	0.000	0.000
		CBPS-TLF	$< 10^{-3}$ ($< 10^{-3}$)	$< 10^{-3}$ ($< 10^{-3}$)	0.024 ($< 10^{-3}$)	0.019 ($< 10^{-3}$)	0.908 (0.004)	0.742 (0.015)	0.000	0.000
		EB	-0.002 ($< 10^{-3}$)	$< 10^{-3}$ ($< 10^{-3}$)	0.030 ($< 10^{-3}$)	0.024 ($< 10^{-3}$)	0.953 (0.003)	1.086 (0.022)	0.000	0.000
		KOM	-0.001 ($< 10^{-3}$)	$< 10^{-3}$ ($< 10^{-3}$)	0.027 ($< 10^{-3}$)	0.022 ($< 10^{-3}$)	0.952 (0.003)	1.074 (0.022)	0.000	0.000

Table 34: Scenario: $n = 2000$, proportion treated: moderate; complexity: low; treatment-effect level: none. Values are reported as estimate (Monte Carlo standard error), except for the missingness columns, which are reported without Monte Carlo standard error.

Estimand	Estimator	Method	Bias	Var	RMSE	MAE	Coverage	Var. ratio	Missingness Point	Missingness SE
ATE	WLS	IPTW	0.005 ($< 10^{-3}$)	$< 10^{-3}$ ($< 10^{-3}$)	0.020 ($< 10^{-3}$)	0.016 ($< 10^{-3}$)	0.939 (0.003)	0.985 (0.020)	0.000	0.000
		CBPS-JI	0.004 ($< 10^{-3}$)	$< 10^{-3}$ ($< 10^{-3}$)	0.020 ($< 10^{-3}$)	0.016 ($< 10^{-3}$)	0.939 (0.003)	0.979 (0.020)	0.000	0.000
		CBPS-OI	0.007 ($< 10^{-3}$)	$< 10^{-3}$ ($< 10^{-3}$)	0.021 ($< 10^{-3}$)	0.016 ($< 10^{-3}$)	0.952 (0.003)	1.159 (0.023)	0.000	0.000
		CBPS-TLF	0.010 ($< 10^{-3}$)	$< 10^{-3}$ ($< 10^{-3}$)	0.022 ($< 10^{-3}$)	0.018 ($< 10^{-3}$)	0.876 (0.005)	0.793 (0.016)	0.000	0.000
		EB	$< 10^{-3}$ ($< 10^{-3}$)	$< 10^{-3}$ ($< 10^{-3}$)	0.021 ($< 10^{-3}$)	0.016 ($< 10^{-3}$)	0.969 (0.002)	1.235 (0.025)	0.000	0.000
		KOM	0.003 ($< 10^{-3}$)	$< 10^{-3}$ ($< 10^{-3}$)	0.019 ($< 10^{-3}$)	0.015 ($< 10^{-3}$)	0.966 (0.003)	1.212 (0.024)	0.000	0.000
	DR	IPTW	$< 10^{-3}$ ($< 10^{-3}$)	$< 10^{-3}$ ($< 10^{-3}$)	0.019 ($< 10^{-3}$)	0.015 ($< 10^{-3}$)	0.946 (0.003)	0.980 (0.020)	0.000	0.000
		CBPS-JI	$< 10^{-3}$ ($< 10^{-3}$)	$< 10^{-3}$ ($< 10^{-3}$)	0.019 ($< 10^{-3}$)	0.015 ($< 10^{-3}$)	0.947 (0.003)	0.976 (0.020)	0.000	0.000
		CBPS-OI	$< 10^{-3}$ ($< 10^{-3}$)	$< 10^{-3}$ ($< 10^{-3}$)	0.019 ($< 10^{-3}$)	0.015 ($< 10^{-3}$)	0.948 (0.003)	0.998 (0.020)	0.000	0.000
		CBPS-TLF	$< 10^{-3}$ ($< 10^{-3}$)	$< 10^{-3}$ ($< 10^{-3}$)	0.019 ($< 10^{-3}$)	0.015 ($< 10^{-3}$)	0.925 (0.004)	0.832 (0.017)	0.000	0.000
		EB	$< 10^{-3}$ ($< 10^{-3}$)	$< 10^{-3}$ ($< 10^{-3}$)	0.019 ($< 10^{-3}$)	0.015 ($< 10^{-3}$)	0.948 (0.003)	0.998 (0.020)	0.000	0.000
		KOM	$< 10^{-3}$ ($< 10^{-3}$)	$< 10^{-3}$ ($< 10^{-3}$)	0.019 ($< 10^{-3}$)	0.015 ($< 10^{-3}$)	0.948 (0.003)	0.998 (0.020)	0.000	0.000
ATT	WLS	IPTW	0.008 ($< 10^{-3}$)	$< 10^{-3}$ ($< 10^{-3}$)	0.024 ($< 10^{-3}$)	0.020 ($< 10^{-3}$)	0.926 (0.004)	0.976 (0.019)	0.000	0.000
		CBPS-JI	0.005 ($< 10^{-3}$)	$< 10^{-3}$ ($< 10^{-3}$)	0.023 ($< 10^{-3}$)	0.018 ($< 10^{-3}$)	0.945 (0.003)	1.027 (0.021)	0.000	0.000
		CBPS-OI	0.011 ($< 10^{-3}$)	$< 10^{-3}$ ($< 10^{-3}$)	0.025 ($< 10^{-3}$)	0.020 ($< 10^{-3}$)	0.932 (0.004)	1.108 (0.022)	0.000	0.000
		CBPS-TLF	0.010 ($< 10^{-3}$)	$< 10^{-3}$ ($< 10^{-3}$)	0.024 ($< 10^{-3}$)	0.019 ($< 10^{-3}$)	0.891 (0.004)	0.811 (0.016)	0.000	0.000
		EB	0.002 ($< 10^{-3}$)	$< 10^{-3}$ ($< 10^{-3}$)	0.024 ($< 10^{-3}$)	0.019 ($< 10^{-3}$)	0.968 (0.002)	1.245 (0.024)	0.000	0.000
		KOM	0.004 ($< 10^{-3}$)	$< 10^{-3}$ ($< 10^{-3}$)	0.022 ($< 10^{-3}$)	0.017 ($< 10^{-3}$)	0.966 (0.003)	1.202 (0.024)	0.000	0.000
	DR	IPTW	$< 10^{-3}$ ($< 10^{-3}$)	$< 10^{-3}$ ($< 10^{-3}$)	0.022 ($< 10^{-3}$)	0.018 ($< 10^{-3}$)	0.943 (0.003)	0.978 (0.019)	0.000	0.000
		CBPS-JI	$< 10^{-3}$ ($< 10^{-3}$)	$< 10^{-3}$ ($< 10^{-3}$)	0.022 ($< 10^{-3}$)	0.018 ($< 10^{-3}$)	0.944 (0.003)	0.972 (0.019)	0.000	0.000
		CBPS-OI	$< 10^{-3}$ ($< 10^{-3}$)	$< 10^{-3}$ ($< 10^{-3}$)	0.022 ($< 10^{-3}$)	0.018 ($< 10^{-3}$)	0.946 (0.003)	0.987 (0.020)	0.000	0.000
		CBPS-TLF	$< 10^{-3}$ ($< 10^{-3}$)	$< 10^{-3}$ ($< 10^{-3}$)	0.022 ($< 10^{-3}$)	0.018 ($< 10^{-3}$)	0.922 (0.004)	0.822 (0.016)	0.000	0.000
		EB	$< 10^{-3}$ ($< 10^{-3}$)	$< 10^{-3}$ ($< 10^{-3}$)	0.024 ($< 10^{-3}$)	0.019 ($< 10^{-3}$)	0.949 (0.003)	1.004 (0.020)	0.000	0.000
		KOM	$< 10^{-3}$ ($< 10^{-3}$)	$< 10^{-3}$ ($< 10^{-3}$)	0.021 ($< 10^{-3}$)	0.017 ($< 10^{-3}$)	0.948 (0.003)	0.996 (0.020)	0.000	0.000

Table 35: Scenario: $n = 2000$, proportion treated: moderate; complexity: moderate; treatment-effect level: none. Values are reported as estimate (Monte Carlo standard error), except for the missingness columns, which are reported without Monte Carlo standard error.

Estimand	Estimator	Method	Bias	Var	RMSE	MAE	Coverage	Var. ratio	Missingness Point	Missingness SE
ATE	WLS	IPTW	0.012 ($< 10^{-3}$)	$< 10^{-3}$ ($< 10^{-3}$)	0.026 ($< 10^{-3}$)	0.021 ($< 10^{-3}$)	0.896 (0.004)	0.951 (0.019)	0.000	0.000
		CBPS-JI	0.009 ($< 10^{-3}$)	$< 10^{-3}$ ($< 10^{-3}$)	0.023 ($< 10^{-3}$)	0.019 ($< 10^{-3}$)	0.917 (0.004)	0.915 (0.018)	0.000	0.000
		CBPS-OI	0.015 ($< 10^{-3}$)	$< 10^{-3}$ ($< 10^{-3}$)	0.027 ($< 10^{-3}$)	0.021 ($< 10^{-3}$)	0.924 (0.004)	1.285 (0.026)	0.000	0.000
		CBPS-TLF	0.050 ($< 10^{-3}$)	$< 10^{-3}$ ($< 10^{-3}$)	0.053 ($< 10^{-3}$)	0.050 ($< 10^{-3}$)	0.134 (0.005)	0.669 (0.013)	0.000	0.000
		EB	0.001 ($< 10^{-3}$)	$< 10^{-3}$ ($< 10^{-3}$)	0.021 ($< 10^{-3}$)	0.017 ($< 10^{-3}$)	0.985 (0.002)	1.527 (0.031)	0.000	0.000
		KOM	0.004 ($< 10^{-3}$)	$< 10^{-3}$ ($< 10^{-3}$)	0.020 ($< 10^{-3}$)	0.016 ($< 10^{-3}$)	0.979 (0.002)	1.463 (0.029)	0.000	0.000
	DR	IPTW	$< 10^{-3}$ ($< 10^{-3}$)	$< 10^{-3}$ ($< 10^{-3}$)	0.021 ($< 10^{-3}$)	0.017 ($< 10^{-3}$)	0.943 (0.003)	0.946 (0.019)	0.000	0.000
		CBPS-JI	$< 10^{-3}$ ($< 10^{-3}$)	$< 10^{-3}$ ($< 10^{-3}$)	0.021 ($< 10^{-3}$)	0.016 ($< 10^{-3}$)	0.939 (0.003)	0.910 (0.018)	0.000	0.000
		CBPS-OI	$< 10^{-3}$ ($< 10^{-3}$)	$< 10^{-3}$ ($< 10^{-3}$)	0.018 ($< 10^{-3}$)	0.015 ($< 10^{-3}$)	0.949 (0.003)	0.968 (0.019)	0.000	0.000
		CBPS-TLF	$< 10^{-3}$ ($< 10^{-3}$)	$< 10^{-3}$ ($< 10^{-3}$)	0.019 ($< 10^{-3}$)	0.015 ($< 10^{-3}$)	0.927 (0.004)	0.834 (0.017)	0.000	0.000
		EB	$< 10^{-3}$ ($< 10^{-3}$)	$< 10^{-3}$ ($< 10^{-3}$)	0.018 ($< 10^{-3}$)	0.015 ($< 10^{-3}$)	0.949 (0.003)	0.968 (0.019)	0.000	0.000
		KOM	$< 10^{-3}$ ($< 10^{-3}$)	$< 10^{-3}$ ($< 10^{-3}$)	0.018 ($< 10^{-3}$)	0.015 ($< 10^{-3}$)	0.949 (0.003)	0.968 (0.019)	0.000	0.000
ATT	WLS	IPTW	0.020 ($< 10^{-3}$)	$< 10^{-3}$ ($< 10^{-3}$)	0.037 ($< 10^{-3}$)	0.031 ($< 10^{-3}$)	0.857 (0.005)	0.947 (0.019)	0.000	0.000
		CBPS-JI	0.010 ($< 10^{-3}$)	$< 10^{-3}$ ($< 10^{-3}$)	0.030 ($< 10^{-3}$)	0.024 ($< 10^{-3}$)	0.937 (0.003)	1.113 (0.022)	0.000	0.000
		CBPS-OI	0.024 ($< 10^{-3}$)	$< 10^{-3}$ ($< 10^{-3}$)	0.038 ($< 10^{-3}$)	0.031 ($< 10^{-3}$)	0.872 (0.005)	1.168 (0.023)	0.000	0.000
		CBPS-TLF	0.058 ($< 10^{-3}$)	$< 10^{-3}$ ($< 10^{-3}$)	0.061 ($< 10^{-3}$)	0.058 ($< 10^{-3}$)	0.097 (0.004)	0.764 (0.015)	0.000	0.000
		EB	0.004 ($< 10^{-3}$)	$< 10^{-3}$ ($< 10^{-3}$)	0.027 ($< 10^{-3}$)	0.022 ($< 10^{-3}$)	0.983 (0.002)	1.546 (0.031)	0.000	0.000
		KOM	0.004 ($< 10^{-3}$)	$< 10^{-3}$ ($< 10^{-3}$)	0.024 ($< 10^{-3}$)	0.020 ($< 10^{-3}$)	0.980 (0.002)	1.486 (0.030)	0.000	0.000
	DR	IPTW	$< 10^{-3}$ ($< 10^{-3}$)	$< 10^{-3}$ ($< 10^{-3}$)	0.027 ($< 10^{-3}$)	0.022 ($< 10^{-3}$)	0.938 (0.003)	0.952 (0.019)	0.000	0.000
		CBPS-JI	$< 10^{-3}$ ($< 10^{-3}$)	$< 10^{-3}$ ($< 10^{-3}$)	0.027 ($< 10^{-3}$)	0.021 ($< 10^{-3}$)	0.933 (0.004)	0.913 (0.019)	0.000	0.000
		CBPS-OI	$< 10^{-3}$ ($< 10^{-3}$)	$< 10^{-3}$ ($< 10^{-3}$)	0.026 ($< 10^{-3}$)	0.021 ($< 10^{-3}$)	0.941 (0.003)	0.974 (0.020)	0.000	0.000
		CBPS-TLF	0.001 ($< 10^{-3}$)	$< 10^{-3}$ ($< 10^{-3}$)	0.023 ($< 10^{-3}$)	0.018 ($< 10^{-3}$)	0.934 (0.004)	0.873 (0.018)	0.000	0.000
		EB	$< 10^{-3}$ ($< 10^{-3}$)	$< 10^{-3}$ ($< 10^{-3}$)	0.027 ($< 10^{-3}$)	0.021 ($< 10^{-3}$)	0.952 (0.003)	1.004 (0.020)	0.000	0.000
		KOM	$< 10^{-3}$ ($< 10^{-3}$)	$< 10^{-3}$ ($< 10^{-3}$)	0.024 ($< 10^{-3}$)	0.019 ($< 10^{-3}$)	0.950 (0.003)	0.993 (0.020)	0.000	0.000

Table 36: Scenario: $n = 2000$, proportion treated: moderate; complexity: high; treatment-effect level: none. Values are reported as estimate (Monte Carlo standard error), except for the missingness columns, which are reported without Monte Carlo standard error.

Estimand	Estimator	Method	Bias	Var	RMSE	MAE	Coverage	Var. ratio	Missingness Point	Missingness SE
ATE	WLS	IPTW	0.024 ($< 10^{-3}$)	0.002 ($< 10^{-3}$)	0.046 ($< 10^{-3}$)	0.036 ($< 10^{-3}$)	0.800 (0.006)	0.812 (0.029)	0.000	0.000
		CBPS-JI	0.018 ($< 10^{-3}$)	$< 10^{-3}$ ($< 10^{-3}$)	0.035 ($< 10^{-3}$)	0.028 ($< 10^{-3}$)	0.826 (0.005)	0.737 (0.014)	0.000	0.000
		CBPS-OI	0.031 ($< 10^{-3}$)	$< 10^{-3}$ ($< 10^{-3}$)	0.044 ($< 10^{-3}$)	0.037 ($< 10^{-3}$)	0.832 (0.005)	1.297 (0.025)	0.000	0.000
		CBPS-TLF	0.084 ($< 10^{-3}$)	$< 10^{-3}$ ($< 10^{-3}$)	0.086 ($< 10^{-3}$)	0.084 ($< 10^{-3}$)	0.001 ($< 10^{-3}$)	0.492 (0.010)	0.000	0.000
		EB	0.002 ($< 10^{-3}$)	$< 10^{-3}$ ($< 10^{-3}$)	0.021 ($< 10^{-3}$)	0.017 ($< 10^{-3}$)	0.996 ($< 10^{-3}$)	2.248 (0.045)	0.000	0.000
		KOM	0.007 ($< 10^{-3}$)	$< 10^{-3}$ ($< 10^{-3}$)	0.021 ($< 10^{-3}$)	0.017 ($< 10^{-3}$)	0.991 (0.001)	2.090 (0.042)	0.000	0.000
	DR	IPTW	-0.001 ($< 10^{-3}$)	$< 10^{-3}$ ($< 10^{-3}$)	0.028 ($< 10^{-3}$)	0.021 ($< 10^{-3}$)	0.945 (0.003)	0.924 (0.019)	0.000	0.000
		CBPS-JI	-0.001 ($< 10^{-3}$)	$< 10^{-3}$ ($< 10^{-3}$)	0.026 ($< 10^{-3}$)	0.020 ($< 10^{-3}$)	0.909 (0.004)	0.743 (0.015)	0.000	0.000
		CBPS-OI	$< 10^{-3}$ ($< 10^{-3}$)	$< 10^{-3}$ ($< 10^{-3}$)	0.017 ($< 10^{-3}$)	0.014 ($< 10^{-3}$)	0.954 (0.003)	1.035 (0.021)	0.000	0.000
		CBPS-TLF	$< 10^{-3}$ ($< 10^{-3}$)	$< 10^{-3}$ ($< 10^{-3}$)	0.017 ($< 10^{-3}$)	0.014 ($< 10^{-3}$)	0.934 (0.004)	0.878 (0.017)	0.000	0.000
		EB	$< 10^{-3}$ ($< 10^{-3}$)	$< 10^{-3}$ ($< 10^{-3}$)	0.017 ($< 10^{-3}$)	0.014 ($< 10^{-3}$)	0.954 (0.003)	1.035 (0.021)	0.000	0.000
		KOM	$< 10^{-3}$ ($< 10^{-3}$)	$< 10^{-3}$ ($< 10^{-3}$)	0.017 ($< 10^{-3}$)	0.014 ($< 10^{-3}$)	0.954 (0.003)	1.035 (0.021)	0.000	0.000
ATT	WLS	IPTW	0.039 ($< 10^{-3}$)	0.004 ($< 10^{-3}$)	0.072 ($< 10^{-3}$)	0.059 ($< 10^{-3}$)	0.744 (0.006)	0.762 (0.022)	0.000	0.000
		CBPS-JI	0.018 ($< 10^{-3}$)	0.002 ($< 10^{-3}$)	0.046 ($< 10^{-3}$)	0.037 ($< 10^{-3}$)	0.940 (0.003)	1.522 (0.031)	0.000	0.000
		CBPS-OI	0.042 ($< 10^{-3}$)	0.002 ($< 10^{-3}$)	0.065 ($< 10^{-3}$)	0.055 ($< 10^{-3}$)	0.778 (0.006)	1.083 (0.020)	0.000	0.000
		CBPS-TLF	0.090 ($< 10^{-3}$)	$< 10^{-3}$ ($< 10^{-3}$)	0.092 ($< 10^{-3}$)	0.090 ($< 10^{-3}$)	0.001 ($< 10^{-3}$)	0.679 (0.014)	0.000	0.000
		EB	0.012 ($< 10^{-3}$)	0.001 ($< 10^{-3}$)	0.034 ($< 10^{-3}$)	0.027 ($< 10^{-3}$)	0.987 (0.002)	2.125 (0.042)	0.000	0.000
		KOM	0.006 ($< 10^{-3}$)	$< 10^{-3}$ ($< 10^{-3}$)	0.031 ($< 10^{-3}$)	0.025 ($< 10^{-3}$)	0.991 (0.001)	2.062 (0.041)	0.000	0.000
	DR	IPTW	-0.004 ($< 10^{-3}$)	0.002 ($< 10^{-3}$)	0.046 (0.001)	0.032 ($< 10^{-3}$)	0.935 (0.003)	0.908 (0.018)	0.000	0.000
		CBPS-JI	-0.002 ($< 10^{-3}$)	0.002 ($< 10^{-3}$)	0.039 ($< 10^{-3}$)	0.031 ($< 10^{-3}$)	0.915 (0.004)	0.821 (0.017)	0.000	0.000
		CBPS-OI	-0.003 ($< 10^{-3}$)	0.001 ($< 10^{-3}$)	0.037 ($< 10^{-3}$)	0.029 ($< 10^{-3}$)	0.943 (0.003)	0.978 (0.019)	0.000	0.000
		CBPS-TLF	-0.003 ($< 10^{-3}$)	$< 10^{-3}$ ($< 10^{-3}$)	0.024 ($< 10^{-3}$)	0.019 ($< 10^{-3}$)	0.929 (0.004)	0.878 (0.017)	0.000	0.000
		EB	-0.002 ($< 10^{-3}$)	$< 10^{-3}$ ($< 10^{-3}$)	0.031 ($< 10^{-3}$)	0.025 ($< 10^{-3}$)	0.951 (0.003)	1.061 (0.021)	0.000	0.000
		KOM	-0.002 ($< 10^{-3}$)	$< 10^{-3}$ ($< 10^{-3}$)	0.029 ($< 10^{-3}$)	0.024 ($< 10^{-3}$)	0.952 (0.003)	1.060 (0.021)	0.000	0.000

Table 37: Scenario: $n = 2000$, proportion treated: high; complexity: low; treatment-effect level: none. Values are reported as estimate (Monte Carlo standard error), except for the missingness columns, which are reported without Monte Carlo standard error.

Estimand	Estimator	Method	Bias	Var	RMSE	MAE	Coverage	Var. ratio	Missingness	
									Point	SE
ATE	WLS	IPTW	0.006 ($< 10^{-3}$)	$< 10^{-3}$ ($< 10^{-3}$)	0.026 ($< 10^{-3}$)	0.021 ($< 10^{-3}$)	0.939 (0.003)	0.998 (0.020)	0.000	0.000
		CBPS-JI	0.003 ($< 10^{-3}$)	$< 10^{-3}$ ($< 10^{-3}$)	0.025 ($< 10^{-3}$)	0.020 ($< 10^{-3}$)	0.941 (0.003)	0.956 (0.019)	0.000	0.000
		CBPS-OI	0.008 ($< 10^{-3}$)	$< 10^{-3}$ ($< 10^{-3}$)	0.026 ($< 10^{-3}$)	0.021 ($< 10^{-3}$)	0.949 (0.003)	1.157 (0.023)	0.000	0.000
		CBPS-TLF	0.006 ($< 10^{-3}$)	$< 10^{-3}$ ($< 10^{-3}$)	0.025 ($< 10^{-3}$)	0.020 ($< 10^{-3}$)	0.847 (0.005)	0.556 (0.011)	0.000	0.000
		EB	0.001 ($< 10^{-3}$)	$< 10^{-3}$ ($< 10^{-3}$)	0.026 ($< 10^{-3}$)	0.021 ($< 10^{-3}$)	0.969 (0.002)	1.252 (0.025)	0.000	0.000
		KOM	0.005 ($< 10^{-3}$)	$< 10^{-3}$ ($< 10^{-3}$)	0.024 ($< 10^{-3}$)	0.019 ($< 10^{-3}$)	0.958 (0.003)	1.181 (0.023)	0.000	0.000
	DR	IPTW	$< 10^{-3}$ ($< 10^{-3}$)	$< 10^{-3}$ ($< 10^{-3}$)	0.025 ($< 10^{-3}$)	0.020 ($< 10^{-3}$)	0.944 (0.003)	0.974 (0.020)	0.000	0.000
		CBPS-JI	$< 10^{-3}$ ($< 10^{-3}$)	$< 10^{-3}$ ($< 10^{-3}$)	0.025 ($< 10^{-3}$)	0.020 ($< 10^{-3}$)	0.941 (0.003)	0.946 (0.019)	0.000	0.000
		CBPS-OI	$< 10^{-3}$ ($< 10^{-3}$)	$< 10^{-3}$ ($< 10^{-3}$)	0.024 ($< 10^{-3}$)	0.019 ($< 10^{-3}$)	0.945 (0.003)	0.992 (0.019)	0.000	0.000
		CBPS-TLF	$< 10^{-3}$ ($< 10^{-3}$)	$< 10^{-3}$ ($< 10^{-3}$)	0.025 ($< 10^{-3}$)	0.020 ($< 10^{-3}$)	0.858 (0.005)	0.553 (0.011)	0.000	0.000
		EB	$< 10^{-3}$ ($< 10^{-3}$)	$< 10^{-3}$ ($< 10^{-3}$)	0.024 ($< 10^{-3}$)	0.019 ($< 10^{-3}$)	0.945 (0.003)	0.992 (0.019)	0.000	0.000
		KOM	$< 10^{-3}$ ($< 10^{-3}$)	$< 10^{-3}$ ($< 10^{-3}$)	0.024 ($< 10^{-3}$)	0.019 ($< 10^{-3}$)	0.945 (0.003)	0.992 (0.019)	0.000	0.000
ATT	WLS	IPTW	0.007 ($< 10^{-3}$)	$< 10^{-3}$ ($< 10^{-3}$)	0.029 ($< 10^{-3}$)	0.024 ($< 10^{-3}$)	0.932 (0.004)	0.991 (0.020)	0.000	0.000
		CBPS-JI	0.004 ($< 10^{-3}$)	$< 10^{-3}$ ($< 10^{-3}$)	0.028 ($< 10^{-3}$)	0.022 ($< 10^{-3}$)	0.940 (0.003)	0.955 (0.019)	0.000	0.000
		CBPS-OI	0.009 ($< 10^{-3}$)	$< 10^{-3}$ ($< 10^{-3}$)	0.029 ($< 10^{-3}$)	0.023 ($< 10^{-3}$)	0.941 (0.003)	1.128 (0.023)	0.000	0.000
		CBPS-TLF	0.006 ($< 10^{-3}$)	$< 10^{-3}$ ($< 10^{-3}$)	0.028 ($< 10^{-3}$)	0.022 ($< 10^{-3}$)	0.829 (0.005)	0.521 (0.010)	0.000	0.000
		EB	0.003 ($< 10^{-3}$)	$< 10^{-3}$ ($< 10^{-3}$)	0.029 ($< 10^{-3}$)	0.023 ($< 10^{-3}$)	0.967 (0.003)	1.243 (0.025)	0.000	0.000
		KOM	0.006 ($< 10^{-3}$)	$< 10^{-3}$ ($< 10^{-3}$)	0.026 ($< 10^{-3}$)	0.021 ($< 10^{-3}$)	0.953 (0.003)	1.165 (0.023)	0.000	0.000
	DR	IPTW	$< 10^{-3}$ ($< 10^{-3}$)	$< 10^{-3}$ ($< 10^{-3}$)	0.028 ($< 10^{-3}$)	0.022 ($< 10^{-3}$)	0.939 (0.003)	0.966 (0.019)	0.000	0.000
		CBPS-JI	$< 10^{-3}$ ($< 10^{-3}$)	$< 10^{-3}$ ($< 10^{-3}$)	0.028 ($< 10^{-3}$)	0.022 ($< 10^{-3}$)	0.936 (0.003)	0.926 (0.019)	0.000	0.000
		CBPS-OI	$< 10^{-3}$ ($< 10^{-3}$)	$< 10^{-3}$ ($< 10^{-3}$)	0.027 ($< 10^{-3}$)	0.022 ($< 10^{-3}$)	0.943 (0.003)	0.980 (0.020)	0.000	0.000
		CBPS-TLF	$< 10^{-3}$ ($< 10^{-3}$)	$< 10^{-3}$ ($< 10^{-3}$)	0.028 ($< 10^{-3}$)	0.022 ($< 10^{-3}$)	0.844 (0.005)	0.517 (0.010)	0.000	0.000
		EB	$< 10^{-3}$ ($< 10^{-3}$)	$< 10^{-3}$ ($< 10^{-3}$)	0.029 ($< 10^{-3}$)	0.023 ($< 10^{-3}$)	0.944 (0.003)	1.000 (0.020)	0.000	0.000
		KOM	$< 10^{-3}$ ($< 10^{-3}$)	$< 10^{-3}$ ($< 10^{-3}$)	0.026 ($< 10^{-3}$)	0.021 ($< 10^{-3}$)	0.945 (0.003)	0.981 (0.019)	0.000	0.000

Table 38: Scenario: $n = 2000$, proportion treated: high; complexity: moderate; treatment-effect level: none. Values are reported as estimate (Monte Carlo standard error), except for the missingness columns, which are reported without Monte Carlo standard error.

Estimand	Estimator	Method	Bias	Var	RMSE	MAE	Coverage	Var. ratio	Missingness Point	Missingness SE
ATE	WLS	IPTW	0.013 ($< 10^{-3}$)	0.001 ($< 10^{-3}$)	0.038 ($< 10^{-3}$)	0.030 ($< 10^{-3}$)	0.891 (0.004)	0.971 (0.021)	0.000	0.000
		CBPS-JI	0.007 ($< 10^{-3}$)	$< 10^{-3}$ ($< 10^{-3}$)	0.031 ($< 10^{-3}$)	0.025 ($< 10^{-3}$)	0.905 (0.004)	0.846 (0.017)	0.000	0.000
		CBPS-OI	0.016 ($< 10^{-3}$)	$< 10^{-3}$ ($< 10^{-3}$)	0.036 ($< 10^{-3}$)	0.029 ($< 10^{-3}$)	0.908 (0.004)	1.242 (0.025)	0.000	0.000
		CBPS-TLF	0.043 ($< 10^{-3}$)	$< 10^{-3}$ ($< 10^{-3}$)	0.048 ($< 10^{-3}$)	0.043 ($< 10^{-3}$)	0.302 (0.006)	0.553 (0.011)	0.000	0.000
		EB	0.002 ($< 10^{-3}$)	$< 10^{-3}$ ($< 10^{-3}$)	0.027 ($< 10^{-3}$)	0.022 ($< 10^{-3}$)	0.983 (0.002)	1.613 (0.033)	0.000	0.000
		KOM	0.006 ($< 10^{-3}$)	$< 10^{-3}$ ($< 10^{-3}$)	0.026 ($< 10^{-3}$)	0.021 ($< 10^{-3}$)	0.973 (0.002)	1.470 (0.030)	0.000	0.000
	DR	IPTW	$< 10^{-3}$ ($< 10^{-3}$)	$< 10^{-3}$ ($< 10^{-3}$)	0.031 ($< 10^{-3}$)	0.024 ($< 10^{-3}$)	0.934 (0.004)	0.958 (0.020)	0.000	0.000
		CBPS-JI	$< 10^{-3}$ ($< 10^{-3}$)	$< 10^{-3}$ ($< 10^{-3}$)	0.029 ($< 10^{-3}$)	0.023 ($< 10^{-3}$)	0.917 (0.004)	0.828 (0.017)	0.000	0.000
		CBPS-OI	$< 10^{-3}$ ($< 10^{-3}$)	$< 10^{-3}$ ($< 10^{-3}$)	0.025 ($< 10^{-3}$)	0.020 ($< 10^{-3}$)	0.948 (0.003)	1.001 (0.021)	0.000	0.000
		CBPS-TLF	$< 10^{-3}$ ($< 10^{-3}$)	$< 10^{-3}$ ($< 10^{-3}$)	0.025 ($< 10^{-3}$)	0.020 ($< 10^{-3}$)	0.879 (0.005)	0.638 (0.013)	0.000	0.000
		EB	$< 10^{-3}$ ($< 10^{-3}$)	$< 10^{-3}$ ($< 10^{-3}$)	0.025 ($< 10^{-3}$)	0.020 ($< 10^{-3}$)	0.948 (0.003)	1.001 (0.021)	0.000	0.000
		KOM	$< 10^{-3}$ ($< 10^{-3}$)	$< 10^{-3}$ ($< 10^{-3}$)	0.025 ($< 10^{-3}$)	0.020 ($< 10^{-3}$)	0.948 (0.003)	1.001 (0.021)	0.000	0.000
ATT	WLS	IPTW	0.016 ($< 10^{-3}$)	0.002 ($< 10^{-3}$)	0.046 ($< 10^{-3}$)	0.037 ($< 10^{-3}$)	0.882 (0.005)	0.960 (0.020)	0.000	0.000
		CBPS-JI	0.008 ($< 10^{-3}$)	0.001 ($< 10^{-3}$)	0.038 ($< 10^{-3}$)	0.030 ($< 10^{-3}$)	0.909 (0.004)	0.883 (0.018)	0.000	0.000
		CBPS-OI	0.019 ($< 10^{-3}$)	0.002 ($< 10^{-3}$)	0.043 ($< 10^{-3}$)	0.035 ($< 10^{-3}$)	0.902 (0.004)	1.182 (0.024)	0.000	0.000
		CBPS-TLF	0.048 ($< 10^{-3}$)	$< 10^{-3}$ ($< 10^{-3}$)	0.053 ($< 10^{-3}$)	0.048 ($< 10^{-3}$)	0.284 (0.006)	0.612 (0.012)	0.000	0.000
		EB	0.007 ($< 10^{-3}$)	0.001 ($< 10^{-3}$)	0.033 ($< 10^{-3}$)	0.026 ($< 10^{-3}$)	0.977 (0.002)	1.601 (0.033)	0.000	0.000
		KOM	0.008 ($< 10^{-3}$)	$< 10^{-3}$ ($< 10^{-3}$)	0.030 ($< 10^{-3}$)	0.024 ($< 10^{-3}$)	0.971 (0.002)	1.477 (0.030)	0.000	0.000
	DR	IPTW	$< 10^{-3}$ ($< 10^{-3}$)	0.001 ($< 10^{-3}$)	0.038 ($< 10^{-3}$)	0.030 ($< 10^{-3}$)	0.934 (0.004)	0.959 (0.020)	0.000	0.000
		CBPS-JI	$< 10^{-3}$ ($< 10^{-3}$)	0.001 ($< 10^{-3}$)	0.036 ($< 10^{-3}$)	0.028 ($< 10^{-3}$)	0.912 (0.004)	0.804 (0.016)	0.000	0.000
		CBPS-OI	$< 10^{-3}$ ($< 10^{-3}$)	0.001 ($< 10^{-3}$)	0.035 ($< 10^{-3}$)	0.027 ($< 10^{-3}$)	0.939 (0.003)	1.010 (0.021)	0.000	0.000
		CBPS-TLF	$< 10^{-3}$ ($< 10^{-3}$)	$< 10^{-3}$ ($< 10^{-3}$)	0.029 ($< 10^{-3}$)	0.023 ($< 10^{-3}$)	0.886 (0.004)	0.656 (0.014)	0.000	0.000
		EB	$< 10^{-3}$ ($< 10^{-3}$)	0.001 ($< 10^{-3}$)	0.033 ($< 10^{-3}$)	0.026 ($< 10^{-3}$)	0.946 (0.003)	1.047 (0.021)	0.000	0.000
		KOM	$< 10^{-3}$ ($< 10^{-3}$)	$< 10^{-3}$ ($< 10^{-3}$)	0.031 ($< 10^{-3}$)	0.024 ($< 10^{-3}$)	0.949 (0.003)	1.027 (0.021)	0.000	0.000

Table 39: Scenario: $n = 2000$, proportion treated: high; complexity: high; treatment-effect level: none. Values are reported as estimate (Monte Carlo standard error), except for the missingness columns, which are reported without Monte Carlo standard error.

Estimand	Estimator	Method	Bias	Var	RMSE	MAE	Coverage	Var. ratio	Missingness Point	Missingness SE
ATE	WLS	IPTW	0.025 (0.001)	0.006 ($< 10^{-3}$)	0.081 (0.002)	0.062 ($< 10^{-3}$)	0.747 (0.006)	0.610 (0.024)	0.000	0.000
		CBPS-JI	0.012 ($< 10^{-3}$)	0.002 ($< 10^{-3}$)	0.048 ($< 10^{-3}$)	0.039 ($< 10^{-3}$)	0.795 (0.006)	0.510 (0.010)	0.000	0.000
		CBPS-OI	0.030 ($< 10^{-3}$)	0.003 ($< 10^{-3}$)	0.066 ($< 10^{-3}$)	0.055 ($< 10^{-3}$)	0.785 (0.006)	0.979 (0.019)	0.000	0.000
		CBPS-TLF	0.075 ($< 10^{-3}$)	$< 10^{-3}$ ($< 10^{-3}$)	0.078 ($< 10^{-3}$)	0.075 ($< 10^{-3}$)	0.020 (0.002)	0.364 (0.007)	0.000	0.000
		EB	0.004 ($< 10^{-3}$)	$< 10^{-3}$ ($< 10^{-3}$)	0.032 ($< 10^{-3}$)	0.025 ($< 10^{-3}$)	0.991 (0.001)	2.091 (0.043)	0.000	0.000
		KOM	0.008 ($< 10^{-3}$)	0.001 ($< 10^{-3}$)	0.033 ($< 10^{-3}$)	0.026 ($< 10^{-3}$)	0.978 (0.002)	1.821 (0.037)	0.000	0.000
	DR	IPTW	-0.004 ($< 10^{-3}$)	0.004 ($< 10^{-3}$)	0.063 (0.003)	0.040 ($< 10^{-3}$)	0.921 (0.004)	0.859 (0.030)	0.000	0.000
		CBPS-JI	-0.003 ($< 10^{-3}$)	0.002 ($< 10^{-3}$)	0.043 ($< 10^{-3}$)	0.034 ($< 10^{-3}$)	0.822 (0.005)	0.523 (0.011)	0.000	0.000
		CBPS-OI	-0.003 ($< 10^{-3}$)	$< 10^{-3}$ ($< 10^{-3}$)	0.030 ($< 10^{-3}$)	0.024 ($< 10^{-3}$)	0.924 (0.004)	0.921 (0.019)	0.000	0.000
		CBPS-TLF	-0.003 ($< 10^{-3}$)	$< 10^{-3}$ ($< 10^{-3}$)	0.030 ($< 10^{-3}$)	0.024 ($< 10^{-3}$)	0.853 (0.005)	0.597 (0.012)	0.000	0.000
		EB	-0.003 ($< 10^{-3}$)	$< 10^{-3}$ ($< 10^{-3}$)	0.030 ($< 10^{-3}$)	0.024 ($< 10^{-3}$)	0.924 (0.004)	0.921 (0.019)	0.000	0.000
		KOM	-0.003 ($< 10^{-3}$)	$< 10^{-3}$ ($< 10^{-3}$)	0.030 ($< 10^{-3}$)	0.024 ($< 10^{-3}$)	0.924 (0.004)	0.921 (0.019)	0.000	0.000
ATT	WLS	IPTW	0.031 (0.001)	0.009 ($< 10^{-3}$)	0.100 (0.001)	0.079 ($< 10^{-3}$)	0.746 (0.006)	0.604 (0.019)	0.000	0.000
		CBPS-JI	0.014 ($< 10^{-3}$)	0.005 ($< 10^{-3}$)	0.071 ($< 10^{-3}$)	0.058 ($< 10^{-3}$)	0.887 (0.004)	1.666 (0.088)	0.000	0.005
		CBPS-OI	0.034 (0.001)	0.006 ($< 10^{-3}$)	0.086 ($< 10^{-3}$)	0.071 ($< 10^{-3}$)	0.788 (0.006)	0.913 (0.018)	0.000	0.000
		CBPS-TLF	0.078 ($< 10^{-3}$)	$< 10^{-3}$ ($< 10^{-3}$)	0.082 ($< 10^{-3}$)	0.078 ($< 10^{-3}$)	0.030 (0.002)	0.456 (0.009)	0.000	0.000
		EB	0.020 ($< 10^{-3}$)	0.002 ($< 10^{-3}$)	0.048 ($< 10^{-3}$)	0.038 ($< 10^{-3}$)	0.965 (0.003)	1.875 (0.038)	0.000	0.000
		KOM	0.012 ($< 10^{-3}$)	0.002 ($< 10^{-3}$)	0.043 ($< 10^{-3}$)	0.035 ($< 10^{-3}$)	0.972 (0.002)	1.787 (0.036)	0.000	0.000
	DR	IPTW	-0.005 (0.001)	0.006 ($< 10^{-3}$)	0.079 (0.003)	0.051 ($< 10^{-3}$)	0.919 (0.004)	0.867 (0.031)	0.000	0.000
		CBPS-JI	-0.003 ($< 10^{-3}$)	0.004 ($< 10^{-3}$)	0.063 ($< 10^{-3}$)	0.050 ($< 10^{-3}$)	0.853 (0.005)	1.136 (0.067)	0.000	0.006
		CBPS-OI	-0.004 ($< 10^{-3}$)	0.003 ($< 10^{-3}$)	0.057 ($< 10^{-3}$)	0.043 ($< 10^{-3}$)	0.924 (0.004)	0.920 (0.018)	0.000	0.000
		CBPS-TLF	-0.004 ($< 10^{-3}$)	0.001 ($< 10^{-3}$)	0.038 ($< 10^{-3}$)	0.030 ($< 10^{-3}$)	0.858 (0.005)	0.612 (0.012)	0.000	0.000
		EB	-0.003 ($< 10^{-3}$)	0.002 ($< 10^{-3}$)	0.045 ($< 10^{-3}$)	0.036 ($< 10^{-3}$)	0.931 (0.004)	0.970 (0.020)	0.000	0.000
		KOM	-0.004 ($< 10^{-3}$)	0.002 ($< 10^{-3}$)	0.043 ($< 10^{-3}$)	0.034 ($< 10^{-3}$)	0.933 (0.004)	0.968 (0.020)	0.000	0.000
