# Supplementary figures and images for: Choosing Covariate Balancing Methods for Causal Inference: Practical Insights From a Simulation Study
Source: Stat Med. 2026 Jul 8;45(15-17):e70672. doi: 10.1002/sim.70672 (PMC13346537; doi:10.1002/sim.70672)

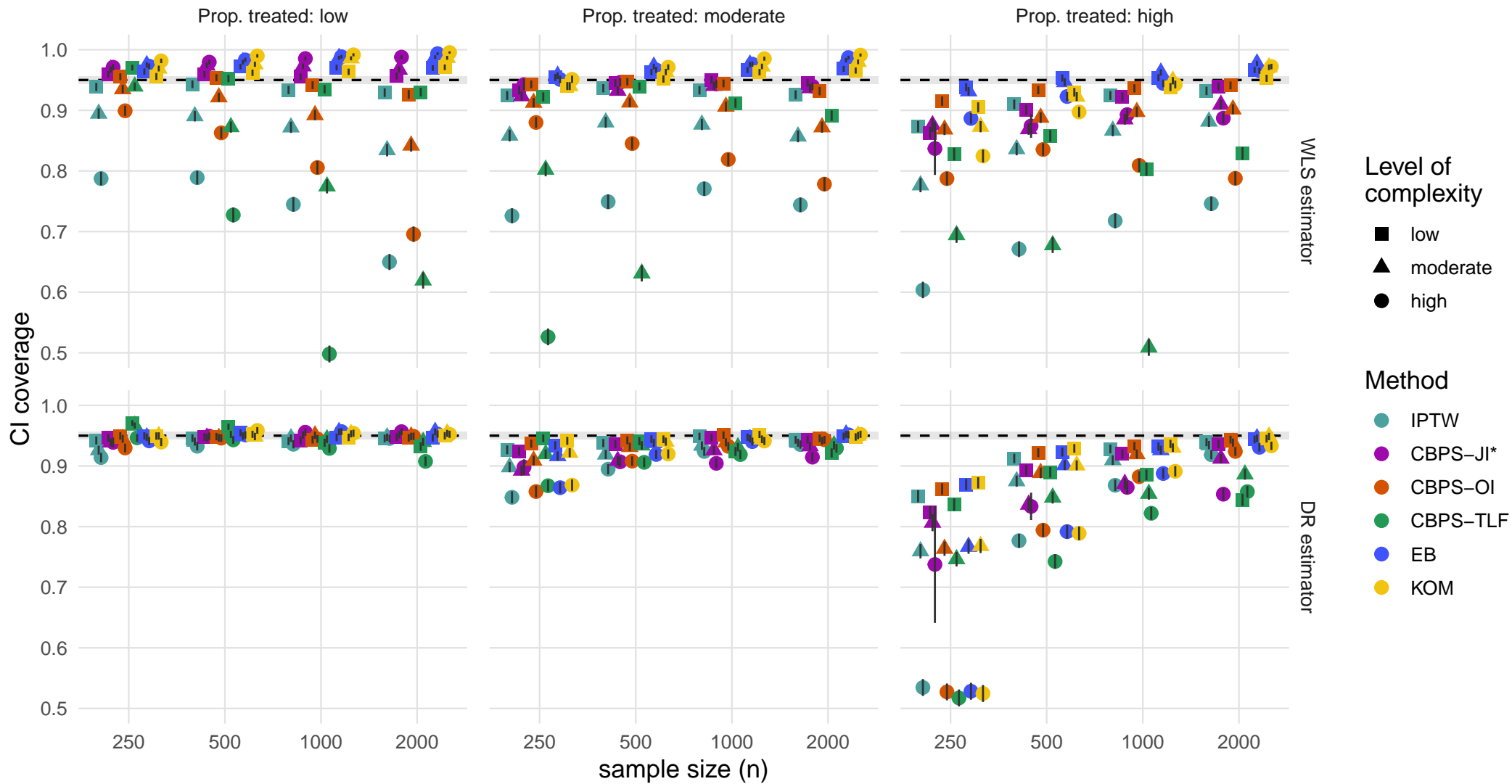

Supplement: Supplementary file 1 — Data S1: Supporting Information. [file SIM-45-0-s001.zip › sim70672-sup-0001-Supinfo/Peyrot_FigS10_CI_coverage-ATT.pdf]

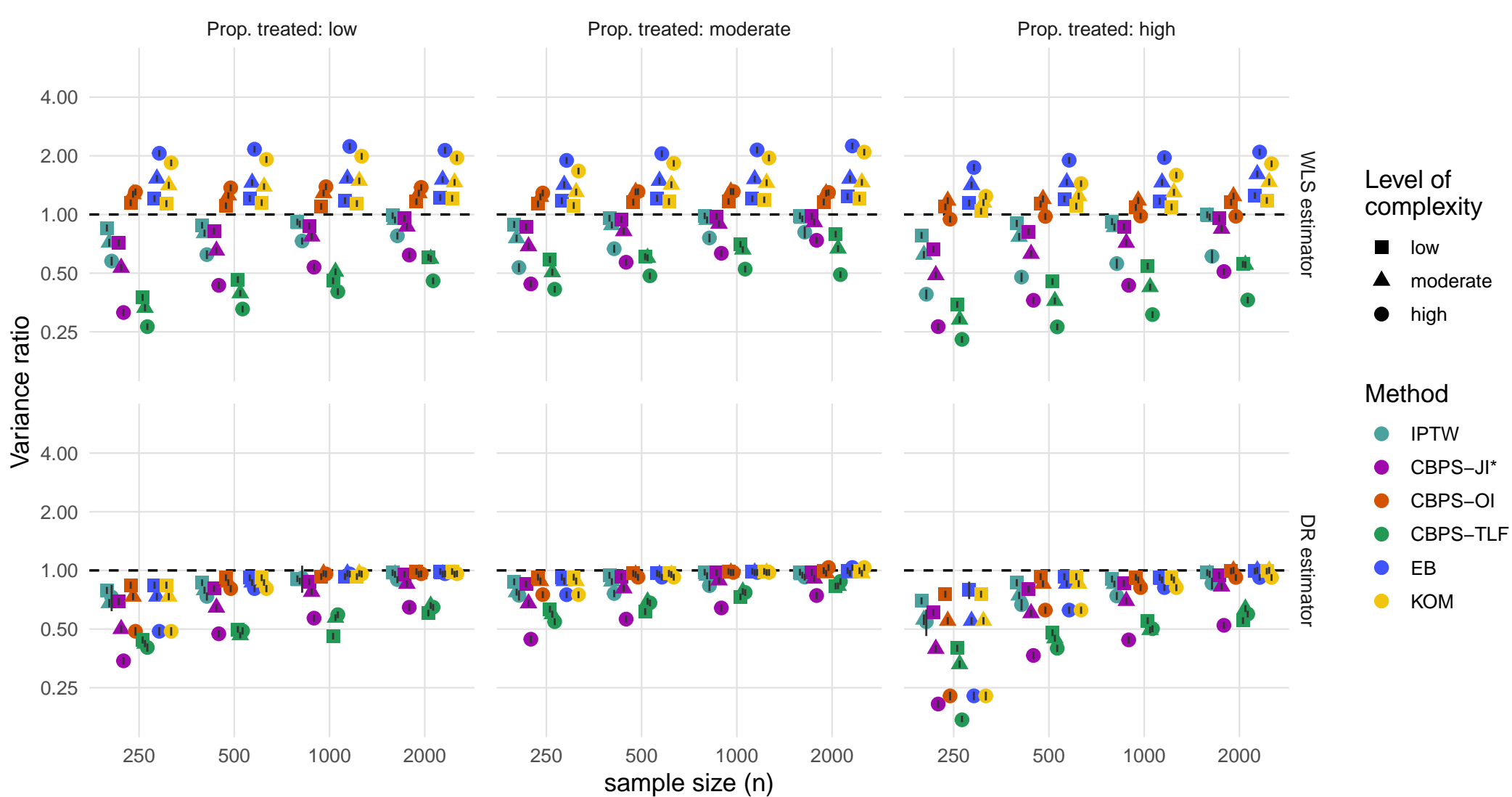

Supplement: Supplementary file 1 — Data S1: Supporting Information. [file SIM-45-0-s001.zip › sim70672-sup-0001-Supinfo/Peyrot_FigS11_variance_ratio-ATE.pdf]

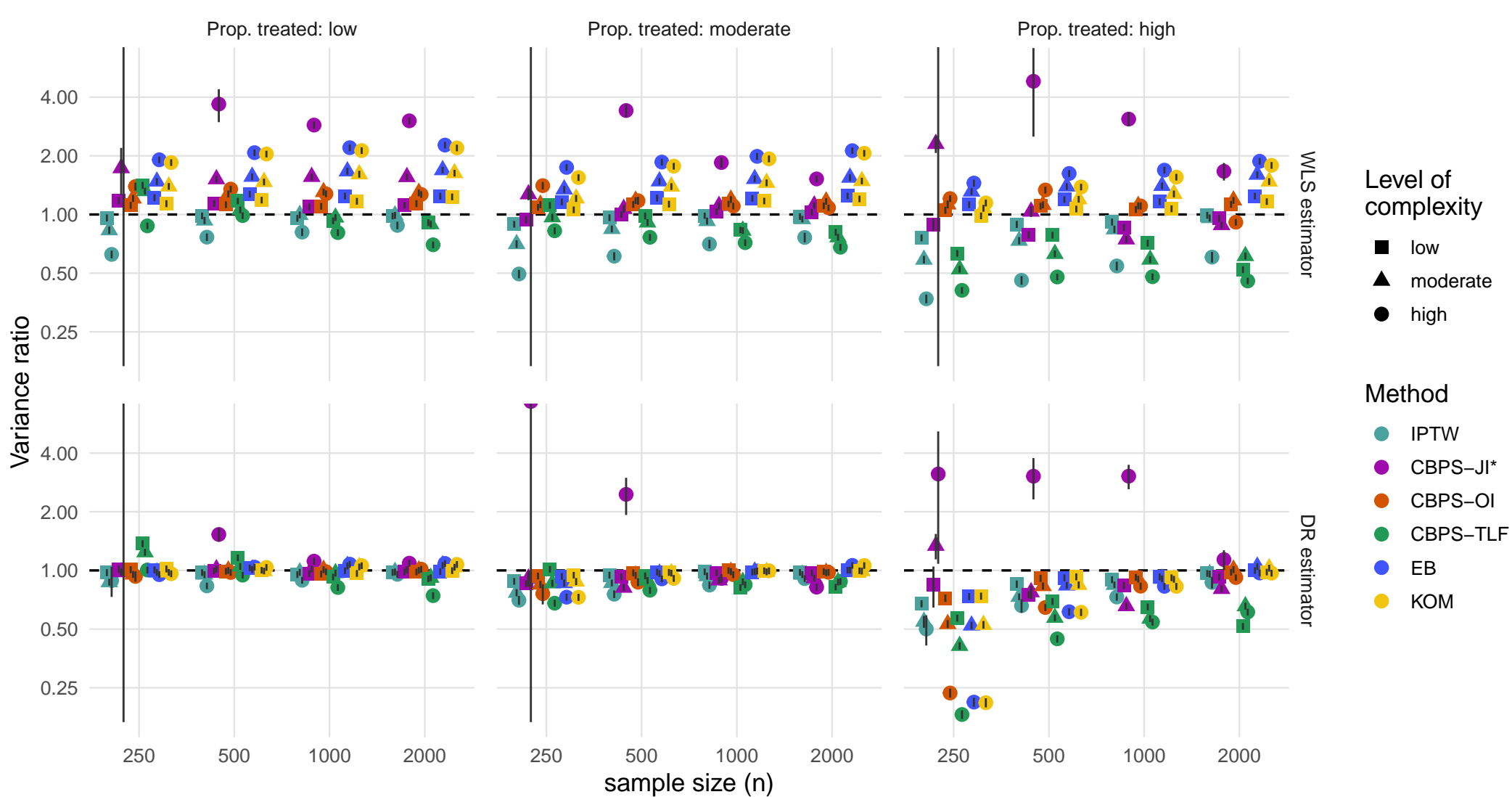

Supplement: Supplementary file 1 — Data S1: Supporting Information. [file SIM-45-0-s001.zip › sim70672-sup-0001-Supinfo/Peyrot_FigS12_variance_ratio-ATT.pdf]

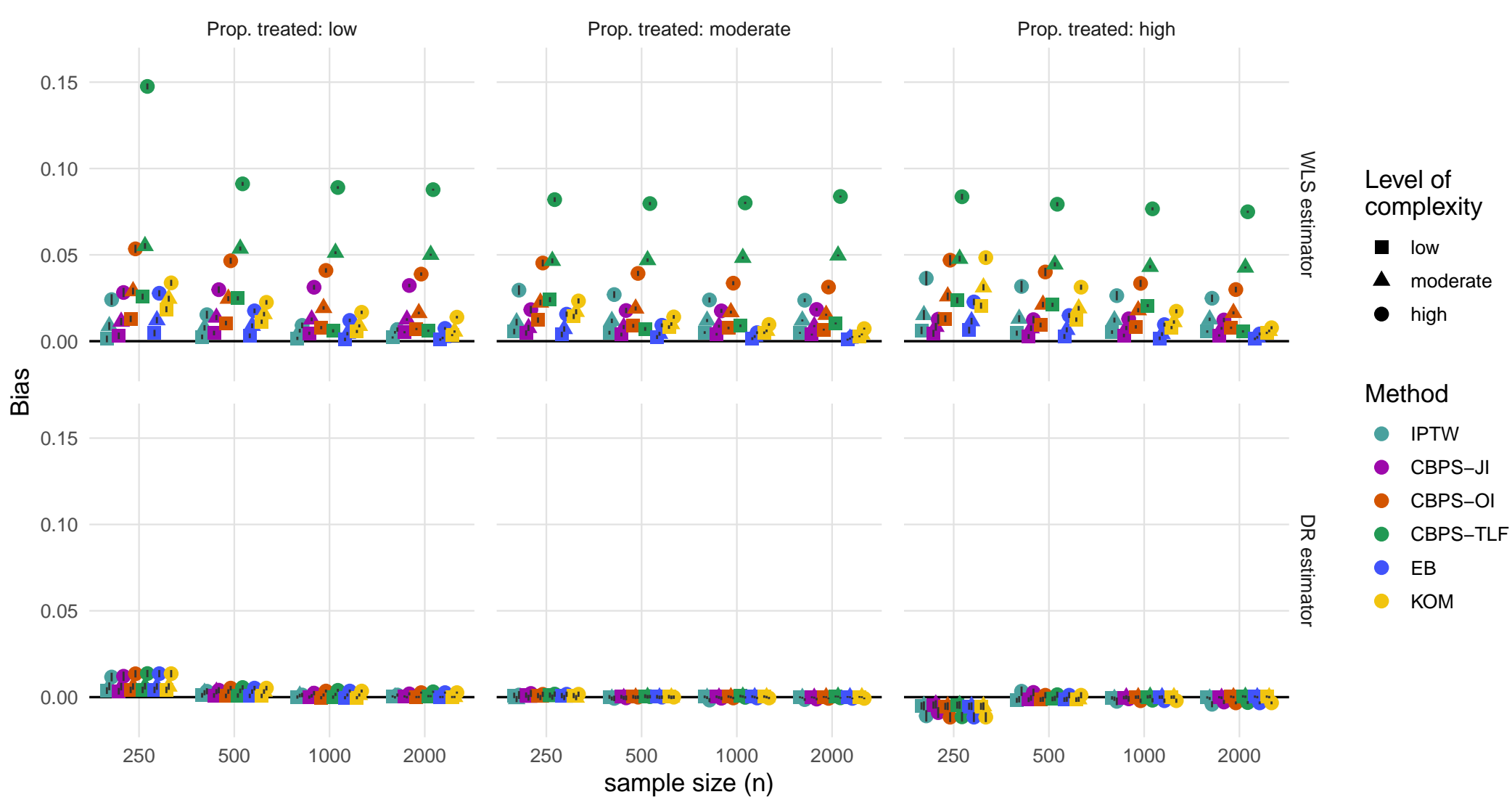

Supplement: Supplementary file 1 — Data S1: Supporting Information. [file SIM-45-0-s001.zip › sim70672-sup-0001-Supinfo/Peyrot_FigS1_bias-ATE.pdf]

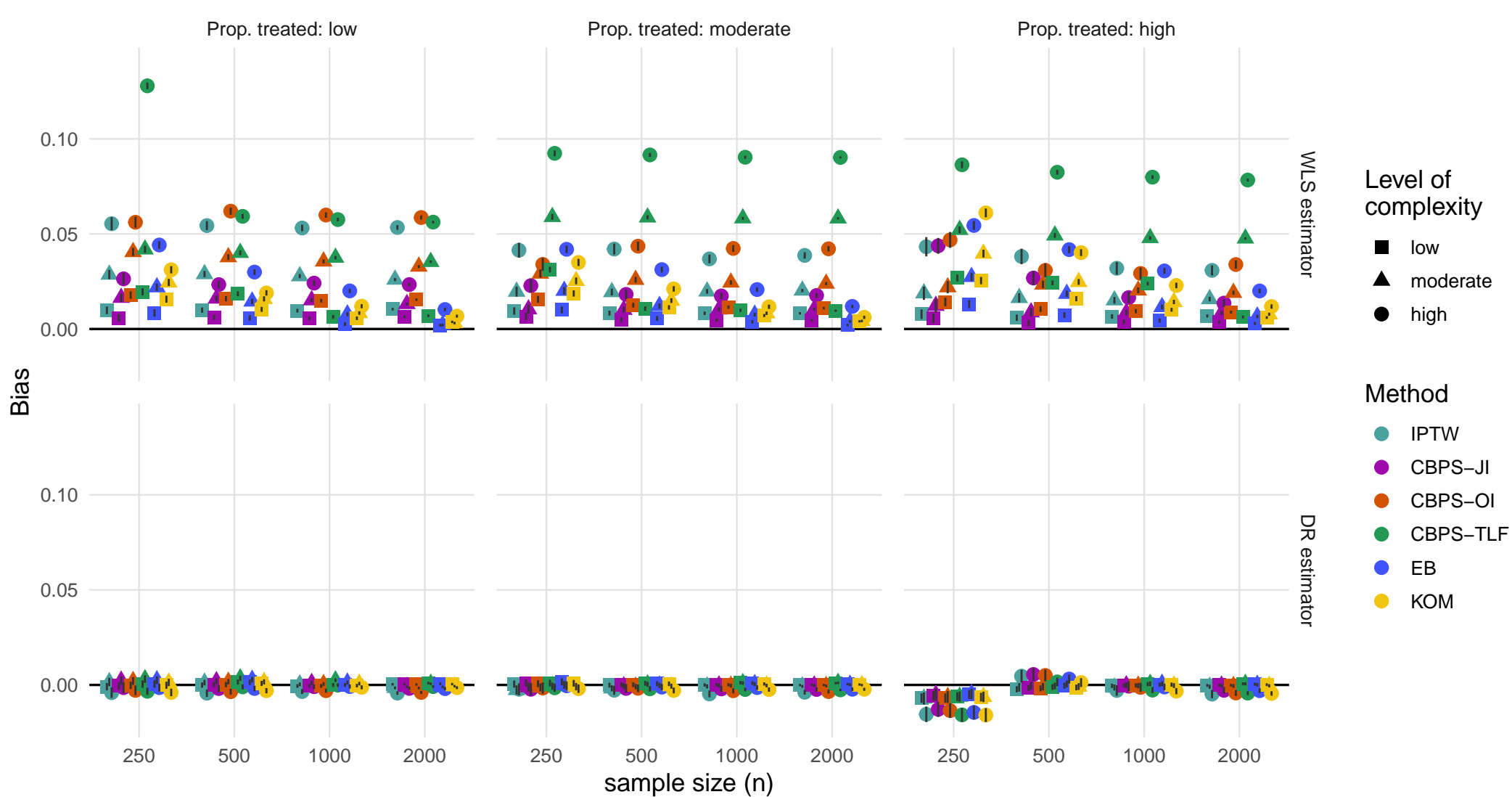

Supplement: Supplementary file 1 — Data S1: Supporting Information. [file SIM-45-0-s001.zip › sim70672-sup-0001-Supinfo/Peyrot_FigS2_bias-ATT.pdf]

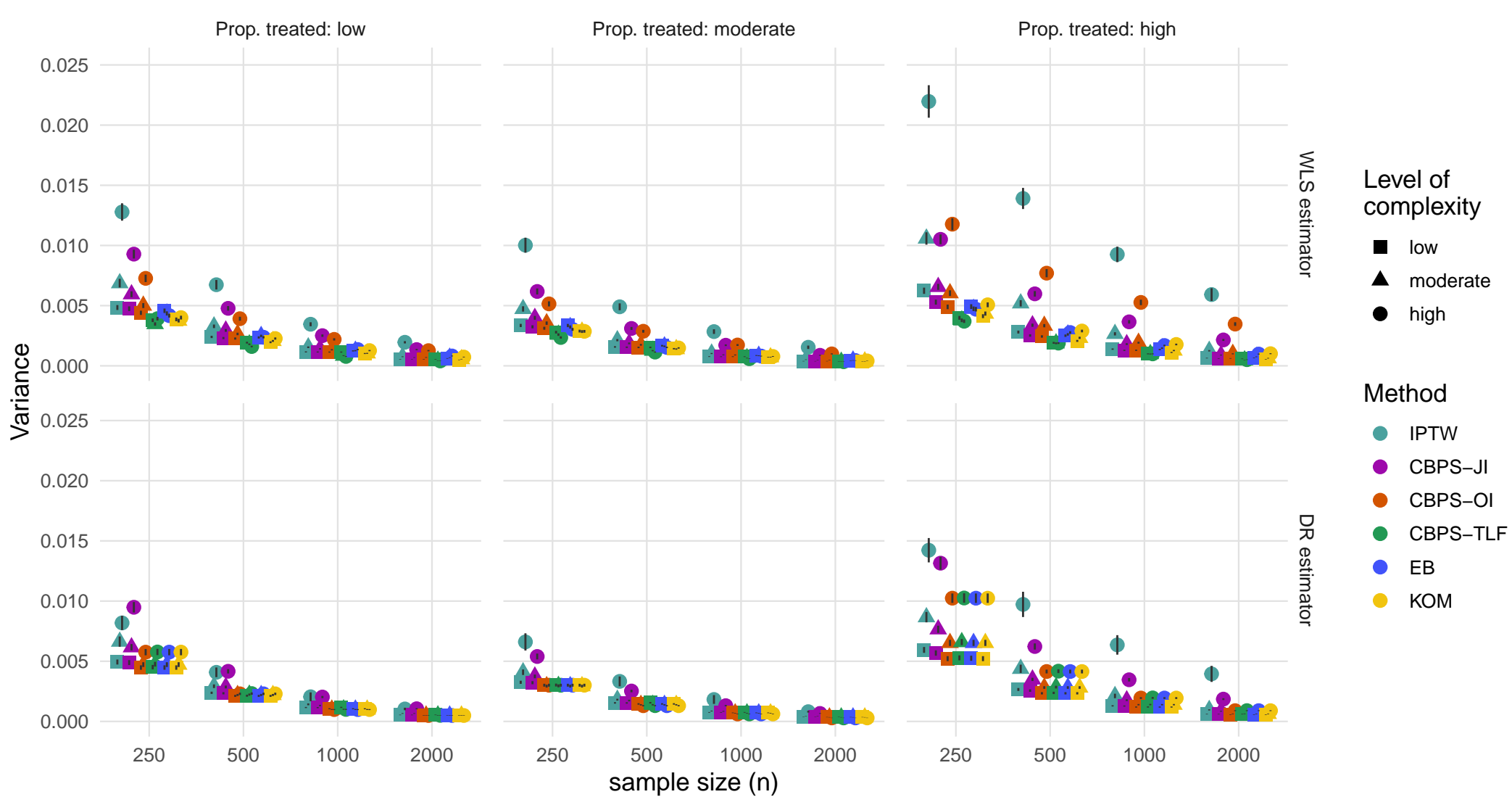

Supplement: Supplementary file 1 — Data S1: Supporting Information. [file SIM-45-0-s001.zip › sim70672-sup-0001-Supinfo/Peyrot_FigS3_variance-ATE.pdf]

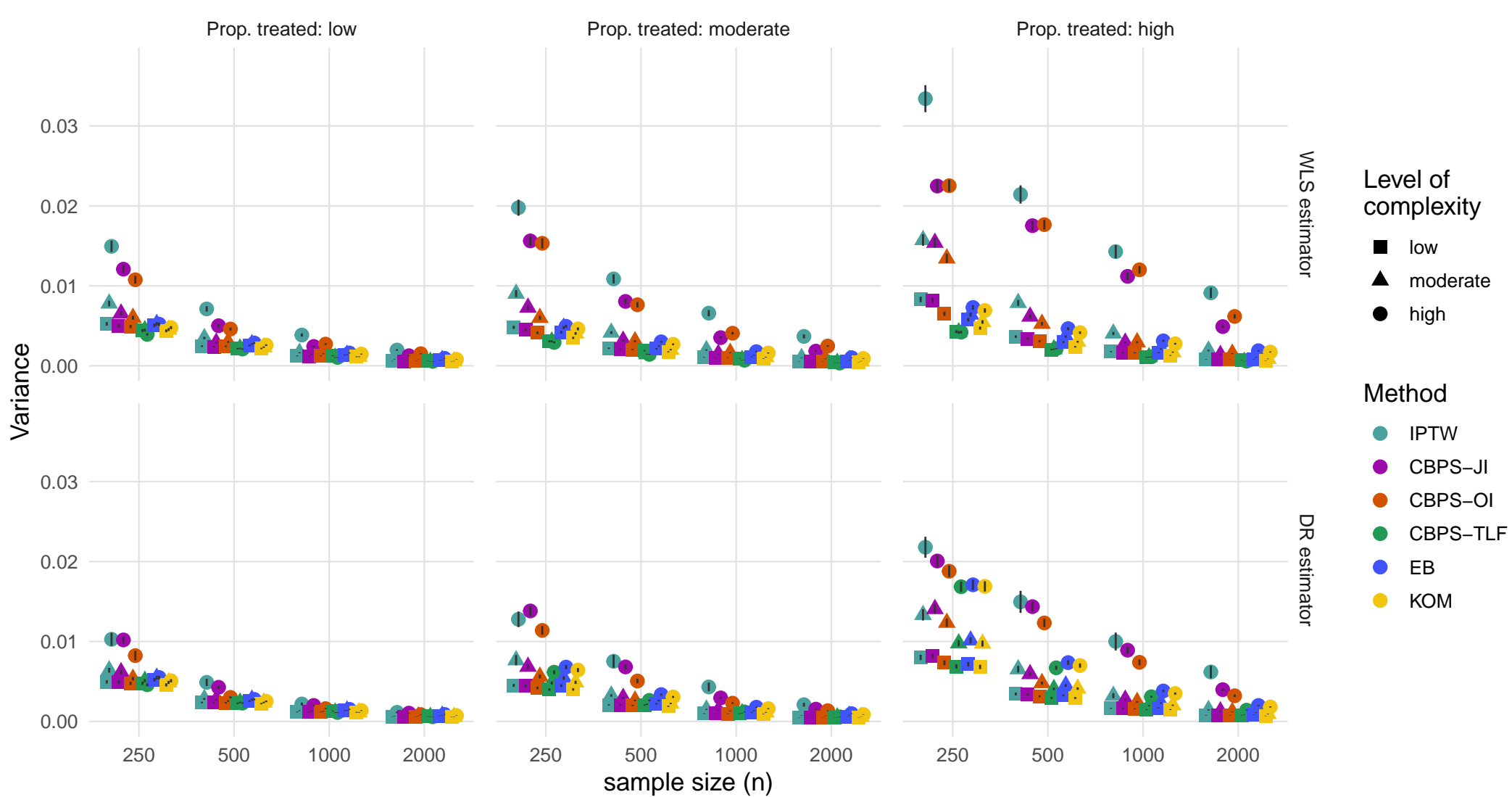

Supplement: Supplementary file 1 — Data S1: Supporting Information. [file SIM-45-0-s001.zip › sim70672-sup-0001-Supinfo/Peyrot_FigS4_variance-ATT.pdf]

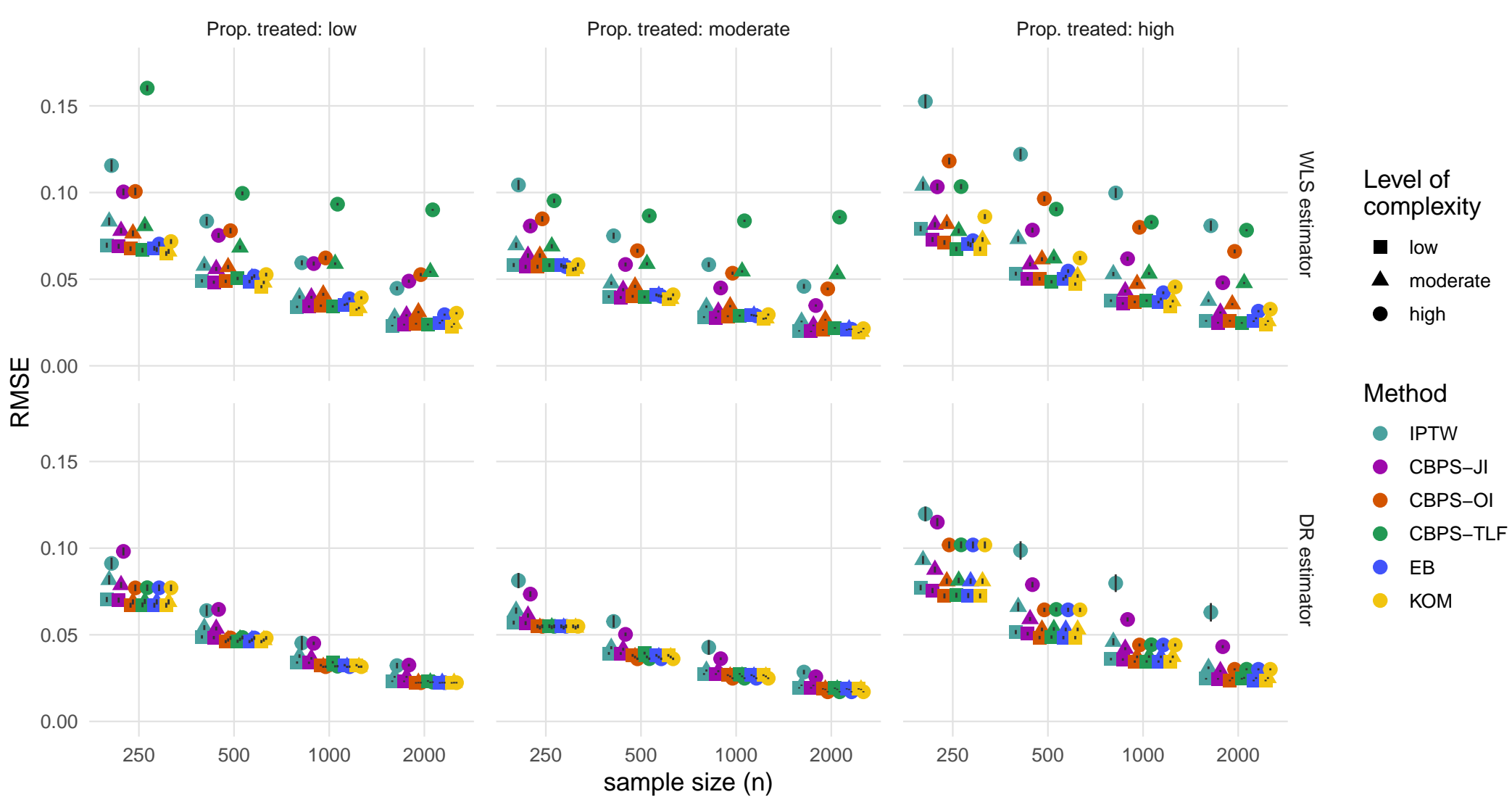

Supplement: Supplementary file 1 — Data S1: Supporting Information. [file SIM-45-0-s001.zip › sim70672-sup-0001-Supinfo/Peyrot_FigS5_rmse-ATE.pdf]

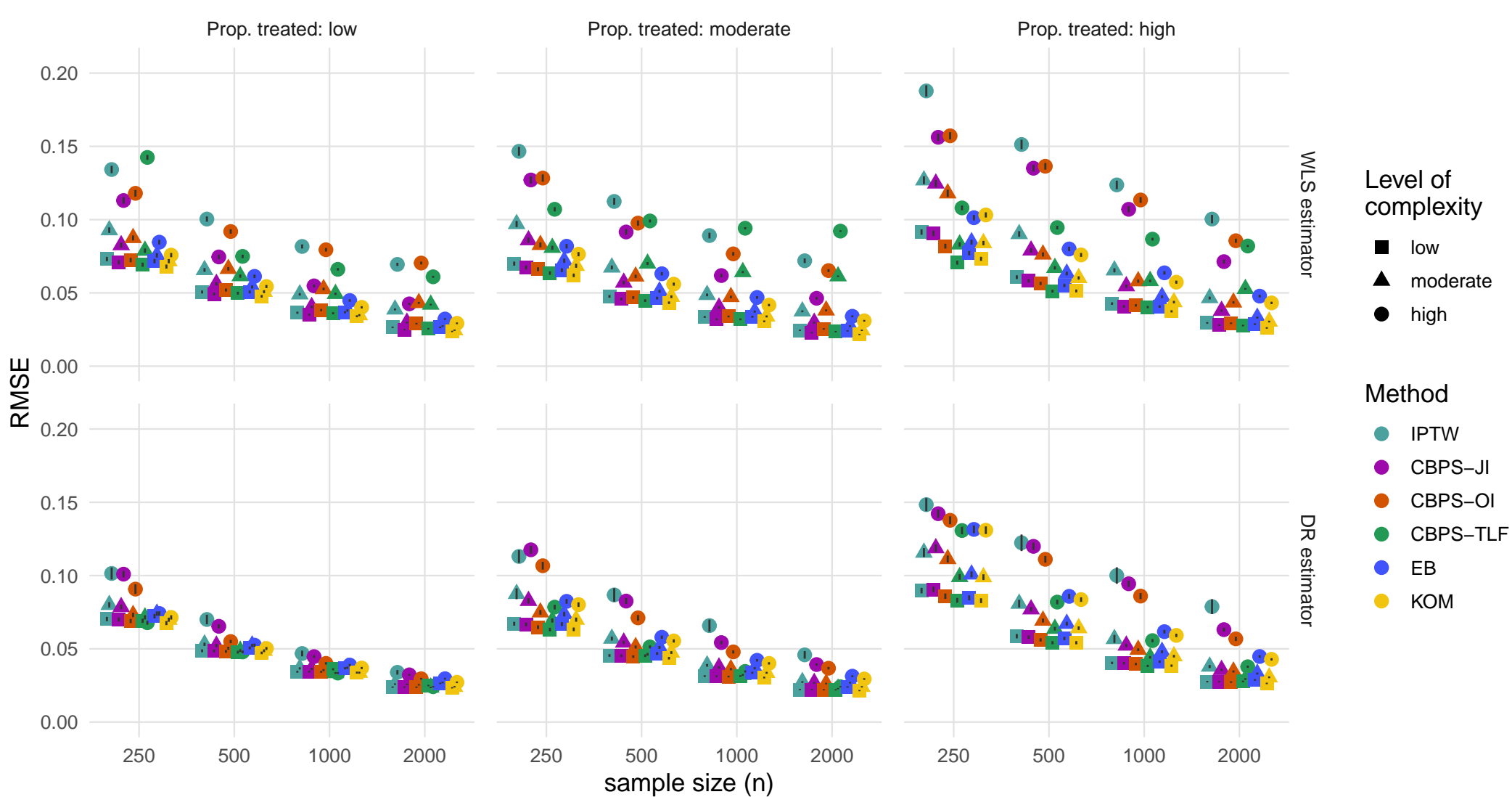

Supplement: Supplementary file 1 — Data S1: Supporting Information. [file SIM-45-0-s001.zip › sim70672-sup-0001-Supinfo/Peyrot_FigS6_rmse-ATT.pdf]

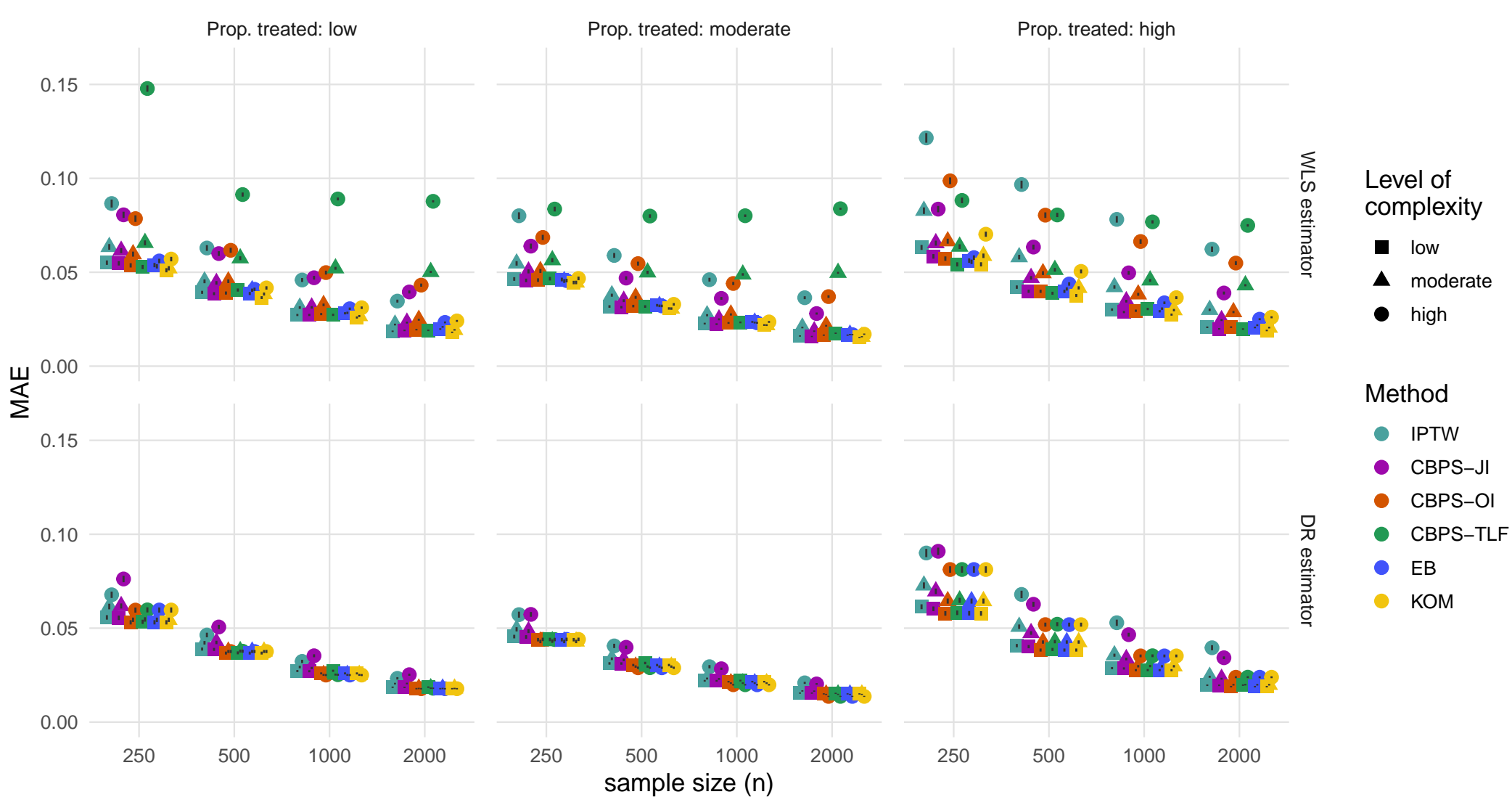

Supplement: Supplementary file 1 — Data S1: Supporting Information. [file SIM-45-0-s001.zip › sim70672-sup-0001-Supinfo/Peyrot_FigS7_mae-ATE.pdf]

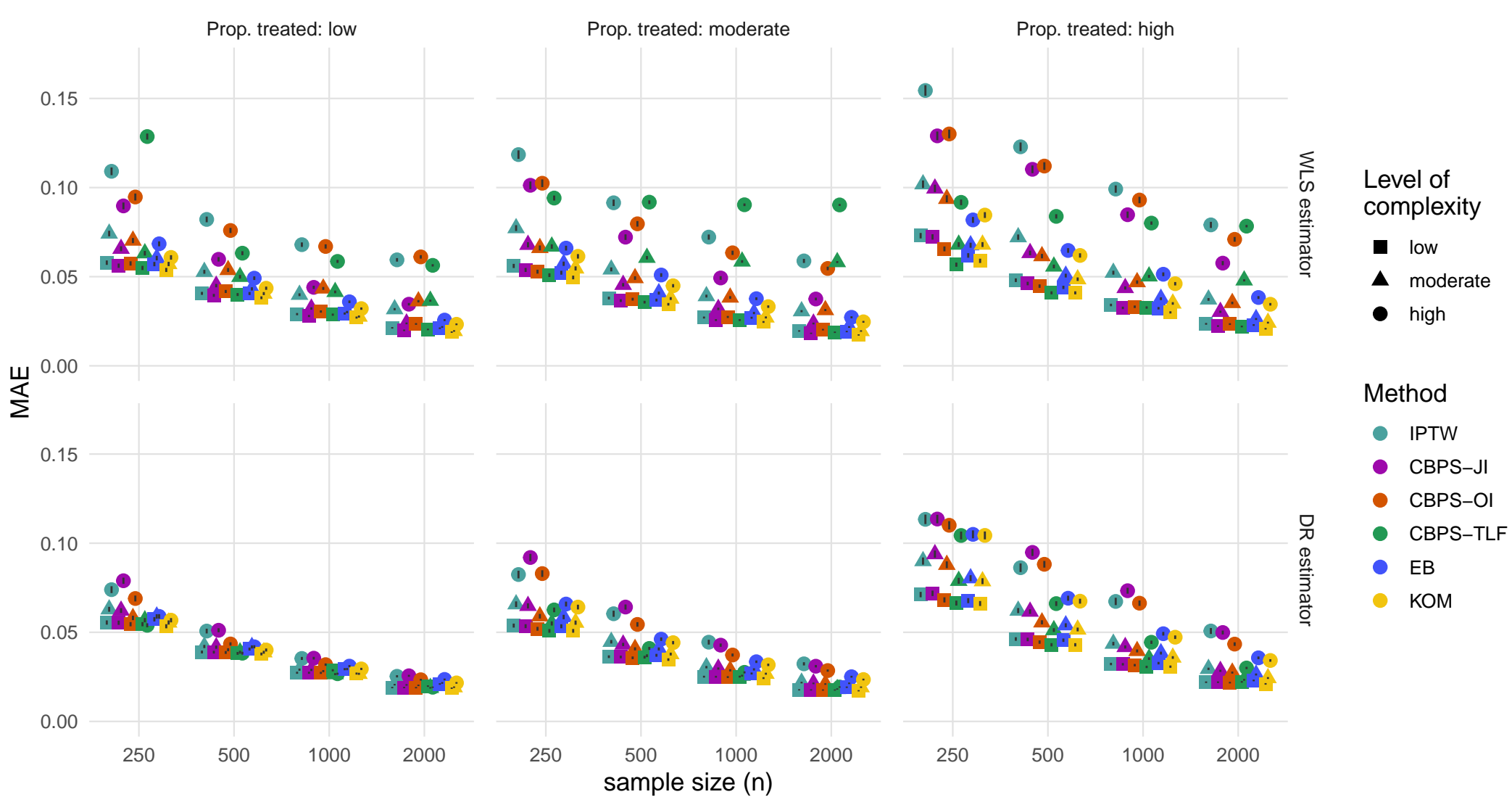

Supplement: Supplementary file 1 — Data S1: Supporting Information. [file SIM-45-0-s001.zip › sim70672-sup-0001-Supinfo/Peyrot_FigS8_mae-ATT.pdf]

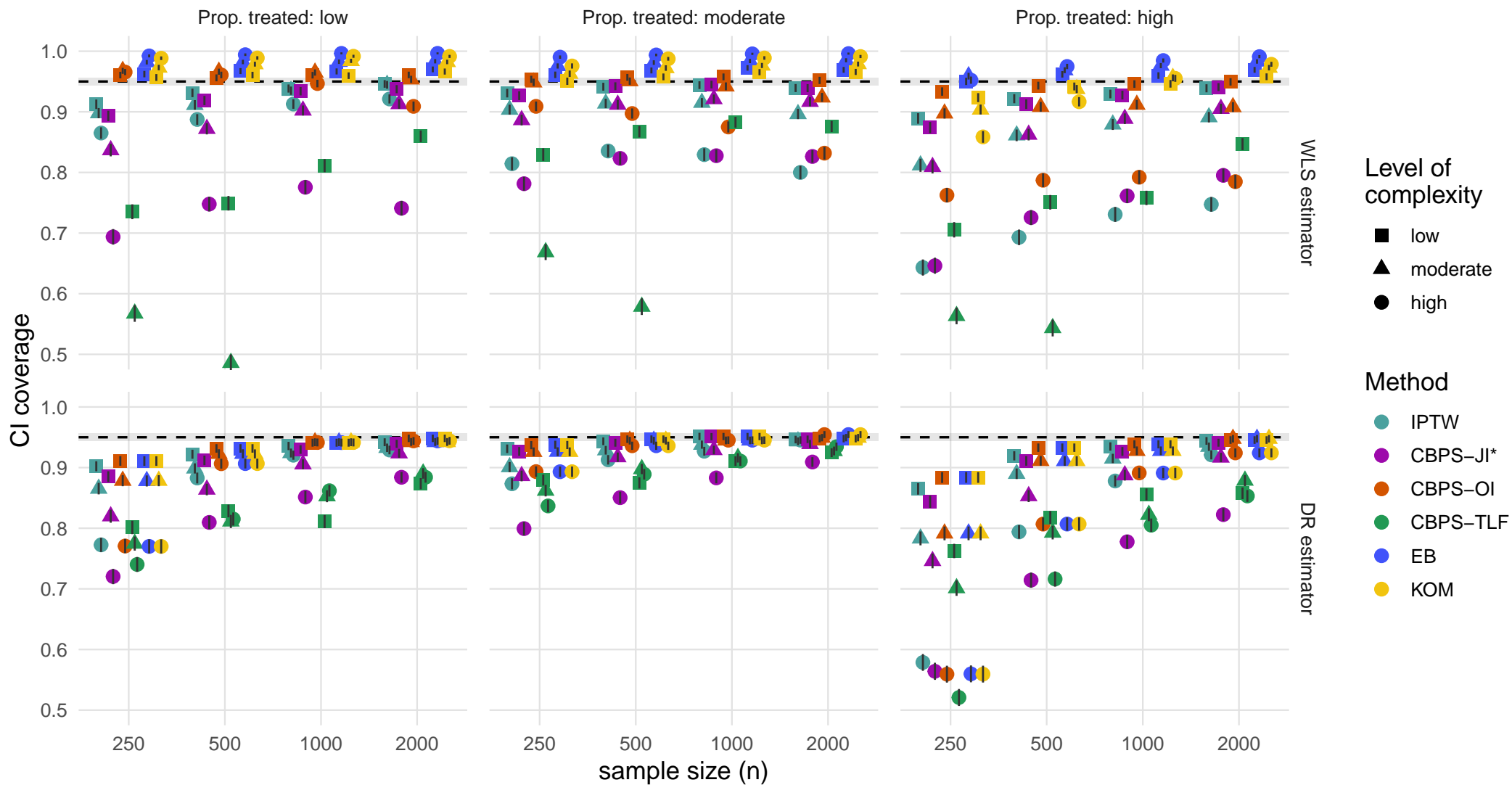

Supplement: Supplementary file 1 — Data S1: Supporting Information. [file SIM-45-0-s001.zip › sim70672-sup-0001-Supinfo/Peyrot_FigS9_CI_coverage-ATE.pdf]
